# Supplementary material for: Cognitive-bias modification intervention to improve physical activity in patients following a rehabilitation programme: protocol for the randomised controlled IMPACT trial
Source: BMJ Open. 2021 Sep 21;11(9):e053845. doi: 10.1136/bmjopen-2021-053845 (PMC8458354; doi:10.1136/bmjopen-2021-053845)
Supplement: Supplementary data [file bmjopen-2021-053845supp001.pdf]

Hôpitaux Universitaires Genève

UNIVERSITÉ DE GENÈVE
SWISS CENTER FOR AFFECTIVE SCIENCES

Titre de l'étude
L'étude IMPACT. Promouvoir l'activité physique des patients en reprogrammant leurs réactions automatiques envers l'activité physique à l'aide de jeux sérieux sur ordinateur.

Cette étude est organisée par : Les Hôpitaux Universitaires de Genève et le Centre Interfacultaire en Sciences Affectives (CISA) de l'Université de Genève.

Madame, Monsieur,

Nous vous proposons de participer à notre projet de recherche. Cette feuille d'information décrit le projet de recherche, d'abord dans une version courte (résumé), comme s'il s'agissait d'une table de matières, puis dans une version longue (version détaillée).

Résumé

1	**Objectifs de l'étude**<br>Par la présente, nous vous proposons de participer à notre étude clinique **IMPACT**. Cette étude concerne toutes les personnes qui sont qui sont hospitalisées au 3DK dans le service de médecine interne & réadaptation de l'Hôpital Beau-Séjour. Nous effectuons cette étude pour tester l'efficacité d'une nouvelle intervention visant à promouvoir l'activité physique chez des patients.
2	**Sélection des personnes**<br>La participation est ouverte à toutes les personnes hospitalisées au 3DK nécessitant des traitements de réhabilitation.
3	**Informations générales sur le projet**<br>Cette étude consiste en un programme d'entrainement qui fait appel à une tâche sur ordinateur dite de « jeu sérieux ». Vous serez répartis au hasard dans le groupe qui recevra le jeu sérieux supposé vous aider à devenir plus actif (c'est à dire, le groupe dit « intervention ») ou dans le groupe contrôle que recevra une forme de jeu sérieux factice (c'est à dire, le groupe dit « placebo »). Ni vous, ni la personne en charge de vous faire compléter le programme d'entrainement ne serez au courant du groupe dans lequel vous avez été inclut.<br><br>L'étude débutera début 2020 (après avoir obtenu l'accord du comité d'éthique) et se finira au mois de Janvier 2022 ; elle inclura un total de 250 patients.
4	**Déroulement pour les participants**<br><br><u>Procédure de sélection :</u><br>Dans un premier temps, le médecin responsable de l'étude vous indiquera si vous répondez aux critères d'inclusion de l'étude. La décision sur votre participation (ou non-participation) sera prise conjointement par le principal investigateur et le médecin responsable de l'étude. Un code d'identification créé de façon aléatoire vous sera fourni. Il vous sera demandé de conserver ce code pour les sessions du programme d'entrainement.<br><br><u>Intervention :</u><br>Vous serez invité à participer à une réunion au cours de laquelle vous seront expliqués les bienfaits sur votre santé et votre moral de l'activité physique. Vous aurez aussi l'occasion de discuter avec les médecins de vos appréhensions et barrières que vous pouvez ressentir à l'idée de faire de l'activité physique. Un plan d'activité physique adapté à votre pathologie et à vos attentes vous sera fourni par les professionnels de la santé qui vous prendront en charge. De plus, durant votre séjour, vous serez équipé d'une montre qui

	vous permettra d'avoir un retour d'information en continue sur votre niveau d'activité physique et de sédentarité (par exemple, nombre de pas, temps passé assis) au cours de votre programme de réhabilitation.<br><br>L'étude prévoit 15 séances (durée max. de chaque séance 15 min) réparties 3 semaines et au cours desquelles il vous sera demandé de compléter le jeu sérieux. Le groupe intervention recevra la version du jeu sérieux supposé vous aider à devenir plus actif. Le groupe contrôle recevra la version du jeu sérieux factice.<br><br>Pendant toute la durée de l'intervention, vos comportements d'activité physique et de sédentarité seront mesurés en continu à l'aide de la montre qui vous aura été distribuée. À chaque début de semaine, il vous sera aussi demandé de remplir à un questionnaire (durée max. 20 min). Les questions posées nous permettrons de connaître l'évolution de vos capacités physiques et de votre bien être.
5	**Bénéfices pour les participants**<br>La participation à l'intervention devrait vous aider à adopter un style de vie plus actif, et ainsi devrait s'accompagner de bienfaits sur votre santé et l'évolution de vos handicaps.
6	**Droits des participants**<br>Vous êtes libre d'accepter ou de refuser de participer à l'étude. Si vous décidez de ne pas participer, cela ne changera rien à votre prise en charge médicale. Vous n'avez pas à justifier vos décisions.
7	**Obligations des participants**<br>Si vous décidez de participer à l'étude, vous devrez accepter de suivre les instructions et de vous conformer au plan de l'étude.
8	**Risques**<br>Il n'y a pas de risque immédiat associé à cette intervention.
9	**Autres possibilités de traitement**<br>Votre médecin vous conseillera sur les autres possibilités concernant votre traitement.
10	**Découvertes**<br>Toute découverte survenant durant l'étude et pertinente pour votre santé vous sera communiquée. Si vous ne souhaitez pas obtenir ce type d'information, veuillez en avisez le médecin-investigateur.
11	**Confidentialité des données et des échantillons**<br>Nous enregistrerons vos données personnelles (sans géolocalisation). Si vous y consentez (consentement séparé), les données pourront être exploitées dans de futurs projets de recherche. Nous respectons toutes les dispositions légales relatives à la protection des données. Toutes les personnes impliquées sont soumises au secret professionnel. Vos données personnelles et médicales sont protégées et utilisées sous une forme codée.
12	**Retrait de l'étude**<br>Vous pouvez à tout moment vous retirer du projet si vous le souhaitez, sans avoir à vous justifier. Les données recueillies jusque-là seront analysées malgré tout. Cependant, si vous le souhaitez vos données peuvent être détruites. Dans ce cas, toutes les données ainsi que le formulaire de consentement seront détruits.
13	**Compensation des participants**<br>Si vous participez à cette étude, vous ne recevrez pour cela aucune compensation
14	**Réparation des dommages subis**<br>La responsabilité civile des Hôpitaux Universitaires de Genève couvre les dommages éventuels dans le cadre de l'étude<br>L'Université de Genève a souscrit une assurance auprès de AXA Winterthur pour pouvoir réparer les dommages sous sa responsabilité.
15	**Financement de l'étude**

Hôpitaux Universitaires Genève

UNIVERSITÉ DE GENÈVE
SWISS CENTER FOR AFFECTIVE SCIENCES

	L'étude est financée par le Fond National Suisse de la Recherche Scientifique et les fonds de service du service de médecine interne & réadaptation de l'Hôpital Beau-Séjour des Hôpitaux Universitaire de Genève
16	**Interlocuteur(s)**<br>Christophe Luthy, Prof.<br>4 Rue Gabrielle-Perret-Gentil, 1205 Genève<br>E-mail: Christophe.Luthy@hcuge.ch

17

Hôpitaux Universitaires Genève

UNIVERSITÉ DE GENÈVE
SWISS CENTER FOR AFFECTIVE SCIENCES

Information détaillée

1. Objectifs de l'étude

Cette étude doit nous permettre de tester l'efficacité d'une nouvelle intervention visant à promouvoir l'activité physique chez des patients. Plus spécifiquement, nous voulons savoir si un programme d'entrainement basés sur un jeu dit « sérieux » est efficace pour permettre aux patients d'être davantage actif.

2. Sélection des personnes pouvant participer à l'étude

La participation est ouverte à toutes les personnes hospitalisées au 3DK dans le service de médecine interne & réadaptation de l'Hôpital Beau-Séjour et nécessitant des traitements de réhabilitation. Elle est en revanche fermée aux personnes pour qui une réhabilitation n'est pas indiquée.

3. Informations générales sur l'étude

- Cette étude a pour but de tester l'efficacité d'une nouvelle intervention visant à promouvoir l'activité physique chez des patients.

- Cette étude est basée en **Suisse** et toute l'intervention se déroulera au 3DK.

- L'intervention consiste en un programme d'entrainement qui fait appel à une tâche sur ordinateur dit de « jeu sérieux ». Le **programme d'entrainement** sera constitué de 15 séances (durée max. de chaque séance 15 min) réparties 3 semaines et au cours desquelles il vous sera demandé de compléter le « jeu sérieux ».

- L'intervention se déroulera en plusieurs étapes. Dans un premier temps, le médecin responsable de l'étude vous indiquera si vous répondez aux critères d'inclusions de l'étude. Ensuite, vous serez invité à participer à une **réunion** au cours de laquelle il vous sera expliqué les bienfaits de l'activité physique. Pendant cette réunion, une montre permettant de mesurer en continue votre activité physique vous sera prêtée. Vous serez ensuite **aléatoirement** réparti dans le groupe qui recevra le jeu sérieux supposé vous aider à devenir plus actif (c'est à dire, le **groupe dit** « **intervention** ») ou dans le groupe contrôle que recevra une forme de jeu sérieux factice (c'est à dire, **le groupe dit** « **placebo** »). Ni vous, ni la personne en charge de vous faire compléter le programme d'entrainement ne serez au courant du groupe dans lequel vous avez été inclus. Au cours de votre programme de réhabilitation, il vous sera aussi demandé de remplir chaque début de semaine à un **questionnaire** (durée max. 20 min) nous permettant de de connaître l'évolution de vos capacités physiques et de votre bien être.

- Au total, la durée de cette étude clinique sera de 2 ans et 250 participants seront inclus.

Nous effectuons cette étude dans le respect des prescriptions de la législation suisse. Nous suivons en outre l'ensemble des directives reconnues au niveau international. La commission cantonale d'éthique compétente a contrôlé et autorisé l'étude.

Vous trouverez aussi un descriptif de l'étude sur le site Internet de l'Office fédéral de la santé publique : www.kofam.ch (le numéro de registre SNCTP de l'étude sera indiqué ici).

4. Déroulement pour les participants

- Procédure de sélection (durée 20 minutes maximum) :

Dans un premier temps, le médecin responsable de l'étude vous indiquera si vous répondez aux critères d'inclusion de l'étude. La décision sur votre participation (ou non-participation) sera prise conjointement par le principal investigateur de l'étude et le médecin responsable de l'étude. Un code d'identification crée de façon aléatoire vous sera fourni. Il vous sera demandé de conserver ce code pour les sessions du programme d'entrainement.

- Intervention (15 sessions de 15 minutes maximum réparties sur 3 semaines) :

Vous serez invité à participer à une réunion au cours de laquelle vous seront expliqués les aspects positifs de l'activité physique. Vous aurez aussi l'occasion de discuter avec les médecins de vos appréhensions et barrières que vous pouvez ressentir à l'idée de faire de l'activité physique. Un plan d'activité physique adapté à votre pathologie et à vos attentes vous sera fourni par les professionnels de la santé qui vous prendront en charge. De plus, vous recevrez une montre qui vous permettra d'avoir un retour d'information en continue sur votre niveau d'activité physique et de sédentarité (par exemple, nombre de pas, temps passé assis) au cours de votre programme de réhabilitation.

L'étude prévoit 15 séances (durée max. de chaque séance 15 min) réparties 3 semaines et au cours desquelles il vous sera demandé de compléter le « jeu sérieux ». Le groupe intervention recevra la version du jeu sérieux supposé vous aider à devenir plus actif. Le groupe contrôle recevra la version du jeu sérieux factice.

Pendant toute la durée de l'intervention, vos comportements d'activité physique et de sédentarité seront mesurés en continu à l'aide de la montre qui vous aura été distribuée. À chaque début de semaine, il vous sera aussi demandé de remplir à un questionnaire (durée max. 20 min). Les questions posées nous permettrons de connaître l'évolution de vos capacités physiques et de votre bien être.

Il se peut que nous devions vous retirer de l'étude avant le terme prévu. Cette situation peut se produire si nous mettons en évidence une contre-indication. En pareil cas, nous vous proposerons pour votre propre sécurité de vous examiner une dernière fois. Votre médecin traitant sera informé de votre participation à l'étude.

5. Bénéfices pour les participants

Si vous participez à l'étude, cela pourra éventuellement vous aider à adopter un style de vie plus actif, et ainsi devrait s'accompagner de bienfaits sur votre santé et l'évolution de vos handicaps.

Les résultats de l'étude pourraient se révéler importants par la suite pour aider les personnes à être plus actif physiquement.

6. Droits des participants

Votre participation est entièrement libre. Si vous choisissez de ne pas participer ou si vous choisissez de participer et revenez sur votre décision pendant le déroulement de l'étude, vous n'aurez pas à justifier votre refus. Cela ne changera rien à votre prise en charge médicale habituelle. Si vous le souhaitez vos données pourront être détruites. Vous pouvez à tout moment poser toutes les questions nécessaires au sujet de l'étude. Veuillez vous adresser pour ce faire à la personne indiquée à la fin de la présente feuille d'information.

7. Obligations des participants

En tant que participant à l'étude, vous serez tenu :

- de suivre les instructions et de vous conformer au plan de l'étude, à savoir, participer à la première réunion d'information sur les bienfaits de l'activité physique, compléter les 15 séances du programme d'entrainement, porter une montre 24h/24h nous permettant de mesurer votre activité physique en continue, et remplir l'ensemble des questionnaires qui vous seront proposés au cours de l'intervention.

Cheval B, *et al. BMJ Open* 2021; 11:e053845. doi: 10.1136/bmjopen-2021-053845

8. Risques et contraintes pour les participants

- Il n'y a pas de risque immédiat associé à cette intervention.

9. Autres possibilités de traitement

Vous n'êtes pas tenu de participer à l'étude. Si vous décidez de ne pas y prendre part, il vous sera toujours possible de demander des informations aux médecins concernant les bienfaits de l'activité physique. Vous pourrez aussi participer à la réunion visant à vous expliquer les bienfaits de l'activité physique sans pour autant accepter de participer au programme d'entrainement.

10. Découvertes pendant l'étude

Le médecin-investigateur vous avisera pendant l'étude de toute nouvelle découverte susceptible d'influer sur les bénéfices de l'étude ou votre sécurité, et donc sur votre consentement à participer. Vous serez informé oralement et par écrit.

11. Confidentialité des données et des échantillons

Pour les besoins de l'étude, nous enregistrerons vos données personnelles et médicales. Seul un nombre limité de personnes peut consulter vos données sous une forme non codée, et exclusivement afin de pouvoir accomplir des tâches nécessaires au déroulement du projet. Les données recueillies à des fins de recherche sont codées lors de leur collecte. Le codage signifie que toutes les données permettant de vous identifier (p. ex. le nom, la date de naissance, etc.) sont remplacées par un code (ce code aléatoirement crée vous sera communiqué au début de l'étude. Seuls les responsables auront accès à la base de données permettant de faire le lien entre ce code et votre personne). Le code reste en permanence au sein de l'hôpital. Les personnes ne connaissant pas ce code ne peuvent pas lier ces données à votre personne. Dans le cas d'une publication, les données seront agrégées et personne ne pourra être individuellement identifié. Votre nom n'apparaîtra jamais sur Internet ou dans une publication. Parfois, les journaux scientifiques exigent la transmission de données individuelles (données brutes). Si des données individuelles devaient être transmises, elles seraient toujours codées et ne permettraient donc pas de vous identifier en tant que personne. Toutes les personnes impliquées dans l'étude de quelque manière que ce soit sont tenues au secret professionnel. Toutes les directives relatives à la protection des données sont respectées et vous avez à tout moment le droit de consulter vos données.

Durant son déroulement, l'étude peut faire l'objet d'inspections. Celles-ci peuvent être effectuées par la commission d'éthique qui s'est chargée de son contrôle initial et l'a autorisé, mais aussi être mandatées par l'organisme qui l'a initiée (le fond national suisse pour la recherche scientifique). Il se peut que le médecin-investigateur doive communiquer vos données personnelles et médicales pour les besoins de ces inspections. En cas de dommage, un représentant de l'assurance peut également être amené à consulter vos données. Toutes les personnes sont tenues au secret professionnel.

12. Retrait de d'étude

Vous pouvez à tout moment vous retirer de l'étude si vous le souhaitez, sans avoir besoin de vous justifier.

Les données personnelles recueillies jusque-là seront tout de même analysés, ceci afin de ne pas compromettre la valeur de l'étude dans son ensemble.

13. Compensation des participants

Si vous participez à ce projet, vous ne recevrez pour cela aucune rémunération.

Cheval B, *et al. BMJ Open* 2021; 11:e053845. doi: 10.1136/bmjopen-2021-053845

Hôpitaux Universitaires Genève

UNIVERSITÉ DE GENÈVE
SWISS CENTER FOR AFFECTIVE SCIENCES

14. Réparation des dommages subis

L'organisme ou l'entreprise (promoteur) qui a initié l'étude et est en charge de sa réalisation est responsable des dommages que vous pourriez subir en relation avec la substance à l'étude ou avec les activités de recherche (p.ex. examens). Les conditions et la procédure sont fixées par la loi. L'Université de Genève a conclu une assurance auprès de la compagnie AXA Winterthur pour être en mesure de réparer les dommages relevant de sa responsabilité. La responsabilité civile des Hôpitaux Universitaires de Genève couvre les dommages éventuels dans le cadre de l'étude

15. Financement de l'étude

L'étude est financée par le Fond National Suisse de la Recherche Scientifique les fonds de service du service de médecine interne & réadaptation de l'Hôpital Beau-Séjour des Hôpitaux Universitaire de Genève

16. Interlocuteur(s)

En cas de doute, de craintes ou d'urgences pendant ou après l'étude, vous pouvez vous adresser à tout moment à :

Prof. Christophe Luthy, 4 Rue Gabrielle-Perret-Gentil, 1205 Genève ; E-mail: Christophe.Luthy@hcuge.ch
Boris Cheval, 9, Chemin des Mines, 1202 Genève ; E-mail : boris.cheval@unige.ch

Hôpitaux Universitaires Genève

UNIVERSITÉ DE GENÈVE
SWISS CENTER FOR AFFECTIVE SCIENCES

Déclaration de consentement

Déclaration de consentement écrite pour la participation à un projet de recherche
Veuillez lire attentivement ce formulaire. N'hésitez pas à poser des questions lorsque vous ne comprenez pas quelque chose ou que vous souhaitez avoir des précisions.

**Numéro BASEC de l'étude:** (après soumission à la commission d'éthique compétente) :	
**Titre de l'étude :** (titre scientifique et titre usuel)	L'étude IMPACT. Promouvoir l'activité physique des patients en reprogrammant leurs réactions automatiques envers l'activité physique à l'aide de jeux sérieux sur ordinateur.
**Institution responsable :** (Promoteur avec adresse complète) :	Hôpitaux Universitaire de Genève / Université de Genève
**Lieu de réalisation de l'étude:**	Hôpital Beau-Séjour
**Médecin responsable du projet sur le site :** (nom et prénom en caractères d'imprimerie) :	LUTHY CHRISTOPHE
**Participant / participante :** (nom et prénom en caractères d'imprimerie) : Date de naissance :	☐ femme ☐ homme

- Je déclare avoir été informé, par le médecin-investigateur responsable de cette étude soussigné ou par un assistant de recherche, oralement et par écrit, des objectifs et du déroulement de l'étude ainsi que des effets présumés, des avantages, des inconvénients possibles et des risques éventuels.
- Je prends part à cette étude de façon volontaire et j'accepte le contenu de la feuille d'information qui m'a été remise sur l'étude précitée. J'ai eu suffisamment de temps pour prendre ma décision.
- J'ai reçu des réponses satisfaisantes aux questions que j'ai posées en relation avec ma participation à l'étude. Je conserve la feuille d'information et reçois une copie de ma déclaration de consentement écrite.
- J'accepte que mon médecin traitant soit informé de ma participation à l'étude.
- J'accepte que les spécialistes compétents du promoteur de l'étude, de la Commission d'éthique compétente puissent consulter mes données brutes afin de procéder à des contrôles, à condition toutefois que la confidentialité de ces données soit strictement assurée.
- Je serai informé des découvertes (fortuites) ayant une incidence directe sur ma santé. Si je ne souhaite pas obtenir ces informations, j'en aviserai le médecin-investigateur.
- Je sais que mes données personnelles peuvent être transmises à des fins de recherche dans le cadre de ce projet uniquement et sous une forme codée.
- Je peux, à tout moment et sans avoir à me justifier, révoquer mon consentement à participer à l'étude, sans que cela n'ait de répercussion défavorable sur la suite de ma prise en charge. Je sais que les données qui ont été recueillies jusque-là seront cependant analysées sauf dans le cas où j'indique ma volonté que les données ainsi que le formulaire de consentement soient totalement détruits.
- Je suis informé que la responsabilité civile de l'hôpital/institution couvre les dommages éventuels que je pourrais subir imputables au projet.

Cheval B, *et al. BMJ Open* 2021; 11:e053845. doi: 10.1136/bmjopen-2021-053845

Hôpitaux Universitaires Genève

UNIVERSITÉ DE GENÈVE
SWISS CENTER FOR AFFECTIVE SCIENCES

- Je suis conscient que les obligations mentionnées dans la feuille d'information destinée aux participants doivent être respectées pendant toute la durée de l'étude. La direction de l'étude peut m'en exclure à tout moment dans l'intérêt de ma santé.

Lieu, date	Signature du participant / de la participante

Attestation du médecin-investigateur : Par la présente, j'atteste avoir expliqué au participant / à la participante la nature, l'importance et la portée de l'étude. Je déclare satisfaire à toutes les obligations en relation avec ce projet conformément au droit en vigueur. Si je devais prendre connaissance, à quelque moment que ce soit durant la réalisation du projet, d'éléments susceptibles d'influer sur le consentement du participant / de la participante à prendre part au projet, je m'engage à l'en informer immédiatement.

Lieu, date	Nom et prénom du médecin-investigateur assurant l'information aux participants en caractères d'imprimerie.<br><br>Signature du médecin-investigateur

Cheval B, *et al. BMJ Open* 2021; 11:e053845. doi: 10.1136/bmjopen-2021-053845
