# Supplementary material for: Cognitive-bias modification intervention to improve physical activity in patients following a rehabilitation programme: protocol for the randomised controlled IMPACT trial
Source: BMJ Open. 2021 Sep 21;11(9):e053845. doi: 10.1136/bmjopen-2021-053845 (PMC8458354; doi:10.1136/bmjopen-2021-053845)
Supplement: Author's manuscript [file bmjopen-2021-053845.draft_revisions.pdf]

BMJ Open is committed to open peer review. As part of this commitment we make the peer review history of every article we publish publicly available.

When an article is published we post the peer reviewers' comments and the authors' responses online. We also post the versions of the paper that were used during peer review. These are the versions that the peer review comments apply to.

The versions of the paper that follow are the versions that were submitted during the peer review process. They are not the versions of record or the final published versions. They should not be cited or distributed as the published version of this manuscript.

BMJ Open is an open access journal and the full, final, typeset and author-corrected version of record of the manuscript is available on our site with no access controls, subscription charges or pay-per-view fees (http://bmjopen.bmj.com).

If you have any questions on BMJ Open's open peer review process please email info.bmjopen@bmj.com

BMJ Open

Improving physical activity (IMPACT) with the retraining of automatic approach tendencies: protocol of a randomized controlled clinical trial in patients following a rehabilitation program

Journal:	*BMJ Open*
Manuscript ID	bmjopen-2021-053845
Article Type:	Protocol
Date Submitted by the Author:	26-May-2021
Complete List of Authors:	Cheval, Boris; University of Geneva; University of Geneva, Department of Psychology<br>Finckh, Axel; Geneva University Hospitals, Department of Medicine<br>Maltagliati, Silvio; University Grenoble Alpes<br>Cullati, Stéphane ; University of Fribourg,<br>Sander, David; University of Geneva; University of Geneva, Department of Psychology<br>Friese, Malte; Saarland University<br>Wiers, Reinout W.; Univ Amsterdam<br>Boisgontier, Matthieu P.; Faculty of Health Sciences University of Ottawa; Bruyère Research Institute<br>Courvoisier, Delphine; Geneva University Hospitals, Division of clinical epidemiology<br>Luthy, C; Division of General Medical Rehabilitation, Geneva University Hospitals & University of Geneva
Keywords:	SPORTS MEDICINE, Quality in health care < HEALTH SERVICES ADMINISTRATION & MANAGEMENT, REHABILITATION MEDICINE

SCHOLARONE™
Manuscripts

I, the Submitting Author has the right to grant and does grant on behalf of all authors of the Work (as defined in the below author licence), an exclusive licence and/or a non-exclusive licence for contributions from authors who are: i) UK Crown employees; ii) where BMJ has agreed a CC-BY licence shall apply, and/or iii) in accordance with the terms applicable for US Federal Government officers or employees acting as part of their official duties; on a worldwide, perpetual, irrevocable, royalty-free basis to BMJ Publishing Group Ltd ("BMJ") its licensees and where the relevant Journal is co-owned by BMJ to the co-owners of the Journal, to publish the Work in this journal and any other BMJ products and to exploit all rights, as set out in our licence.

The Submitting Author accepts and understands that any supply made under these terms is made by BMJ to the Submitting Author unless you are acting as an employee on behalf of your employer or a postgraduate student of an affiliated institution which is paying any applicable article publishing charge ("APC") for Open Access articles. Where the Submitting Author wishes to make the Work available on an Open Access basis (and intends to pay the relevant APC), the terms of reuse of such Open Access shall be governed by a Creative Commons licence – details of these licences and which Creative Commons licence will apply to this Work are set out in our licence referred to above.

Other than as permitted in any relevant BMJ Author's Self Archiving Policies, I confirm this Work has not been accepted for publication elsewhere, is not being considered for publication elsewhere and does not duplicate material already published. I confirm all authors consent to publication of this Work and authorise the granting of this licence.

For peer review only

Improving physical activity (IMPACT) with the retraining of automatic approach tendencies: protocol of a randomized controlled clinical trial in patients following a rehabilitation program

Boris Cheval[1,2], Axel Finckh[3], Silvio Maltagliati[4], Stéphane Cullati[5,6], David Sander[1,2] Malte Friese[7], Reinout W. Wiers[8], Matthieu P. Boisgontier[9,10], Delphine Courvoisier[11], Christophe Luthy[12]

[1]Swiss Center for Affective Sciences, University of Geneva, Geneva, Switzerland

[2]Laboratory for the Study of Emotion Elicitation and Expression (E3Lab), Department of Psychology, University of Geneva, Geneva, Switzerland

[3]Division of Rheumatology, Department of Medicine, Geneva University Hospitals, Geneva, Switzerland

[4]Univ. Grenoble Alpes, SENS laboratory, Grenoble, France

[5]Population Health Laboratory, University of Fribourg, Switzerland.

[6]Department of Readaptation and Geriatrics, University of Geneva, Switzerland.

[7]Department of Psychology, Saarland University, Germany.

[8]Department of Psychology and center for Urban Mental Health, University of Amsterdam, The Netherlands.

[9]School of Rehabilitation Sciences, Faculty of Health Sciences, University of Ottawa, Ottawa, ON, Canada

[10]Bruyère Research Institute, Ottawa, ON, Canada

[11]Quality of Care Unit, Geneva University Hospitals, Geneva, Switzerland

[12]Division of General Medical Rehabilitation, University Hospitals of Geneva (HUG), Switzerland

For peer review only - http://bmjopen.bmj.com/site/about/guidelines.xhtml
For peer review only

Corresponding author: Campus, Biotech, Chemin des mines 9, 1202, Genève, Switzerland; boris.cheval@unige.ch; @ChevalBoris (B. Cheval).

Word count: 4800

Tables/Figures: 5

For peer review only

For peer review only - http://bmjopen.bmj.com/site/about/guidelines.xhtml

Abstract

Introduction. Being physically active is associated with a wide range of health benefits in patients. However, many patients do not engage in the recommended levels of physical activity (PA). To date, interventions promoting PA in patients mainly rely on providing knowledge about the benefits associated with PA to develop their motivation to be active. Yet, these interventions focusing on changing patients' conscious goals have proven to be rather ineffective in changing behaviors. Recent research on automatic factors (e.g., automatic approach tendencies) may provide additional targets for interventions. However, the implementation and evaluation of intervention designed to change these automatic bases of PA are rare. Consequently, little is known about whether and how interventions that target automatically activated processes toward PA can be effective in changing PA behaviors. The Improving physical activity (IMPACT) trial proposes to fill this knowledge gap by investigating the effect of a cognitive-bias modification intervention aiming to modify the automatic approach toward exercise-related stimuli on PA among patients.

Methods and analysis. The IMPACT trial is a single-center, placebo (sham controlled), double-blinded, phase 3 randomized controlled trial. Immediately after starting a rehabilitation program in the Division of General Medical Rehabilitation (University Hospital of Geneva; Switzerland), patients will be randomized (1:1 ratio) to receive either the cognitive-bias modification intervention consisting of a 12-session training program performed over 3 weeks and aiming to change automatic approach tendencies toward PA and sedentary behaviors, or a control intervention (placebo). Primary outcomes are the device-based PA collected during the intervention. Secondary outcomes are related to changes in (1) automatic approach tendencies and self-reported motivation to be active, (2) physical health and (3) mental health. Sedentary behaviors and self-reported PA will also be examined.

Ethics and dissemination. The study will be conducted in accordance with the Declaration of Helsinki. This trial was approved by the Ethics Committee of Geneva Canton, Switzerland (reference number: CCER2019-02257). Results will be published in relevant scientific journals and be disseminated in international conferences.

For peer review only

Strength and limitations of this study

- The IMPACT trial is the first randomized controlled trial investigating the additional beneficial effect of an easy deliverable cognitive-bias modification (CBM) intervention promoting physical activity among patients.

- The CBM intervention is anchored in the dual-process models of behaviors, arguing that automatic reactions toward physical activity represent additional targets for interventions.

- The findings from this well-powered study will provide evidence-based recommendations for clinical interventions aiming to promote physical activity.

For peer review only

Introduction

The health benefits of physical activity (PA) are well established and extensive. PA can reduce rates of cardiovascular diseases,[1] cancers,[2] hypertension,[3] diabetes,[4] obesity,[5] depression,[6] and all-cause mortality,[7] even more effectively than medication.[8] PA is safe and beneficial for almost everyone, while the risk of harm from moderate PA is small.[8 9] A recent systematic review and meta-analysis suggests that any PA, irrespective of the intensity, is beneficial for health.[7] In patients suffering from chronic diseases, increased PA is associated with reduced hospital admissions, decrease in pain, greater quality of life and mental health, and improvement in physical function.[8 10-13] These myriads of benefits even led the Academy of Medical Sciences to consider PA as a miracle cure.[14] Nevertheless, patients, similarly to the general population, remain largely physically inactive.[15-17]

Healthcare professionals are uniquely placed to promote PA among patients. Today, interventions aiming to enhance PA in patients largely relies on providing rational information about the benefits associated with PA. For example, a practical guide to help clinicians discussing about PA within a consultation has been recently proposed.[8] In this guide, clinicians are encouraged to rationally address patients' concerns about PA, to explain that there are more benefits to become active than to remain sedentary, to set an achievable goal, to identify barriers to be overcome, and finally to set a plan. This type of intervention guide is grounded in the dominant social-cognitive theories,[18] which contend that goals are proximal determinants of behaviors.[19 20] From these perspectives, changing patients' conscious goals should lead to substantial changes in their behaviors.[21 22] While these types interventions have proven to be effective to change PA behaviors to some extent,[23] meta-analyses also indicate that these approaches are more effective in changing intentions than in changing actual behavior.[24] Thus, developing additional interventions targeting alternative mechanisms is needed.

Recent research focusing on automatic mechanisms may provide additional targets for interventions.[25-28] For example, studies showed that in physically active individuals stimuli associated with PA attract attention,[29-32] trigger positive affective reactions,[33-36] and activate approach tendencies toward PA.[37-40] These automatically activated processes are thought to facilitate the translation of conscious goals into actual PA behaviors. Importantly, these automatic reactions predict PA behaviors above and beyond self-reported measures, such as the intention to be physically active[38], and are stronger predictors of spontaneous and unplanned actions that often consist of light-intensity physical activities.[41] As such, from this perspective,

For peer review only

physical inactivity is thought to also result from an imbalance between a strong motivation to be physically active, but weak automatic approach tendencies toward PA. Crucially, this imbalance between automatic and reflective processes may be particularly pronounced in patients, whose automatic reactions toward PA may be negatively biased by the fear, pain and discomfort felt during some exercises.[42] Thus, in comparison with the general population, patients may demonstrate more negative automatic reactions toward PA, including, for example, stronger negative affective reactions and weaker approach tendencies toward PA. One practical implication of these findings is that interventions designed to promote PA in patients might particularly benefit from directly targeting automatically activated processes toward PA.

What kinds of interventions can target automatically activated processes? New types of interventions have been developed to directly target these automatic reactions toward a given health behavior.[43 44] For example, in alcohol addiction, studies have used a cognitive-bias modification (CBM) intervention aimed at retraining automatic approach reactions toward alcohol using a computerized task.[45] In a CBM intervention, patients were repeatedly asked to push a joystick when exposed to alcohol-related pictures, simulating an avoidance movement. Specifically, in this computerized-based task, participants were asked to push or pull a joystick in response to the format of the pictures. For example, they were instructed to make a pushing movement when the picture presented on the screen was in the landscape format (i.e., avoidance), and to make a pulling movement when the picture was in the portrait format (i.e., approach). To ensure congruence with the participant's actions on the joystick, the picture became smaller when the participant pushed the joystick, and it became larger when the participant pulled the joystick. Participants received training in which they had to push the joystick away in response to pictures of alcohol (i.e., all alcohol pictures were presented in the push format) and to pull the joystick toward them in response to non-alcohol pictures (i.e., all non-alcohol pictures were presented in the pull format). Two large studies conducted in patients showed that adding a CBM intervention to a regular cognitive-behavior treatment yielded a beneficial effect on the relapse rates one year after treatment discharge, with a reduction of 9%,[46] 13%,[45] and 12%,[47] which could be attributed to changes in approach tendencies.[46 48] These interventions have also proven to be useful in impacting cigarette smoking,[49] social anxiety,[50] or eating behaviors.[51-53] Yet, it should be noted, the clinical effectiveness of CBM interventions has been criticized,[54 55] especially for anxiety and depression-related outcomes.[56-59]

For peer review only - http://bmjopen.bmj.com/site/about/guidelines.xhtml

To the best of our knowledge, however, only one study has been conducted to examine the effect of a brief CBM intervention on an exercise task in a sample of healthy young adults.[60] Specifically, using a manikin task,[41 61] a variant of the approach-avoidance joystick task, participants were explicitly trained to repeatedly approach a manikin toward pictures depicting PA and to avoid pictures depicting sedentary behaviors, by pressing keys on the keyboard. Results revealed that participants spent more time exercising during a laboratory exercise task of moderate intensity (i.e., doing squat), in comparison with control groups either trained to approach stimuli depicting sedentary behaviors and avoid stimuli depicting PA (i.e., reverse contingencies) or to approach and avoid stimuli depicting PA and sedentary behaviors equally often (sham controlled). These findings suggest that a single and brief CBM session targeting automatic approach tendencies toward PA and sedentary behaviors can have beneficial effect on laboratory-based PA behaviors. However, this study has at least two important limitations. First, it is unclear if and to what extent the PA behavior performed in the laboratory extends to behaviors performed in everyday life, thereby preventing the possibility to determine whether CMB manipulations can be effective in changing daily-life behaviors. Second, the study was conducted on a sample of rather physically active college students. As such the potential beneficial effect of adding a CBM intervention to a regular treatment in patients, a population which may particularly benefit from such manipulation, remains unknown.

Objectives

In sum, while recent research highlights the importance of targeting automatically activated processes related to PA, the effectiveness of interventions designed to change these presumed automatic bases of PA behaviors has been largely overlooked. Consequently, little is known about whether and how interventions that target automatically activated processes toward PA can be effective in changing behaviors. The primary objective of the IMPACT trial is to investigate the effectiveness of a CBM intervention targeting automatic approach tendencies toward exercise-related stimuli on PA patients in a rehabilitation program. This trial will be performed using a placebo, double-blinded, phase 3 randomized controlled trial. The secondary objectives are to evaluate the effect of this CBM intervention on changes in (1) automatic approach tendencies and self-reported motivation to be active, (2) physical health and (3) mental health. We hypothesize that the CBM intervention will be associated with higher levels of PA in patients during the rehabilitation program (H1). Moreover, we hypothesize that the CBM intervention will increase patients' automatic approach tendencies toward PA (H2a) and self-reported motivation to be active (H2b) but will decrease patients' automatic approach

For peer review only - http://bmjopen.bmj.com/site/about/guidelines.xhtml

tendencies toward sedentary behaviors (H2c). Finally, we predict that the CBM intervention will improve patients' physical and mental health (H3). All these hypotheses will be tested one week as well as one, three, six, and 12 months after the intervention.

Methods and data analysis

Study design

The IMPACT trial is a single-center, placebo (sham controlled), double-blinded, phase 3 randomized controlled trial. The trial will start (First-Participant-In) January 2022 in the ward 3DK of the Division of General Medical Rehabilitation (University Hospitals of Geneva; Switzerland) and will finish (Last-Participant-Out) in January 2024. Eligible patients will be randomly assigned to either the CBM intervention or the active control condition (placebo) in a 1:1 ratio. The current study follows the Standard Protocol Items: Recommendations for Interventional Trials (SPIRIT) statement.[62]

Eligibility criteria

The eligibility criteria are listed in Box 1. Participants fulfilling all the inclusion criteria are eligible for the study. The presence of the exclusion criterion will lead to the exclusion of the participant.

Box 1. Inclusion and exclusion criteria

Inclusion criteria

- Patients treated in the ward 3DK of the Division of General Medical Rehabilitation
- Aged 18 years or older
- Can comply with the study protocol
- Able to provide a written consent of participation in the trial.

Exclusion criteria

- Contraindication to PA in the view of the health status

Decision to include/exclude a participant

The decision to include/exclude a participant from this study will be jointly decided by the chief medical officer and the research assistant.

Participant screening, recruitment, and consent

For peer review only

All patients starting rehabilitation program in the ward 3DK of the Division of General Medical Rehabilitation, University Hospital of Geneva, Switzerland (from January 2022 to January 2024) will be approached during the first consultation with the chief medical officer and will receive an information sheet explaining the main objective of the IMPACT trial. The investigators will explain to each participant the nature of the study, its purpose, the procedures involved, the expected duration, the potential risks and benefits and any discomfort it may entail. Each participant will be informed that the participation in the study is voluntary and that he or she may withdraw from the study at any time and that withdrawal of consent will not affect his or her subsequent medical assistance and treatment. The participant will be informed that his or her medical records may be examined by authorized individuals other than their treating physician. All participants will be provided a participant information sheet and a consent form describing the study and providing sufficient information for participants to make an informed decision about their participation in the study. Participants will have time to carefully read the documents and can give their responses up to 24 hours after having received the documents. The formal consent of a participant, using the approved consent form, will be obtained before the participant is submitted to any study procedure. Participants will then complete a first questionnaire assessing the exclusion and inclusion criteria, as well as other screening measures. All the questionnaires will be assessed electronically using REDCap software. Finally, patients' expectations regarding the effects of the intervention will be assessed.[63] Table 1 provides an overview of all the baseline screening measures available. The study patient flow chart is provided in Figure 1.

Sample size

Based on estimates of the effect size of automatically activated processes,[53] a sample size calculation indicates that a minimum of 220 patients (110 per arm) would be needed to demonstrate efficacy, with a probability of committing a type I error < 5% and a probability of committing a type II error < 10%. We expect a loss to follow-up of 10 to 20% over one year. Thus, a total of 250 patients will be recruited.

Feasibility

The ward 3DK of the Division of General Medical Rehabilitation has 24 beds and treats on average 40 patients per month. We expect that 1 patient out of 5 will not agree (for various reasons) to participate in the study, thereby leading to a total of about 30 participants recruited per month. Consequently, we should be able to collect the target sample size in approximately

For peer review only - http://bmjopen.bmj.com/site/about/guidelines.xhtml

8-10 months. To accelerate and facilitate knowledge dissemination, all articles will be preprinted, and data and code shared on public repositories.

Interventions

All newly admitted patients will attend a meeting organized in the unit. The objective of this meeting will be to present and illustrate the health benefits of PA. Consistent with the recent practical guide to help healthcare professionals promoting PA to patients,[8] research assistants will follow the "Ask-Assess-Advise" structure for discussing PA behavior change in the consultation. Patients will also receive a watch tracking (i.e., polar) during the rehabilitation period and giving personalized feedback on their PA and sedentary behaviors. This procedure aims at increasing their self-reported motivation to be active, thereby allowing to examine the additional effects of the CBM intervention.

Intervention group: Training program of 15 sessions over 3 weeks (i.e., 5 sessions by week on average) using an adapted version of the Visual-Approach/Avoidance-by-the-Self Task (VAAST),[64] a task that have shown to produce large and replicable effects, compared with the manikin task. Specifically, patients will be asked to react to the format (i.e., portrait vs. landscape) of the pictures depicting PA and of sedentary behaviors by pressing twice the "move forward" or "move backward" key press to approach or avoid the pictures, respectively. Participants will be instructed to approach the picture when it appears in a portrait format, and to avoid it when the picture appears in a landscape format (the rule will be counterbalanced between participants). Of note, unlike the previous study that relied on an explicit instruction task (i.e., participants were asked to respond to the content of the pictures),[60] the current study uses an irrelevant feature task (i.e., participants were asked to respond to the format of the pictures). This irrelevant feature task allows a training without explicit instruction. Congruent with the patient's approach or avoidance response, the whole visual environment will zoom in on the picture to simulate an approach movement and zoom out to simulate an avoidance movement. A change by 10% after each key press will be used to give the impression to walk forward or backward as a consequence of the responses. Participants in the intervention group will receive training in which all pictures depicting PA will be presented in the approach format, and all pictures depicting sedentary behaviors will be presented in the avoidance format. Each training session will consist of 144 trials for a total duration of approximately 10 minutes. At the first session and at the beginning of each week, the training session will be preceded by 96 assessment trials in which the contingency of approaching or avoiding PA or sedentary

For peer review only - http://bmjopen.bmj.com/site/about/guidelines.xhtml

behaviors will be 50%. Assessment trials will allow to measure patients' automatic approach-avoidance tendencies toward PA and sedentary behaviors (see Figure 2).

Comparator group: Patients in the comparator group (placebo; sham controlled) will not be trained to approach PA and to avoid sedentary behaviors. Specifically, the retraining sessions will also consist of 144 trials, but the task will require an equal number of approach and avoidance responses to both stimuli depicting PA and sedentary behaviors (see Figure 2).

Stimuli: Stimuli representing PA and sedentary behaviors will be created using the Unity software ®. A set of 195 pictures including 14 avatars (50% women) in either active (walking and running) and inactive posture (sit on a cubicle) will be tested in a pilot study to identify the 48 pictures the most associated with "movement and physically active behaviors" and the 48 pictures the most associated with "rest and physically inactive behaviors" using two visual analogic scales (VAS 1; *"please indicate how this image is, in your opinion, associated with a behavior that requires: 0 = no physical exertion at all, 100 = a lot of physical exertion*"; VAS 2; *"Please indicate how closely this image is associated with: 0 = resting, sedentary behavior, 100 = movement, very active behavior").* The credibility of the pictures will also be tested ("*how realistic do you think this person's behavior is? Realistic meaning that the images may resemble to a real-life behavior*"; on a VAS from 0 = behavior not at all realistic; 100 = behavior very realistic) and for agreeableness *("how pleasant/sympathetic do you find the person in this image? For example, would you like to talk to her/him"*; from 0 = very unpleasant/antipathetic, 100 = very pleasant/sympathetic). Pictures will be built to match for color, brightness, and visual complexity. To examine the generalization of training effects,[65] in both the intervention and comparator group, only half of the pictures used in the assessment phase will be included on the training phase (the selected pictures will be counterbalanced across participants).

<u>Randomization and blinding</u>

The research assistants and the participants will be blinded to the allocation of the groups. At the end of the trial, the success of the participant blinding will be examined by asking the participants to guess in what group there were, including a percentage of certainty. Moreover, the success of research assistants blinding will be examined by asking each research assistant if they were able to detect the group (comparator vs. intervention) when they conducted the data collection.

For peer review only - http://bmjopen.bmj.com/site/about/guidelines.xhtml

The randomization will be generated on a computer and will be performed using permuted blocks (size = 8). To ensure that the research team will be blinded to the randomization, an independent co-worker will carry out the randomization. The patient's identification number will be used to determine the sequence of randomization. Patients will be randomized in a 1:1 ratio between the intervention and active control condition.

Outcomes

Primary outcomes

The primary outcome will be the accelerometer-based time spent in PA. Following recommendations in patients,[66] a three-axis accelerometer (Actigraph GT3X+; Pensacola, USA) will be used to assess PA. Patients will be given the accelerometer and related indications during the first training session. They will be asked to wear the accelerometer for the full week and to return during the next appointment. One-minute epochs will be used for data analyses and non-wear time will be defined as ≥ 59 consecutive minutes of zero counts. Daily data will be included if the wear time is ≥ 10 waking hours per day [67]. Data will be included if ≥ 4 days met the aforementioned conditions.[68] The times spent in light, moderate, and vigorous PA will be determined through previously validated cut points,[69] in bouts lasting at least 10 min, and will be used as an outcome. Then, in the week following the rehabilitation period, participants will be asked to wear the accelerometer for one week. Finally, participants will be asked to wear the accelerometer for one week at one, three, six, and 12 months post-intervention.

Secondary outcomes

The secondary outcomes will be the changes in (1) automatic approach tendencies and self-reported motivation to be active, (2) physical health, and (3) mental health. Sedentary behaviors and self-reported PA will also be examined. Table 2 provides an overview of all the outcomes measures and Table 3 provides the schedule of assessment.

Data analysis

Statistical analyses will be performed according to the intention-to-treat (ITT) principle and will abide by the Consolidated Standards of Reporting Trials (CONSORT) guidelines. Analysis will be conducted in a blinded way. We will use mean, standard deviation (SD), median, and range values to summarize the continuous data. The primary outcome will be analyzed using

For peer review only - http://bmjopen.bmj.com/site/about/guidelines.xhtml

mixed effects models, which account for the nested structure of the data (i.e., multiple observations within a single participant), thereby providing accurate parameter estimates with acceptable Type I error rates.[70] To formally examine the effect of the intervention on the evolution of PA within the rehabilitation period, models will include interaction terms between conditions (intervention group vs. comparator group) and number of days within the rehabilitation program (linear and quadratic). The number of days should be relatively equal between patients (about 21 days) but may differ to some extent (some patients can leave earlier or other later than 21 days). A statistically significant interaction will indicate that the rate of PA change throughout the rehabilitation program would be different across the conditions. The quadratic effect of the number of days will be included to account for potential non-linear change of PA across the rehabilitation period. This will allow, for instance, to model the possibility that the effect of the intervention will take some sessions before becoming effective or that no additional effect could be hoped after a certain number of sessions. The continuous secondary outcomes will be treated in the similar way to the primary outcome. Moreover, moderator analyses (i.e., for motivation to change, usual level of PA, personality, expectations for improvement) will be conducted. All analyses will be conducted using R software®. Any deviation from the original statistical plan will be described and justified in the final trial report.

<u>Data security, management, and monitoring:</u>

Project data will be handled with uttermost discretion and will be only accessible to authorized personnel who require the data to fulfill their duties within the scope of the research project. On the online Case Report Forms (CRFs) and other specific documents, participants are only identified by a unique participant number. The online CRF will be created using Redcap.

Data recording: The dataset will be accompanied by a README file, which will describe the directory hierarchy and file naming convention. The directory will contain an INFO file describing the experimental protocol used in that experiment. This INFO file will also record any deviation from the protocol and other useful contextual information. This procedure should allow the data to be easily understood by other researchers and should support future reuse of the data. Metadata will be created to provide contextual information required to interpret data. This metadata file will be created in accordance with the Data Documentation Initiative (DDI). In particular, the metadata file will include short unique identifier, the name of the author(s), the content, the date of creation, the locations, the reason why the data was generated, and how the data was created. The codebook will explicitly indicate the name, explanations, and the

For peer review only - http://bmjopen.bmj.com/site/about/guidelines.xhtml
For peer review only

modalities of the different variables measured in the experiment. In addition, it will include information on the study design and contain all information necessary for another analyst to use the data accurately.

Data anonymization: Individual participant information collected during the study is considered confidential and disclosure to third parties is prohibited. Subject confidentiality will be ensured by utilizing subject identification code numbers to correspond to treatment data in the computer files. Only a minority of personnel (i.e., the principal investigator and chief medical officer) will have access to the data in a non-coded form.

Data storage: Participant data on a secure database in accordance with the General Data Protection Regulations (2018). Three copies of the data will be stored. First, original data will be stored on the principal investigator's computer, which will be backed up daily, and protected by a password. Additionally, data will be stored on a secure server hosted by the University of Geneva. Finally, data will be stored on an external device at a different location and be protected by a password. The original notebook will be stored in the principal investigator's laboratory. Local version of the data for statistical analysis will remain on a University computer, and be password protected. Each person who collected the data will have the responsibility to annotate their data within the metadata. Nevertheless, the principal investigator will have the responsibility to weekly check that the data is properly processed, documented, and stored. All study data will be archived for a minimum of 10 years beyond the end of the randomized controlled trial.

Trial monitoring: The PI will organize a proper training of all involved study personnel to ensure that the study will be conducted according to the protocol. Research assistants should understand the detailed contents of the protocol before starting the data collection. For quality assurance the Ethics Committee may visit the research sites. Direct access to the source data and all project-related files and documents must be granted on such occasions.

Patient and public involvement in the trial design

No patient or public was involved in the present study.

Ethics and dissemination

For peer review only - http://bmjopen.bmj.com/site/about/guidelines.xhtml

The study was approved by the Ethics Committee of Geneva Canton, Switzerland (reference number: CCER2019-02257). All participants will give an informed consent to participate in the study.

Results will be published in relevant scientific journals and be disseminated in international conferences. Anonymity of the participants will be guaranteed when presenting the data at scientific meetings or publishing them in scientific journals. Individual participant information collected during the study is considered confidential and disclosure to third parties is prohibited.

Data sharing and reuse: Datasets and metadata from this trial will be deposited in ZENODO (a generic and free repository based at CERN, Geneva), and made public at the time of publication. Data in the repository will be stored in accordance with funder and university data policies. Particularly, original datasets, original software script and code, and original raw data will be deposited. However, as stressed above, personal data will be anonymized before diffusion.

Discussion

PA is associated with a wide range of health benefits,[1-7] but patients, similarly to the general population, remain largely physically inactive. Promoting PA to patients is thus urgently warranted, and healthcare professionals are uniquely placed to do so.[8] To date, interventions mainly rely on providing rational information to change patients' conscious goals and motivation to be active. Yet, these approaches are insufficient to substantially impact actual behaviors.[24] One explanation for this lack of effectiveness draws on recent observations suggesting that automatic reactions toward exercise-related stimuli are involved in the regulation of PA.[32 33 38 71 72] As such, developing interventions targeting both reflective (e.g., motivation) and automatic (e.g., approach tendencies) precursors of PA could be particularly effective. This protocol paper outlines the design of the IMPACT trial, the first placebo, double-blinded, randomized controlled trial examining the effectiveness of a CBM intervention targeting automatic approach tendencies toward exercise-related stimuli on PA in patients in rehabilitation program remains. The IMPACT trial will focus on an accelerometer-based measure of PA as the primary outcome due to all the extensive benefits associated with being physically active. The secondary outcomes will allow examining other positive-side effects of the intervention on physical and mental health.

For peer review only - http://bmjopen.bmj.com/site/about/guidelines.xhtml

For peer review only

In conclusion, PA is a key factor to improve the management of patients' diseases. Helping patients to become more active is likely to promote their recovery, their physical and mental health, as well as to reduce the development of other comorbidities. Targeting automatic reactions toward PA, which may be negatively biased in patients, is particularly innovative. Furthermore, this low cost and easily deliverable intervention could be rapidly implemented on a large scale to help patients become more physically active. The findings from this study will provide evidence-based conclusions for future interventions promoting PA in patients.

For peer review only

For peer review only - http://bmjopen.bmj.com/site/about/guidelines.xhtml

Contributors:

B.C.: conceptualization, writing – original draft; A.F.: conceptualization, writing – review & editing; S.M.: Methodology – creation of the tasks, writing – review & editing; S.C.: writing – review & editing; D.S.: writing – review & editing; M.S.: writing – review & editing; R.W.W.: writing – review & editing; M.P.B.: conceptualization, writing – original draft; R.W.: writing – review & editing; D.C.: supervision, resources – provision on instrumentation, writing – review & editing; C.L.: supervision, resources – provision of study materials, writing – original draft

Funding: The current project is related to the clinical part of a project funded by the Swiss National Science Foundation (699'915 CHF). Project Ambizione n° PZ00P1_180040. The purchase of protocol-related material (i.e., 30 Polar watches and accelerometers) is provided by the non-operating fund of the Division of General Medical Rehabilitation, University Hospital of Geneva, Switzerland. The computer equipment (3 laptops) will be made available by Division of General Medical Rehabilitation, University Hospital of Geneva. M.P.B. is supported by the Natural Sciences and Engineering Research Council of Canada (RGPIN-2021-03153).

Competing interests: None declared

Participants consent: Not obtained

Ethics approval: Ethics Committee of the Geneva University Hospitals, Switzerland

Provenance and peer review: Not commissioned; peer reviewed for ethical and funding approval prior to submission.

Data availability statement: Datasets and metadata from this trial will be deposited in ZENODO (a generic and free repository based at CERN, Geneva) for the duration of 20 years, and made public at the time of publication.

For peer review only

References:

1. Wahid A, Manek N, Nichols M, et al. Quantifying the association between physical activity and cardiovascular disease and diabetes: a systematic review and meta-analysis. *J Am Heart Assoc* 2016;5:e002495.
2. Moore SC, Lee I-M, Weiderpass E, et al. Association of leisure-time physical activity with risk of 26 types of cancer in 1.44 million adults. *JAMA Intern Med* 2016;176:816-25.
3. Liu X, Zhang D, Liu Y, et al. Dose–response association between physical activity and incident hypertension: a systematic review and meta-analysis of cohort studies. *Hypertension* 2017;69:813-20.
4. Aune D, Norat T, Leitzmann M, et al. Physical activity and the risk of type 2 diabetes: a systematic review and dose–response meta-analysis. *Eur J Epidemiol* 2015;30:529-42.
5. Bleich SN, Vercammen KA, Zatz LY, et al. Interventions to prevent global childhood overweight and obesity: a systematic review. *Lancet Diabetes Endocrinol* 2018;6:332-46.
6. Schuch F, Vancampfort D, Firth J, et al. Physical activity and sedentary behavior in people with major depressive disorder: a systematic review and meta-analysis. *J Affect Disord* 2017;210:139-50.
7. Ekelund U, Tarp J, Steene-Johannessen J, et al. Dose-response associations between accelerometry measured physical activity and sedentary time and all cause mortality: systematic review and harmonised meta-analysis. *BMJ* 2019;366:l4570.
8. Haseler C, Crooke R, Haseler T. Promoting physical activity to patients. *BMJ* 2019;366:l5230.
9. Godlee F. The miracle cure. *BMJ* 2019;366:l5605.
10. Anderson L, Oldridge N, Thompson DR, et al. Exercise-based cardiac rehabilitation for coronary heart disease: Cochrane systematic review and meta-analysis. *J Am Coll Cardiol* 2016;67:1-12.
11. Stevinson C, Lawlor DA, Fox KR. Exercise interventions for cancer patients: systematic review of controlled trials. *Cancer causes & control* 2004;15:1035-56.
12. Taylor RS, Brown A, Ebrahim S, et al. Exercise-based rehabilitation for patients with coronary heart disease: systematic review and meta-analysis of randomized controlled trials. *The American journal of medicine* 2004;116:682-92.
13. Zanuso S, Jimenez A, Pugliese G, et al. Exercise for the management of type 2 diabetes: a review of the evidence. *Acta Diabetol* 2010;47:15-22.
14. Exercise: the miracle cure and the role of the doctor in promoting it. https://www.aomrc.org.uk/wp-content/uploads/2016/05/Exercise_the_Miracle_Cure_0215.pdf. 2015
15. Johansen KL, Chertow GM, Ng AV, et al. Physical activity levels in patients on hemodialysis and healthy sedentary controls. *Kidney Int* 2000;57:2564-70.
16. Watz H, Waschki B, Meyer T, et al. Physical activity in patients with COPD. *Eur Respir J* 2009;33:262-72.
17. Jeong S-W, Kim S-H, Kang S-H, et al. Mortality reduction with physical activity in patients with and without cardiovascular disease. *Eur Heart J* 2019;40:3547-55.
18. Rhodes RE, Nigg CR. Advancing physical activity theory: a review and future directions. *Exerc Sport Sci Rev* 2011;39:113-19.
19. Bandura A. Self-efficacy: The exercise of control (1st edition). New York: Worth Publishers 1997.
20. Carver CS, Scheier MF. On the self-regulation of behavior. New York: Cambridge University Press 1998.

For peer review only - http://bmjopen.bmj.com/site/about/guidelines.xhtml

21. Sheeran P, Gollwitzer PM, Bargh JA. Nonconscious processes and health. *Health Psychol* 2013;32:460-73.
22. Brand R, Cheval B. Theories to explain exercise motivation and physical inactivity: Ways of expanding our current theoretical perspective. *Front Psychol* 2019;10:1147.
23. Hillsdon M, Foster C, Thorogood M. Interventions for promoting physical activity. *Cochrane Database Syst Rev* 2005;25:CD003180.
24. Rhodes RE, Dickau L. Experimental evidence for the intention–behavior relationship in the physical activity domain: a meta-analysis. *Health Psychol* 2012;31:724-27.
25. Cheval B, Radel R, Neva JL, et al. Behavioral and neural evidence of the rewarding value of exercise behaviors: a systematic review. *Sports Med* 2018;48:1389-404.
26. Brand R, Ekkekakis P. Affective–Reflective Theory of physical inactivity and exercise. *Ger J Exerc Sport Res* 2018;48:48-58.
27. Conroy DE, Berry TR. Automatic affective evaluations of physical activity. *Exerc Sport Sci Rev* 2017;45:230-37.
28. Cheval B, Sarrazin P, Boisgontier MP, et al. Temptations toward behaviors minimizing energetic costs (BMEC) automatically activate physical activity goals in successful exercisers. *Psychol Sport Exerc* 2017;30:110-17.
29. Berry TR. Who's even interested in the exercise message? Attentional bias for exercise and sedentary-lifestyle related words. *J Sport Exerc Psychol* 2006;28:4-17.
30. Berry TR, Spence JC, Stolp SM. Attentional bias for exercise-related images. *Res Q Exerc Sport* 2011;82:302-9.
31. Calitri R, Lowe R, Eves FF, et al. Associations between visual attention, implicit and explicit attitude and behaviour for physical activity. *Psychol Health* 2009;24:1105-23.
32. Cheval B, Miller MW, Orsholits D, et al. Physically active individuals look for more: an eye-tracking study of attentional bias. *Psychophysiology* 2020;57:e13582.
33. Conroy DE, Hyde AL, Doerksen SE, et al. Implicit attitudes and explicit motivation prospectively predict physical activity. *Ann Behav Med* 2010;39:112-18.
34. Rebar AL, Ram N, Conroy DE. Using the EZ-diffusion model to score a single-category implicit association test of physical activity. *Psychol Sport Exerc* 2015;16:96-105.
35. Bluemke M, Brand R, Schweizer G, et al. Exercise might be good for me, but I don't feel good about it: do automatic associations predict exercise behavior? *J Sport Exerc Psychol* 2010;32:137-53.
36. Chevance G, Caudroit J, Romain AJ, et al. The adoption of physical activity and eating behaviors among persons with obesity and in the general population: the role of implicit attitudes within the Theory of Planned Behavior. *Psychol Health Med* 2017;22:319-24.
37. Hannan TE, Moffitt RL, Neumann DL, et al. Implicit approach–avoidance associations predict leisure-time exercise independently of explicit exercise motivation. *Sport Exerc Perform Psychol* 2019;8:210-22.
38. Cheval B, Sarrazin P, Isoard-Gautheur S, et al. Reflective and impulsive processes explain (in)effectiveness of messages promoting physical activity: a randomized controlled trial. *Health Psychol* 2015;34:10-19.
39. Cheval B, Sarrazin P, Isoard-Gautheur S, et al. How impulsivity shapes the interplay of impulsive and reflective processes involved in objective physical activity. *Pers Individ Dif* 2016;96:132-7.
40. Cheval B, Tipura E, Burra N, et al. Avoiding sedentary behaviors requires more cortical resources than avoiding physical activity: an EEG study. *Neuropsychologia* 2018;119:68-80.
41. Cheval B, Sarrazin P, Pelletier L. Impulsive approach tendencies towards physical activity and sedentary behaviors, but not reflective intentions, prospectively predict non-exercise activity thermogenesis. *Plos One* 2014;9:e115238.

For peer review only - http://bmjopen.bmj.com/site/about/guidelines.xhtml

42. Shelby RA, Somers TJ, Keefe FJ, et al. Domain specific self-efficacy mediates the impact of pain catastrophizing on pain and disability in overweight and obese osteoarthritis patients. *J Pain* 2008;9:912-19.
43. Friese M, Hofmann W, Wiers RW. On taming horses and strengthening riders: Recent developments in research on interventions to improve self-control in health behaviors. *Self Identity* 2011;10:336-51.
44. Marteau TM, Hollands GJ, Fletcher PC. Changing human behavior to prevent disease: the importance of targeting automatic processes. *Science* 2012;337:1492-95.
45. Wiers RW, Eberl C, Rinck M, et al. Retraining automatic action tendencies changes alcoholic patients' approach bias for alcohol and improves treatment outcome. *Psychol Sci* 2011;22:490-97.
46. Eberl C, Wiers RW, Pawelczack S, et al. Approach bias modification in alcohol dependence: do clinical effects replicate and for whom does it work best? *Dev Cogn Neurosci* 2013;4:38-51.
47. Manning V, Garfield JB, Staiger PK, et al. Effect of cognitive bias modification on early relapse among adults undergoing inpatient alcohol withdrawal treatment: a randomized clinical trial. *JAMA Psychiat* 2021;78:133-40.
48. Gladwin TE, Rinck M, Eberl C, et al. Mediation of cognitive bias modification for alcohol addiction via stimulus-specific alcohol avoidance association. *Alcohol Clin Exp Res* 2015;39:101-07.
49. Wittekind CE, Feist A, Schneider BC, et al. The approach-avoidance task as an online intervention in cigarette smoking: A pilot study. *J Behav Ther Exp* 2015;46:115-20.
50. Taylor CT, Amir N. Modifying automatic approach action tendencies in individuals with elevated social anxiety symptoms. *Behaviour research and therapy* 2012;50:529-36.
51. Fishbach A, Friedman RS, Kruglanski AW. Leading us not unto temptation: Momentary allurements elicit overriding goal activation. *J Pers Soc Psychol* 2003;84:296-309. doi: 10.1037/0022-3514.84.2.296
52. Fishbach A, Shah JY. Self-control in action: implicit dispositions toward goals and away from temptations. *J Pers Soc Psychol* 2006;90:820.
53. Aulbach MB, Knittle K, Haukkala A. Implicit process interventions in eating behaviour: a meta-analysis examining mediators and moderators. *Health Psychol Rev* 2019;13:179-208.
54. Field M, Duka T, Eastwood B, et al. Experimental manipulation of attentional biases in heavy drinkers: do the effects generalise? *Psychopharmacology* 2007;192:593-608.
55. Schoenmakers T, Wiers RW, Jones BT, et al. Attentional re-training decreases attentional bias in heavy drinkers without generalization. *Addiction* 2007;102:399-405.
56. Cristea IA, Kok RN, Cuijpers P. The effectiveness of cognitive bias modification interventions for substance addictions: a meta-analysis. *Plos One* 2016;11:e0162226.
57. Cristea IA, Kok RN, Cuijpers P. Efficacy of cognitive bias modification interventions in anxiety and depression: meta-analysis. *Br J Psychiatry* 2015;206:7-16.
58. Mogoaşe C, David D, Koster EH. Clinical efficacy of attentional bias modification procedures: An updated meta-analysis. *Journal of Clinical Psychology* 2014;70:1133-57.
59. Boffo M, Zerhouni O, Gronau QF, et al. Cognitive bias modification for behavior change in alcohol and smoking addiction: Bayesian meta-analysis of individual participant data. *Neuropsychol Rev* 2019;29:52-78.
60. Cheval B, Sarrazin P, Pelletier L, et al. Effect of retraining approach-avoidance tendencies on an exercise task: A randomized controlled trial. *J Phys Act Health* 2016;13:1396-403.

For peer review only - http://bmjopen.bmj.com/site/about/guidelines.xhtml

61. Krieglmeyer R, Deutsch R. Comparing measures of approach–avoidance behaviour: The manikin task vs. two versions of the joystick task. *Cogn Emot* 2010;24:810-28.
62. Chan A-W, Tetzlaff JM, Altman DG, et al. SPIRIT 2013 statement: defining standard protocol items for clinical trials. *Ann Intern Med* 2013;158:200-07.
63. Boot WR, Simons DJ, Stothart C, et al. The pervasive problem with placebos in psychology: Why active control groups are not sufficient to rule out placebo effects. *Perspect Psychol Sci* 2013;8:445-54.
64. Rougier M, Muller D, Ric F, et al. A new look at sensorimotor aspects in approach/avoidance tendencies: The role of visual whole-body movement information. *J Exp Soc Psychol* 2018;76:42-53.
65. Wiers RW, Rinck M, Kordts R, et al. Retraining automatic action-tendencies to approach alcohol in hazardous drinkers. *Addiction* 2010;105:279-87.
66. Mantri S, Wood S, Duda JE, et al. Comparing self-reported and objective monitoring of physical activity in Parkinson disease. *Parkinsonism Relat Disord* 2019;67:56-59.
67. Evenson KR, Terry JW. Assessment of differing definitions of accelerometer nonwear time. *Res Q Exerc Sport* 2009;80:355-62.
68. Matthews CE, Ainsworth BE, Thompson RW, et al. Sources of variance in daily physical activity levels as measured by an accelerometer. *Med Sci Sports Exerc* 2002;34:1376-81.
69. Freedson PS, Melanson E, Sirard J. Calibration of the computer science and applications, inc. accelerometer. *Med Sci Sports Exerc* 1998;30:777-81.
70. Boisgontier MP, Cheval B. The anova to mixed model transition. *Neurosci Biobehav Rev* 2016;68:1004-05.
71. Cheval B, Cabral DAR, Daou M, et al. Inhibitory control elicited by physical activity and inactivity stimuli: an EEG study. *Motiv Sci* 2021
72. Cheval B, Daou M, Cabral DAR, et al. Higher inhibitory control is required to escape the innate attraction to effort minimization. *Psychol Sport Exerc* 2020;51:101781.
73. Grimby G, Börjesson M, Jonsdottir I, et al. The "Saltin–Grimby physical activity level scale" and its application to health research. *Scand J Med Sci Spor* 2015;25:119-25.
74. Gosling SD, Rentfrow PJ, Swann Jr WB. A very brief measure of the Big-Five personality domains. *J Res Pers* 2003;37:504-28.
75. Lowe R, Eves F, Carroll D. The influence of affective and instrumental beliefs on exercise intentions and behavior: A longitudinal analysis. *J Appl Soc Psychol* 2002;32:1241-52.
76. French DP, Sutton S, Hennings SJ, et al. The Importance of Affective Beliefs and Attitudes in the Theory of Planned Behavior: Predicting Intention to Increase Physical Activity 1. *J Appl Soc Psychol* 2005;35:1824-48.
77. Leong DP, Teo KK, Rangarajan S, et al. Prognostic value of grip strength: findings from the Prospective Urban Rural Epidemiology (PURE) study. *Lancet* 2015;386:266-73.
78. Mang CS, McEwen LM, MacIsaac JL, et al. Exploring genetic influences underlying acute aerobic exercise effects on motor learning. *Sci Rep* 2017;7:12123.
79. Craig CL, Marshall AL, Sjostrom M, et al. International physical activity questionnaire: 12-country reliability and validity. *Med Sci Sports Exerc* 2003;35:1381-95.

For peer review only

Table 1. Overview of the baseline screening measures

**Measures**	**Assessment method**
**Inclusion criteria**	
Patients treated in ward 3DK of the Division of General Medical Rehabilitation	During the first meeting with the research assistant.
≥ 18 years of age	
Can comply with study protocol	
Able to provide a written consent	
**Exclusion criterion**	
Contraindication to PA in the view of the health status	During the first meeting with the research assistant.
**Additional baseline screening assessment**	
Medical evaluation (questionnaires and objective tests)	Patients' diseases and treatment characteristics (medical burden, comorbidity, body mass index, mobility test, functional independence, health-related quality of life).
Sociodemographic characteristics	Questionnaires (age, sex, height, weight).
Usual level of PA	Saltin-Grimby PA Level Scale (SGPALS).[73]
Personality	Ten-Item Personality Inventory (TIPI).[74]
Expectations for improvement	A questionnaire measuring patients' thoughts about the effects of the intervention.[63]
Self-reported motivation to change	Questionnaire measuring patients' motivation to change their condition, to avoid a new treatment, and to engage in more PA in the future.
Self-reported ability to implement daily-life PA	Questionnaire measuring patients' self-reported ability to adopt regular PA in their daily life.

For peer review only

Table 2. Outcomes measures of the IMPACT trial and assessment time point

**Outcome**	**Assessment method**
**Primary outcomes**	
PA and sedentary behaviors	Accelerometer-based PA (Actigraph GT3X+) to measure the time spent in light, moderate, and vigorous PA.
**Secondary outcomes**	
Reflective and automatic precursors of PA	
Attitudes	Instrumental (i.e., useful, beneficial) and affective (i.e., enjoyable, interesting) attitudes toward PA using a short, self-reported questionnaire.[75 76]
Approach tendencies	The Visual-Approach/Avoidance-by-the-Self Task (VAAST).[64] A computerized reaction-time task assessing automatic approach tendencies toward PA and sedentary behaviors
Physical Health	
Weight	Weight (accuracy 0.1 kg) was assessed with participants clothed (lightweight clothing)
Muscle strength	Grip strength measured with a handheld dynamometer.[77]
Cardiorespiratory fitness	Maximal graded exercise test. [78]
Perceived global physical health	Global physical health Patient-Reported Outcomes Measurement Information System (PROMIS)scale.
Mental health	
Perceived physical functioning	
Perceived pain interference	Pain interference and pain intensity PROMIS scales.
Depression, anxiety, general life satisfaction	Anxiety, depression, general life satisfaction PROMIS scales.
Self-efficacy	Self-efficacy for managing chronic conditions PROMIS scales.
Social role	Ability to participants in social roles and activities PROMIS scale.
Other PA-related measures	
Self-reported behaviors	The International PA Questionnaire to measure the time spent in PA and in sedentary behaviors.[79]
Sedentary behaviors	Accelerometer-based sedentary behaviors (Actigraph GT3X+)

For peer review only

Table 3. Schedule of assessment

WEEK 1	-1 day	0	+1	+2	+3	+4
**Visit**	Information	Screening	1st training session	2nd training session	3rd training session	4th training session
**Oral and written patient information**	+					
**Informed written consent**		+				
**Inclusion exclusion criteria**		+				
**Additional baseline screening assessment**		+				
**Self-reported PA (usual week)**		+				
**Intervention**			+	+	+	+
**Motivation to be active**			+			
**Approach tendencies**			+			
**Physical health**			+			
**Mental health**			+			
**Accelerometer-based PA**			Continuously across the week			

WEEK 2	+1	+2	+3	+4
**Visit**	1st training session	2nd training session	3rd training session	4th training session
**Intervention**	+	+	+	+
**Motivation to be active**	+			
**Approach tendencies**	+			
**Physical health**	+			
**Mental health**	+			
**Accelerometer-based PA**	Continuously across the week			

WEEK 3	+1	+2	+3	+4
**Visit**	1st training session	2nd training session	3rd training session	4th training session
**Intervention**	+	+	+	+
**Motivation to be active**	+			
**Approach tendencies**	+			
**Physical health**	+			
**Mental health**	+			
**Accelerometer-based PA**	Continuously across the week			

Post-intervention	1 week	1 month	3 months	6 months	12 months
**Motivation to be active**	+	+	+	+	+
**Approach tendencies**	+	+	+	+	+
**Physical health**	+	+	+	+	+
**Mental health**	+	+	+	+	+

For peer review only - http://bmjopen.bmj.com/site/about/guidelines.xhtml

**Self-reported and accelerometer-based PA (during one week)**	+	+	+	+	+

For peer review only

Figure 1. Flow chart

Figure 2. Study design and of Cognitive-Bias Modification (CBM) task

Note. A. Study design. B. Illustration of the Cognitive-Bias Modification (CBM) task. In the CBM task, participants are asked to approach or avoid the picture appearing on the screen depending on its format (i.e., portrait vs. landscape, counterbalanced across participants). Participants are asked to approach the picture in the approach conditions and to avoid the picture in the avoidance conditions. In the intervention condition, all pictures depicting physical activity are presented in the approach format, and all the pictures depicting sedentary behaviors are presented in the avoidance format. In the control condition, the pictures depicting physical activity and sedentary behaviors are equally distributed across formats (i.e., 50%-50%).

For peer review only

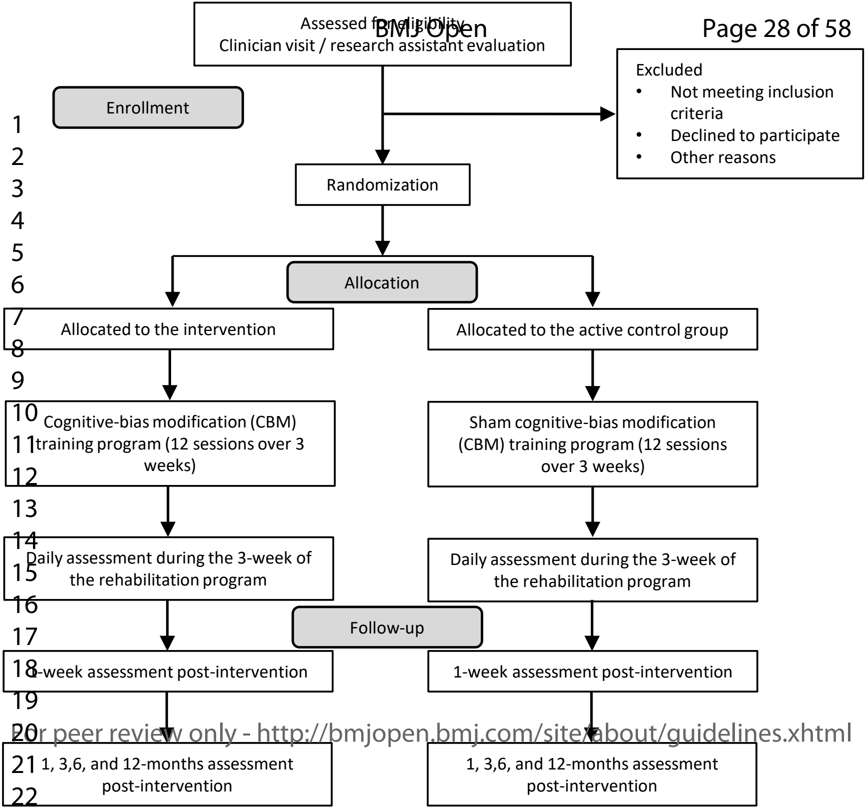

For peer review only - http://bmjopen.bmj.com/site/about/guidelines.xhtml

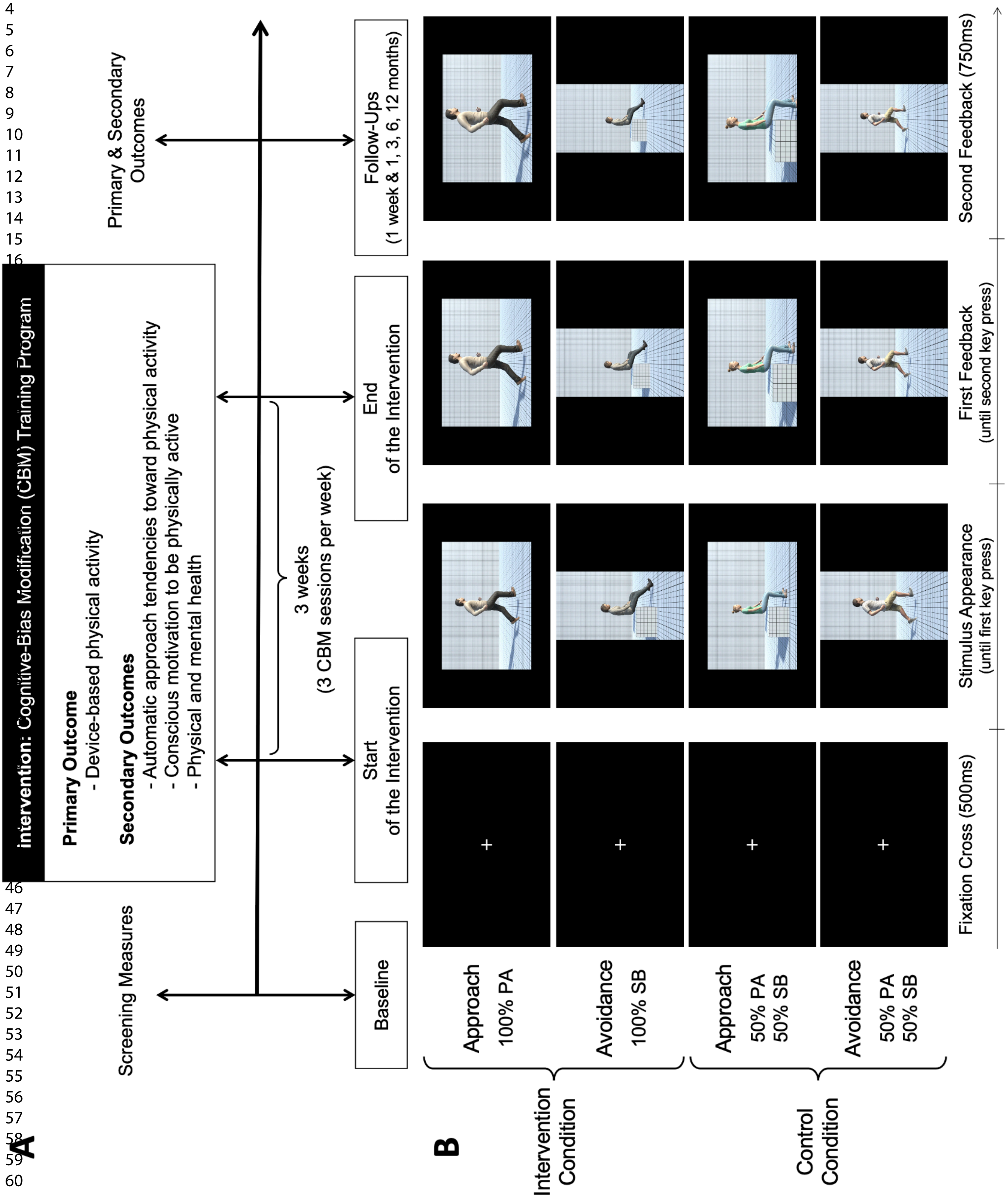
A
Intervention: Cognitive-Bias Modification (CBM) Training Program
Primary Outcome
- Device-based physical activity
Secondary Outcomes
- Automatic approach tendencies toward physical activity
- Conscious motivation to be physically active
- Physical and mental health
Screening Measures
Primary & Secondary Outcomes
3 weeks
(3 CBM sessions per week)
Baseline
Start of the Intervention
End of the Intervention
Follow-Ups
(1 week & 1, 3, 6, 12 months)
B
Intervention Condition
Control Condition
Approach 100% PA
Avoidance 100% SB
Approach 50% PA 50% SB
Avoidance 50% PA 50% SB
Fixation Cross (500ms)
Stimulus Appearance
(until first key press)
First Feedback
(until second key press)
Second Feedback (750ms)

Protocol Title: Improving physical activity (IMPACT) with the retraining of automatic actions tendencies: protocol of a randomized controlled clinical trial in an inpatient multidisciplinary rehabilitation program

Study Type:	Other Clinical Trial according to ClinO, Chapter 4
Risk Categorisation:	Risk category A acc. to ordinance HRO Art.7
Study Registration:	Promouvoir l'activité physique des patients en reprogrammant leurs réactions automatiques à l'aide de jeux sérieux sur ordinateur. L'essai randomisé contrôlé IMPACT.<br>Registration number: not applicable at this time
Sponsor:	University Hospital of Geneva / University of Geneva
Principal Investigators	PhD. Cheval Boris, Swiss National Science Foundation (SNSF) *Ambizione* fellowships, Swiss Center for Affective Sciences, Campus Biotech, University of Geneva, 9, Chemin des Mines, 1202 Geneva, Switzerland, Switzerland, phone +41223798942, e-mail: boris.cheval@unige.ch<br><br>Prof Christophe Luthy, MD, Chief Medical Officer, Internal Medicine and rehabilitation, Hospital “Beau-Séjour”, University Hospital of Geneva, 4, rue Gabrielle-Perret-Gentil, 1205 Geneva, Switzerland, Switzerland, phone +41223723545, e-mail: christophe.luthy@hcuge.ch<br><br>Prof Delphine Courvoisier, PhD, Bio-statistician, Quality of Care Unit, University Hospital of Geneva, 4, rue Gabrielle-Perret-Gentil, 1211 Geneva, Switzerland, Switzerland, phone +41223729008, e-mail: delphine.couvoisier@hcuge.ch
Investigated Intervention:	The new cognitive-bias modification (CBM) intervention consists of a 15-session training program performed over 3 weeks using computerized-based task in which participant are trained to approach pictures depicting physical activity and avoid pictures depicting sedentary behaviors, or a control intervention (sham training).The sham training condition (placebo) is the intervention against which the study intervention is evaluated.
Protocol ID	2019-02257
Version and Date:	Version 2 (dated 06/01/2020)

CONFIDENTIALITY STATEMENT

The information contained in this document is confidential and the property of the principal investigators. The information may not - in full or in part - be transmitted, reproduced, published, or disclosed to others than the applicable Competent Ethics Committee(s) and Regulatory

For peer review only - http://bmjopen.bmj.com/site/about/guidelines.xhtml

Authority(ies) without prior written authorisation from the sponsor except to the extent necessary to obtain informed consent from those who will participate in the study.

For peer review only

For peer review only - http://bmjopen.bmj.com/site/about/guidelines.xhtml

PROTOCOL SIGNATURE FORM

Study Title	Improving physical activity (IMPACT) with the retraining of automatic actions tendencies: protocol of a randomized controlled clinical trial in an inpatient multidisciplinary rehabilitation program
Study ID	2019-02257

The principal investigators have approved the protocol version 1 (dated 23/10/2019) and confirm hereby to conduct the study according to the protocol, current version of the World Medical Association Declaration of Helsinki, and ICH-GCP guidelines as well as the local legally applicable requirements.

Principal Investigator:

Name: CHEVAL, BORIS

Date: Genève, 06/01/2020__________ Signature:

For peer review only

For peer review only - http://bmjopen.bmj.com/site/about/guidelines.xhtml

TABLE OF CONTENTS

TABLE OF CONTENTS	4
GLOSSARY OF ABBREVATIONS	5
1 STUDY SYNOPSIS	6
2 BACKGROUND AND RATIONALE	8
3 STUDY OBJECTIVES AND DESIGN	10
3.1 Hypothesis and primary objective	10
3.2 Primary and secondary endpoints	10
3.3 Study design	10
3.4. Study intervention	11
4 STUDY POPULATION AND STUDY PROCEDURES	12
4.1 Inclusion and exclusion criteria, justification of study population	12
4.2 Recruitment, screening and informed consent procedure	12
4.3 Study procedures	12
4.4 Withdrawal and discontinuation	13
5 STATISTICS AND METHODOLOGY	13
5.1. Statistical analysis plan and sample size calculation	13
5.2. Handling of missing data and drop-outs	14
6 REGULATORY ASPECTS AND SAFETY	15
6.1 Local regulations / Declaration of Helsinki	15
6.2 (Serious) Adverse Events	15
6.3 (Periodic) safety reporting	16
6.4 Radiation	16
6.5 Pregnancy	16
6.6 Amendments	16
6.7 (Premature) termination of study	16
6.8 Insurance	17
7 FURTHER ASPECTS	17
7.1 Overall ethical considerations	17
7.2 Risk-benefit assessment	17
8 QUALITY CONTROL AND DATA PROTECTION	17
8.1 Quality measures	17
8.2 Data recording and source data	18
8.3 Confidentiality and coding	18
8.4 Retention and destruction of study data and biological material	19
9 MONITORING AND REGISTRATION	19
10. FUNDING / PUBLICATION / DECLARATION OF INTEREST	20
10 REFERENCES	20
References	21

For peer review only

For peer review only - http://bmjopen.bmj.com/site/about/guidelines.xhtml

GLOSSARY OF ABBREVATIONS

CBM	*Cognitive Bias Modification*
AE	*Adverse Event*
ASR/DSUR	*Annual Safety Repot / Development Safety Report*
BASEC	*Business Administration System for Ethical Committees*
CRF	*Case Report Form*
CTCAE	*Common Terminology Criteria for Adverse Events*
FADP	*Federal Act on Data Protection (in German: DSG, in French: LPD, in Italian: LPD)*
eCRF	*electronic Case Report Form*
FOPH	*Federal Office of Public Health*
GCP	*Good Clinical Practice*
HRA	*Human Research Act (in German: HFG, in French: LRH, in Italian: LRUm)*
ICH	*International Conference on Harmonisation*
ClinO	*Ordinance on Clinical Trials in Human Research (in German: KlinV, in French: OClin, in Italian: OSRUm)*
SAE	*Serious Adverse Event*

For peer review only

For peer review only - http://bmjopen.bmj.com/site/about/guidelines.xhtml

1 STUDY SYNOPSIS

**Sponsor / Sponsor-Investigator**	Swiss National Science Foundation /University Hospital of Geneva
**Study Title**	Improving physical activity (IMPACT) with the retraining of automatic actions tendencies: protocol of a randomized controlled clinical trial in an inpatient multidisciplinary rehabilitation program.
**Short Title / Study ID**	IMPACT / Study ID (not applicable at this time)
**Protocol Version and Date**	Version 2.0 from 06/01/2020
**Study Registration**	Promouvoir l'activité physique des patients en reprogrammant leurs réactions automatiques envers l'activité physique à l'aide de jeux sérieux sur ordinateur. L'essai randomisé contrôlé IMPACT.<br>The clinical trial will be registered in the Swiss Clinical Trials Portal (SNTCP) and in the EU Clinical Trials Register (EU-CTR).
**Study Category and Rationale**	Category A: minimal risk for the participants.
**Background and Rationale**	Being physically active is associated with a wide range of health benefits for rehabilitation inpatients, but it is challenging to maintain activity. Current interventions mainly rely on providing information about those benefits to increase motivation and develop the intention to be active. Yet, these interventions have proven to be rather ineffective in changing behaviors. Recent research in neuroscience demonstrated the significant involvement of automatic processes toward exercise-related stimuli in the regulation of physical activity behaviors, but they have not been applied to inpatients in rehabilitation.
**Risk / Benefit Assessment**	*Risks:* No specific risk.<br><br>*Benefits:* The intervention should help the participants to improve their physical activity level, thereby leading to potential health benefits.
**Objective(s)**	The **primary objective** is to evaluate the effect of a cognitive-bias modification (CBM) intervention on physical activity of inpatients multidisciplinary rehabilitation program.<br><br>The **secondary objectives** are to evaluate the effect of this CBM intervention on conscious motivation to be active, physical and mental health, and the use of healthcare system (number of days of hospitalization)
**Endpoint(s)**	Primary endpoints:<br>The primary outcomes will be time (in minutes) spent lying down, sit, standing-up, walking, and running, and the number of steps, the burned calories, the walking distance, and the total activity time, measured with the devices Polar.<br><br>Secondary endpoints:<br>The secondary outcomes will be the changes in (1) automatic action tendencies and conscious motivation to be active, (2) physical health, (3) mental health, and (4) the use of healthcare system.
**Study Design**	Single-center, placebo (sham-controlled), double-blinded, phase 3 randomized controlled trial.
**Statistical Considerations**	Analysis of variance, regression, and mixed-effects models will be used to assess the effect the intervention.
**Inclusion- / Exclusion Criteria**	Inclusion criteria: Patients treated in the ward 3DK of the General Medical Rehabilitation Division, aged 18 years or older, can comply with the study protocol, and able to provide a written consent of participation in the trial.<br><br>Exclusion criteria: Contraindication to physical activity in the view of the health status<br><br>Decision: The decision to include/exclude a participant from this study will be jointly decided by the chief medical officer and the research assistant.
**Number of Participants with Rationale**	Based on estimates of the effect size of automatic processes, a sample size calculation indicates that a minimum of 220 patients (110 per condition) is needed. We expect a loss to follow-up of 10 to 20% over one year. Thus, a total of 250 patients will be recruited.

For peer review only - http://bmjopen.bmj.com/site/about/guidelines.xhtml

**Study Intervention**	*Intervention group*. Patients in the intervention group will receive a training in which pictures depicting physical activity will be presented in the approach format, and pictures depicting sedentary behaviors will be presented in the avoidance format. Each training session will consist of 400 trials for a total duration of 15 minutes.
**Control Intervention**	*Comparator group.* Patients in the comparator group (placebo; sham-training) will receive a training in which pictures depicting physical activity and sedentary behaviors will be presented equally often in the approach and avoidance format. Each training session will consist of 400 trials for a total duration of 15 minutes.
**Study procedures**	Screening: Patients diseases and treatment characteristics, sociodemographic information, usual level of physical activity, and personality, as well as inclusion/exclusion criteria.<br><br>Intervention: The cognitive-bias (CM) intervention consists of a 15-sessions training program performed over 3 weeks using computerized-based task in which participant are trained to approach pictures depicting physical activity and avoid pictures depicting sedentary behaviors. Specifically, patients will be asked to react to the color of the square surrounding the pictures of physical activity and of sedentary behaviors by pressing four time the “move forward” or “move backward” key press to approach or avoid the pictures, respectively. Participants will be instructed to approach the picture when it will be surrounded by a green square, and to avoid it when the picture will be surrounded by a red square.<br><br>The primary outcome (devices-based measures of physical activity) will be measured during the intervention,. The secondary outcomes will be assessed before the intervention and at the beginning of each week during the intervention.
**Study Duration and Schedule**	Planned after ethics agreement (First-Participant-In)<br>Planned 01/2022 of Last-Participant-Out
**Investigator(s)**	PhD, **Boris Cheval**<br>9, Chemin des Mines, 1202 Geneva, Switzerland<br>Phone +41 22 379 89 42,<br>E-mail: boris.cheval@unige.ch<br><br>Prof, MD, **Christophe Luthy**<br>4 Rue Gabrielle-Perret-Gentil, 1205 Geneva, Switzerland<br>Phone +41 22 372 35 45,<br>E-mail: christophe.luthy@hcuge.ch<br><br>PhD., PD, **Delphine Courvoisier**<br>4 Rue Gabrielle-Perret-Gentil, 1211 Geneva, Switzerland<br>Phone +41223729008<br>E-mail: delphine.couvoisier@hcuge.ch
**Study Center(s)**	Division of General Medical Rehabilitation, Department of Rehabilitation and Geriatrics, University Hospital of Geneva, 4, rue Gabrielle-Perret-Gentil, 1205 Geneva, Switzerland, Switzerland
**Data privacy**	Data anonymization: Anonymity of the participants will be guaranteed when presenting the data at scientific meetings or publishing them in scientific journals, per CNIL recommendations. Individual participant information obtained as a result of this study is considered confidential and disclosure to third parties is prohibited. Subject confidentiality will be ensured by utilizing subject identification code numbers to correspond to treatment data in the computer files. Only a minority of personnel (i.e., the principal investigator and chief medical officer) will have access to the data in a non-coded form.
**Ethical consideration**	Scientific value of the project:<br>This project will test whether an intervention designed to directly target automatic reactions toward physical activity and sedentary behaviors, in addition to a traditional education intervention promoting physical activity to patients, can improve physical activity level in patients following a multidisciplinary rehabilitation program.<br><br>Social value of the project:<br>Physical activity is a key factor to improve the management of patient’s diseases recovery and health. Targeting automatic reactions toward physical activity, which may be particularly biased towards negative impressions of physical exercise among patients, is particularly innovative due to its low cost and its possibility to be implemented on a large scale (in and out-patients) to help individuals become more physically active, thereby improving physical fitness, quality of life and likely reduce the development of other co-morbidities. The findings from this study

For peer review only - http://bmjopen.bmj.com/site/about/guidelines.xhtml

	will provide evidence-based recommendations for a complementary intervention aiming to promote physical activity to patients in rehabilitation program.<br><br>Benefits/risks for the participants:<br>The investigator affirms and upholds the principle of the participant's right to dignity, privacy and health and that the project team shall comply with applicable privacy laws. Especially, anonymity of the participants will be guaranteed when presenting the data at scientific meetings or publishing them in scientific journals, per CNIL recommendations.<br><br>Methodology:<br>The placebo, double-blinded, phase 3 randomized controlled trials is appropriate to gain new generalizable knowledge on the effectiveness of a complementary intervention aiming to promote physical activity to patients in rehabilitation program.
**GCP Statement**	This study will be conducted in compliance with the protocol, the current version of the Declaration of Helsinki, the ICH-GCP, the HRA as well as other locally relevant legal and regulatory requirements.

2 BACKGROUND AND RATIONALE

The health benefits of physical activity are well established and extensive. Physical activity can reduce rates of cardiovascular disease,[1] cancers,[2] hypertension,[3] diabetes,[4] obesity,[5] depression,[6] and all cause of mortality,[7] even more effectively than medication.[8] Physical activity is safe and beneficial for almost everyone, while the risk of harm from moderate physical activity is small.[8 9] A recent systematic review and meta-analysis suggests that any level of and any intensities (including light intensity) of physical activity is beneficial.[7] In patients suffering from chronic diseases, increased physical activity is associated with reduced hospital admissions, decrease in pain, greater quality of life and mental health, and improvement in physical function.[8 10] These myriads of benefits even led the Academy of Medical Sciences to consider physical activity as a miracle cure.[11] Meanwhile, patients, similarly to the general population, remain nevertheless largely physically inactive.[12 13]

Healthcare professionals are uniquely placed to promote physical activity to patients. Current interventions to enhance physical activity in patients relies largely on providing rational information about the physical activity benefits. For example, a practical guide to help clinicians discuss about physical activity within a consultation has been recently proposed.[8] In that guide, clinicians are encouraged to rationally address patients' concerns about physical activity, to explain that there are more benefits to become active than to remain sedentary, to set an achievable goal, to identify barriers to be overcome, and finally to set a plan. This type of intervention is grounded on the dominant social-cognitive theories,[14] which contend that goals are proximal determinants of behaviors.[15 16] From these perspectives, changing patients' conscious goals should lead to substantial changes in their behaviors.[17 18] While these types interventions have proven to be effective to change physical activity behaviors to some extent,[19] meta-analyses also indicate that such rational interventions are more effective in changing intentions than in changing actual behavior.[20] Thus, new interventions targeting alternative mechanisms is needed.

Recent research on automatic factors may provide additional targets for interventions. Anchored within the dual-process models of behaviors,[21-23] this research suggests that physical activity is not only governed by "conscious" or reflective processes (e.g., intentions and attitudes towards physical activity), but also by "automatic processes" (e.g. automatic affective reactions) acting outside conscious awareness. For example, studies showed that in physically active individuals stimuli associated with physical activity attract attention,[24-27] trigger positive affective reactions,[28-31] and activate action tendencies to approach physical activity.[32-34] These automatic reactions are thought to facilitate the translation of conscious intentions into actual physical activity behaviors. As such, from this perspective, physical inactivity is thought to result from an imbalance between strong negative automatic reactions toward exercise and relatively weak intentions to be physically active. Crucially, this imbalance between automatic and reflective processes may be particularly pronounced in patients, who are more likely to spontaneously associate physical

For peer review only - http://bmjopen.bmj.com/site/about/guidelines.xhtml

activity with fear, as well as with the pain and discomfort felt during some exercises. Thus, in comparison with the general population, patients may demonstrate higher automatic negative reactions toward exercise, including, for instance, stronger negative affective reactions and weaker action tendencies to approach physical activity. The practical implications of these findings are that interventions designed to promote physical activity in patients might particularly benefit from directly targeting these automatic reactions toward physical activity.

What kinds of interventions are able to target automatic processes? Researchers have developed new types of interventions to directly target these automatic reactions toward a given health behavior.[35 36] For example, in alcohol addiction, studies have used a cognitive-bias modification (CBM) intervention aiming at retraining automatic approach reactions toward alcohol.[37] In this CBM intervention patients were repeatedly asked to pushing away alcohol-related pictures using a joystick simulating approach or avoidance movement. Specifically, in this computerized-based task participants were asked to push or pull a joystick in response to the format of the pictures. For example, they were instructed to make a push movement when the picture presented on the screen was tilted on the left, and to make a pull movement when the picture was tilted on the right. To ensure congruence with the participant' movements, the picture became smaller with the push movement, reflecting avoidance, while it became larger with the pull movement, reflecting approach. Participants received a training in which they have to push the joystick away in response to pictures of alcohol (i.e., all alcohol pictures were presented in the push format), and have to pull the joystick toward them in response to non-alcohol pictures (i.e., all non-alcohol were presented in the pull format). Two large studies conducted in patients showed that adding a CBM intervention to a regular treatment have a beneficial effect on the relapse rates one year after treatment discharge, with a reduction of 9%,[38] and 13%.[37] This improvement in the clinical effects was explained by change in approach tendencies.[38 39] These interventions have also proven to be useful in impacting cigarette smoking,[40] social anxiety,[41] or eating behaviors.[42-44]

To the best of our knowledge, however, only one study has been conducted to examine the effect of a brief CBM intervention on an exercise task in a sample of health young adults.[45] Specifically, using a manikin task,[46 47] a variant of the approach-avoidance joystick task, participants were trained to repeatedly approach a manikin toward pictures depicting physical activity and to avoid pictures depicting sedentary behaviors. Results revealed that participants spent more time exercising in a laboratory moderate intensity exercise task (i.e., doing squat), in comparison with control groups either trained to approach stimuli depicting sedentary behaviors and avoid stimuli depicting physical activity (i.e., reverse contingencies) or to approach and avoid stimuli depicting physical activity and sedentary behaviors equally often (sham training). These findings suggest that a single and brief CBM session targeting action tendencies to approach physical activity and sedentary behaviors can have beneficial effect of physical activity behaviors. However, this study has at least two important limitations. First, the physical activity behavior used as main outcome lack of ecological validity. Second, the study was conducted on a sample of rather physical active college students. As such the potential beneficial effect of adding a CBM intervention to a regular treatment in patients in rehabilitation program remains unknown.

In sum, automatic reactions toward exercise are crucial for the regulation of physical activity. However, current interventions designed to promote physical activity have mostly focused on changing reflective motivation, whereas automatic reactions have been rarely targeted. The IMPACT trial will assess, for the first time, whether an intervention designed to directly target automatic reactions toward physical activity and sedentary behaviors can improve physical activity levels in hospitalized patients following a multidisciplinary rehabilitation program.

Physical activity is a key factor to improve the management of patient's diseases recovery and health. Targeting automatic reactions toward physical activity, which may be particularly biased towards negative impressions of physical exercise among patients, is particularly innovative due to its low cost and its possibility to be implemented on a large

For peer review only

For peer review only - http://bmjopen.bmj.com/site/about/guidelines.xhtml

scale to help patients become more physically active, thereby improving physical fitness, quality of life and likely reduce the development of other co-morbidities. The findings from this study will provide evidence-based recommendations for a complementary intervention aiming to promote physical activity to patients in rehabilitation program.

3 STUDY OBJECTIVES AND DESIGN

3.1 Hypothesis and primary objective

The primary objective of this project is to investigate the effectiveness of a CBM intervention targeting automatic action tendencies toward physical activity and sedentary behaviors in an inpatient multidisciplinary rehabilitation program on physical activity behaviors. This trial will be performed using a sham-controlled, double-blinded randomized controlled trial. **The secondary objectives** are to evaluate the effect of this CBM intervention on conscious motivation to be active, physical and mental health, and the use of healthcare system (number of days of hospitalization). **We hypothesize** that the CBM intervention will be associated with higher levels of physical activity and lower levels of sedentary behaviors in patients during the rehabilitation program (H1). Moreover, we hypothesize that the CBM intervention will change patients' automatic approach bias toward physical activity and sedentary behaviors (H2). Finally, we predict that the CBM intervention will improve patients physical and mental health, and will reduce the use of healthcare system (H3).

3.2 Primary and secondary endpoints

Primary outcome measure:
The primary outcomes will be time (in minutes) spent lying down, sit, standing-up, walking, and running, and the number of steps, the burned calories, the walking distance, and the total activity time, measured with the devices Polar (Appendix 1 for an example of polar output).

Secondary outcomes:
The secondary outcomes will be changes in (1) automatic action tendencies and conscious motivation to be active, (2) physical health, (3) mental health, and (4) the use of healthcare system.

Baseline factors:
Patients diseases and treatment characteristics that may have an influence on the endpoints will be assessed in the screening procedure (e.g., demographics information, medical burden and co-morbidity, body mass index, mobility test, functional independence, health-related quality of life).

3.3 Study design

The IMPACT trial is a single-center, placebo (sham controlled), double-blinded, phase 3 randomized controlled trial. The double-blinded is made possible by the fact that the content of the intervention is difficult to identify during the task. The trial will start in after the ethics agreement (First-Participant-In) at the rehabilitation service of the Hospital "Beau-Séjour" of the University Hospital of Geneva (Switzerland) and will finish (Last-Participant-Out) in January 2022. Eligible patients will be randomly assigned to either the CBM intervention or the active control condition (sham training) in a 1:1 ratio. The current study follows the Standard Protocol Items: Recommendations for Interventional Trials (SPIRIT) statement.[48]

Pilot study:
A first pilot study has been conducted to assess the feasibility of an intervention targeting the automatic approach-avoidance tendencies toward physical activity and sedentary behaviors.[45]

For peer review only - http://bmjopen.bmj.com/site/about/guidelines.xhtml

This study demonstrates that, compared with participants from the control groups, participants trained to approach physical activity and avoid sedentary behaviors spent more time exercising in a laboratory moderate intensity exercise task. Although promising, this study was conducted on young and healthy students. Consequently, before running the prospective randomized controlled trial, we will first conduct a pilot study (n=30) at the rehabilitation service of the Hospital "Beau-Séjour" to secure the feasibility and acceptability of the current intervention among patients. This pilot study will start directly after the ethics agreement and will finish after thirtieth participants. The data collected will be analyzed, the potential changes to be made to the protocol will be discussed, and the decision to run the prospective randomized controlled trial will be jointly decided by the chief medical officer and the principal investigators.

Randomization and blinding:
The research assistants and the participants will be blinded to the allocation of the groups. At the end of the trial, the success of the participant blinding will be examined by asking to the participants to guess in what group there were, including a percentage of certainty.

The randomization will be generated on a computer and will be performed using permuted blocks (size = 4) and strata (size = 2). Specifically, the additional strata are used because the pictures used in the assessment phase (one-half of the pictures used in the assessment phase vs. second half of the pictures used in the assessment phase) will vary between the participants. To ensure that the research team will be blind on the randomization, an independent co-worker will carry out the randomization. The patient's identification number will be used to determine the sequence of randomization. Patients will be randomized in a 1:1 ratio between the intervention and active control condition.

3.4. Study intervention

All newly admitted patients will attend a meeting organized in the unit. The objective of this meeting will be to present and illustrate the health benefits of physical activity. Consistent with the recent practical guide to help healthcare professionals promoting physical activity to patients, research assistants will follow the "Ask-Assess-Advise" structure for discussing physical activity behavior change in the consultation. Patients will also receive a watch tracking and giving personalized feedback on their physical activity and sedentary behaviors. This procedure aims at increasing their positive attitudes toward physical activity and their intentions and motivation to be active, thereby allowing examining the additional effect of the CBM intervention.

Intervention group: Training program of 15 sessions over 3 weeks (i.e., 5 sessions by week on average) using an adapted version of visual approach/avoidance by the Self Task (VAAST),[49] a task that have shown to produce large and replicable effects. Specifically, patients will be asked to react to the color of the square surrounding the pictures of physical activity and of sedentary behaviors by pressing four time the "move forward" or "move backward" key press to approach or avoid the pictures, respectively. Participants will be instructed to approach the picture when it will be surrounded by a green square, and to avoid it when the picture will be surrounded by a red square. Congruent with the patient' approach or avoidance response, the whole visual environment will be zoomed in to simulate approach and zoomed out to simulate avoidance. A change by 10% after each key press will be used to give the impression to walk forward or backward as a consequence of the responses. Participants in the intervention group will receive a training in which all pictures depicting physical activity will be presented in the approach format, and all pictures depicting sedentary behaviors will be presented in the avoidance format. Each training session will consist of 400 trials for a total duration of 15 minutes. At the beginning of each week, this training phase will be preceded by 60 assessment trials in which the contingency of approaching or avoiding physical activity or sedentary behaviors will be 50% (i.e., participants will approach and avoid physical activity and sedentary behaviors equally often). That is, in this assessment phase, participants will be asked to approach and avoid both physical activity and

For peer review only - http://bmjopen.bmj.com/site/about/guidelines.xhtml

sedentary behaviors. This procedure will allow to measure the automatic approach (vs. avoidance) tendency toward physical activity and toward sedentary behaviors. See Wiers et al.,[37] for similar procedure.

Comparator group: Patients in the comparator group (placebo; sham-training) will not be trained to approach physical activity and to avoid sedentary behaviors. Specifically, the retraining sessions will also consist of 400 trials, but the task will require an equal number of approach and avoidance tractions to both stimuli depicting physical activity and sedentary behaviors.

Stimuli: Stimuli representing physical activity and sedentary behaviors will be created using the unity software. A set of 168 pictures including 14 avatars (50% women) in either active (walking and running) and inactive posture (sit on a cubicle) will be tested in a pilot study to identify the 60 pictures the most associated with associated with “movement and physically active behaviors” and the 60 pictures the most associated with “rest and physically inactive behaviors”. Pictures will be built to match for color, brightness, and visual complexity. The pictures will be also tested for valence and arousal (see appendix 2 for a sample of images).

To examine the generalization of training effects,[50] in both the intervention and comparator group, only half of the pictures used in the assessment phase will be included on the training phase (the selected pictures will be counterbalanced across participants).

4 STUDY POPULATION AND STUDY PROCEDURES

4.1 Inclusion and exclusion criteria, justification of study population

Participants fulfilling all the following **inclusion criteria** are eligible for the study:

- Patients treated in the ward 3DK of the General Medical Rehabilitation Division
- Aged 18 years or older
- Can comply with the study protocol
- Able to provide a written consent of participation in the trial.
-

The presence of the following **exclusion criteria** will lead to the exclusion of the participants:

- Contraindication to physical activity in the view of the health status

Decision to include/exclude a participant:
The decision to include/exclude a participant from this study will be jointly decided by the chief medical officer and the research assistant.

4.2 Recruitment, screening and informed consent procedure

All patients hospitalized in the ward 3DK following the rehabilitation program (from the beginning of 2020 (after the ethic committee agreement) to January 2022) will be approached during the first consultation with the chief medical officer and will receive an information sheet explaining the main objective of the IMPACT trial.

The investigators will explain to each participant the nature of the study, its purpose, the procedures involved, the expected duration, the potential risks and benefits and any discomfort it may entail. Each participant will be informed that the participation in the study is voluntary and that he or she may withdraw from the study at any time and that withdrawal of consent will not affect his or her subsequent medical assistance and treatment. The participant will be informed that his or her medical records may be examined by authorised individuals other than their treating physician.

All participants for the study will be provided a participant information sheet and a consent form describing the study and providing sufficient information for participant to make an informed decision about their participation in the study. Participants will have time to carefully read the

For peer review only - http://bmjopen.bmj.com/site/about/guidelines.xhtml

documents and can give their responses up to 24 hours after having receive the documents. Then, they will be randomized (1:1 ratio) to receive either the intervention or to the placebo (sham training).

The formal consent of a participant, using the approved consent form, will be obtained before the participant is submitted to any study procedure.

The consent form will be signed and dated by the investigator or his designee at the same time as the participant sign. A copy of the signed informed consent will be given to the study participant. The consent form will be retained as part of the study records.

All the measures that are not routine or daily practice (e.g., questionnaires on usual level of physical activity, temperaments) will be only performed once the informed consent has been obtained.

No compensation or payments will be given to the participants.

4.3 Study procedures

Study duration:
From after ethics agreement (First-Participant-In) to 01/2022 (Last-Participant-Out).

Screening:
Patients diseases and treatment characteristics, sociodemographic information, usual level of physical activity, and personality. Appendix 3 provides an overview of all the baseline screening measures available that may have an influence on the endpoints.

Intervention:
The intervention starts within two days of patient's arrival in the ward 3DK (that is, the necessary time to allow patients to carefully read the inform consent and to assist to the meeting aiming to increase their motivation to be active). The cognitive-bias (CBM) intervention consists of a 15-session training program performed over 3 weeks using computerized-based task in which participant are trained to approach pictures depicting physical activity and avoid pictures depicting sedentary behaviors. Specifically, patients will be asked to react to the color of the square surrounding the pictures of physical activity and of sedentary behaviors by pressing four time the "move forward" or "move backward" key press to approach or avoid the pictures, respectively. Participants will be instructed to approach the picture when it will be surrounded by a green square, and to avoid it when the picture will be surrounded by a red square. *In the intervention group*, patients will receive a training in which all pictures depicting physical activity will be presented in the approach format, and all pictures depicting sedentary behaviors will be presented in the avoidance format. *In the comparator group* (placebo; shame training), patients in the comparator group (placebo; sham-training) will receive a training in which pictures depicting physical activity and sedentary behaviors will be presented equally often in the approach ad avoidance format. As such, patients will not be trained to systematically approach physical activity and avoid sedentary behaviors. For both groups, each training session will consist of 400 trials for a total duration of 15 minutes.

The primary outcome:

The devices-based measures of physical activity (Polar) will be measured during the intervention.

The secondary outcomes:

The (1) automatic action tendencies and conscious motivation to be active, (2) physical health, (3) mental health, and (4) the use of healthcare system will be assessed before the intervention, and at the beginning of each week during the intervention. The outcome measures are described in more detail in appendix 4, and the timing of assessment in appendix 5. The study patient flow chart is provided in appendix 6.

For peer review only - http://bmjopen.bmj.com/site/about/guidelines.xhtml

4.4 Withdrawal and discontinuation

Participants will be informed that they can reconsider their decision to participate and withdraw from the study at any time without having to justify. This information will be explained in the informed consent form, but also orally by the research assistant at the beginning of each experimental sessions. The data will be conserved as data from participants prematurely withdrawing will be used for testing some research questions. However, participants will be informed that their data could be destroyed if they want. In such case, the data and consent forms will be properly deleted to ensure that information will not be recovered.

5 STATISTICS AND METHODOLOGY

5.1. Statistical analysis plan and sample size calculation

As recommended a statistician (Delphine Courvoisier) was consulted for the statistical analysis plan.

Statistical analyses will be performed according the intention-to-treat (ITT) principle and will abide by the Consolidated Standards of Reporting Trials (CONSORT) guidelines. Analysis will be conducted in a blinded way. We will used mean, standard deviation (SD), median, and range values to summarize the continuous data. The primary outcome will be analyzed using mixed effects models, which account for the nested structure of the data (i.e., multiple observations within a single participant), thereby providing accurate parameter estimates with acceptable Type I error rates.[51] To formally examine the short-term effect of the intervention on the evolution of physical activity within the rehabilitation period, models will include interaction terms between conditions (intervention group vs. comparator group) and number days within the rehabilitation program (linear and quadratic). The number of days should be relatively equal between patients (about 21 days), but may differ to some extent (some patients can leave earlier or other later than 21 days). A statistically significant interaction will indicate that the rate of physical activity change throughout the rehabilitation program is different across the conditions. The quadratic effect of number of days will be included to account for potential non-linear change of physical activity across the rehabilitation period. This will allow, for instance, modeling the possibility that the effect of the intervention will take some sessions before becoming effective or that no additional effect could be hoped after a certain number of sessions.

The continuous secondary outcomes will be treated in the similar way to the primary outcome. The number of days of hospitalization and the reliance of the healthcare system will analyzed using a regression with a Poisson distribution.

<u>Cost-benefits analysis</u>
Analyses will be also conduct to evaluate the cost-benefits of the CBM intervention. Interventions costs (staff, materials, etc…) will be estimated based on the median salary of a research assistant in the participating site and using the cost of 30 watch tracking (this quantity representing a reasonable number of watches to allow a unit to easily set up the intervention). Intervention benefits will be estimated based on the number of days of hospitalization during the rehabilitation program, as well as on the use of the medical staffs (time of nurses' and physicians' interventions) and expendables (bandages, compresses, etc.), compared to costs under treatment as usual. All analyses will be conducted using R software. Any deviation from the original statistical plan will be described and justified in the final trial report.

<u>Sample size:</u>
Based on estimates of the effect size of automatic processes,[44] a sample size calculation indicates that a minimum of 220 patients (110 per condition) is needed. We expect a loss to follow-up of 10 to 20% over one year. Thus, a total of 250 patients will be recruited.

For peer review only - http://bmjopen.bmj.com/site/about/guidelines.xhtml

Feasibility:
The ward 3DK of the General Medical Rehabilitation Division, has 24 beds and treats on average 40 patients per month. We expect that 1 patient out of 5 will not agree (for various reasons) to participate in the study, thereby leading to a total of about 30 participants recruited per month. As a consequence, we should be able to collect the target sample size in approximately 8-10 months.

5.2. Handling of missing data and drop-outs

Sporadic missing values will be imputed using multiple imputations with chained equation. Additionally, an analysis of the enrollment issues at the mid-point of the study will allow to check for data collection issues and, if loss to follow-up is higher than expected, to readjust the number of participants to recruit. As stressed in the section above, we already plan to recruit 30 additional participants to compensate for lost to follow-up.

6 REGULATORY ASPECTS AND SAFETY

6.1 Local regulations / Declaration of Helsinki

This study is conducted in compliance with the protocol, the current version of the Declaration of Helsinki, the ICH-GCP, the HRA as well as other locally relevant legal and regulatory requirements.

6.2 (Serious) Adverse Events

An Adverse Event (AE) is any untoward medical occurrence in a patient or a clinical investigation subject which does not necessarily have a causal relationship with the trial procedure. An AE can therefore be any unfavourable or unintended finding, symptom, or disease temporally associated with a trial procedure, whether or not related to it.

A Serious Adverse Event (SAE) (ClinO, Art. 63) is any untoward medical occurrence that

- Results in death or is life-threatening,
- Requires in-patient hospitalisation or prolongation of existing hospitalisation,
- Results in persistent or significant disability or incapacity, or
- Causes a congenital anomaly or birth defect

Both Investigator and Sponsor-Investigator make a causality assessment of the event to the trial intervention (see table below based on the terms given in ICH E2A guidelines). Any event assessed as possibly, probably or definitely related is classified as related to the trial intervention.

Relationship	Description
Definitely	Temporal relationship<br>Improvement after dechallenge*<br>Recurrence after rechallenge<br>(or other proof of drug cause)
Probably	Temporal relationship<br>Improvement after dechallenge<br>No other cause evident
Possibly	Temporal relationship<br>Other cause possible

For peer review only - http://bmjopen.bmj.com/site/about/guidelines.xhtml

Unlikely	Any assessable reaction that does not fulfil the above conditions
Not related	Causal relationship can be ruled out
*Improvement after dechallenge only taken into consideration, if applicable to reaction	

Both Investigator and Sponsor-Investigator make a severity assessment of the event as mild, moderate or severe. Mild means the complication is tolerable, moderate means it interferes with daily activities and severe means it renders daily activities impossible.

Reporting of SAEs (see ClinO, Art. 63)

All SAEs are documented and reported immediately (within a maximum of 24 hours) to the Sponsor-Investigator of the study.

If it cannot be excluded that the SAE occurring in Switzerland is attributable to the intervention under investigation, the Investigator reports it to the Ethics Committee via BASEC within 15 days.

Follow up of (Serious) Adverse Events

No adverse event resulting from the intervention is expected. Yet, the reasons for participants terminating the study will be reported in the eCRF, as such it will be possible to determine whether the termination of the study is due to a serious adverse event such as a transfer in the emergency unit or a death.

6.3 (Periodic) safety reporting

An annual safety report (ASR/DSUR) is submitted once a year to the local Ethics Committee by the Investigator (ClinO, Art. 43 Abs).

The project leader is promptly notified (within 24 hours) if immediate safety and protective measures have to be taken during the conduct of the research project. The Ethics Committee will be notified via BASEC of these measures and of the circumstances necessitating them within 7 days.

6.4 Radiation

None.

6.5 Pregnancy

Not applicable.

6.6 Amendments

Substantial changes to the study setup and study organization, the protocol and relevant study documents will be submitted to the Ethics Committee for approval before implementation. Under emergency circumstances, deviations from the protocol to protect the rights, safety and well-being of human subjects will be proceed without prior approval of the Ethics Committee. Such deviations will be documented and reported to the Ethics Committee as soon as possible.

Substantial amendments are changes that affect the safety, health, rights and obligations of participants, changes in the protocol that affect study objective(s) or central research topic, changes of study site(s) or of study leader and sponsor (ClinO, Art. 29).

6.7 (Premature) termination of study

The principal investigator or any other competent authority may terminate the study prematurely according to the following circumstances:

- Ethical concerns
- Insufficient participants recruitment
- Early evidence of harm of benefit of the experimental intervention (interim analysis).

For peer review only - http://bmjopen.bmj.com/site/about/guidelines.xhtml

Upon regular study termination, the Ethics Committee is notified via BASEC within 90 days (ClinO, Art. 38).

Upon premature study termination or study interruption, the Ethics Committee is notified via BASEC within 15 days (ClinO, Art. 38).

All the health-related data will be anonymized upon end of the study.

6.8 Insurance

In the event of study-related damage or injuries, the liability of University Hospitals of Geneva and of the University of Geneva (via is insurance contract conduced with AXA Winterthur company) provides compensation, except for claims that arise from misconduct or gross negligence.

7 FURTHER ASPECTS

7.1 Overall ethical considerations

Scientific value of the project:
This project will test whether an intervention design to directly automatic reactions toward physical activity and sedentary behaviors, in addition to a traditional education intervention promoting physical activity to patients, can improve physical activity level in patients following a multidisciplinary rehabilitation program.

Social value of the project:
Physical activity is a key factor to improve the management of patient's diseases recovery and health. Targeting automatic reactions toward physical activity, which may be particularly biased towards negative impressions of physical exercise among patients, is particularly innovative due to its low cost and possibility to be implemented on a large scale to help patients become more physically active, thereby improving physical fitness, quality of life and likely reduce the development of other co-morbidities. The findings from this study will provide evidence-based recommendations for a complementary intervention aiming to promote physical activity to patients in rehabilitation program.

Perspectives:
Developing an online, home-based, computerized intervention targeting automatic reactions toward exercise in order to reach a larger population living at home.

Project-specific ethical aspects:

The investigator affirms and upholds the principle of the participant's right to dignity, privacy and health and that the project team shall comply with applicable privacy laws. Especially, anonymity of the participants will be guaranteed when presenting the data at scientific meetings or publishing them in scientific journals, per CNIL recommendations.

7.2 Risk-benefit assessment

Risks:
No specific risk expected.

Benefits:

For peer review only

For peer review only - http://bmjopen.bmj.com/site/about/guidelines.xhtml

The intervention should help the participants to improve their physical activity level, thereby leading to potential health benefits.

8 QUALITY CONTROL AND DATA PROTECTION

8.1 Quality measures

The principal investigator will organize a proper training of all involved study personnel to ensure that the study will be conducted according to the protocol. Research assistants should understand the detailed contents of the protocol before starting the data collection.

For quality assurance the sponsor, the Ethics Committee or an independent trial monitor may visit the research sites. Direct access to the source data and all study related files is granted on such occasions. All involved parties keep the participant data strictly confidential.

8.2 Data recording and source data

This project will generate 5 main types of raw data.

1. Clinical data routinely assessed during clinical practice
2. Questionnaires for baseline screening measures
3. Devices based-measures of physical activity and sedentary behavior
4. E-prime data associated with the completion of each training session
5. Questionnaires measuring the secondary outcomes

All data will be stored in the format in which it was originally generated are not expected to exceed 200 M0. Then, all the data will be converted into spreadsheets. The five main types of raw data will be stored in separated spreadsheets. On these spreadsheets the participants are only identified by a unique participant number. The principal investigator will be in charge to merge all these data in a unique spreadsheet using the merge function of the R software to avoid any errors in the constitution of this unique datafile. Moreover, the correspondence between each unique spreadsheet and the common spreadsheet will be manually checker by two research assistants.

The dataset will be accompanied by a README file, which will describe the directory hierarchy and file naming convention. The directory will contain an INFO file describing the experimental protocol used in that experiment. This INFO file will also record any deviations from the protocol and other useful contextual information. This procedure should allow the data to be easily understood by other researchers and should support future reuse on the data. For each experiment a metadata will be created to provide contextual information required to interpret data. This metadata file will be created in accordance with the Data Documentation Initiative (DDI). In particular, the metadata file will include short unique identifier, the name of the author(s), the content, the date of creation, the locations, the reason why the data was generated, and how the data was created. The codebook will explicitly indicate the name, explanations, and the modalities of the different variables measured in the experiment. In addition, it will include information on the study design and contain all information necessary for another analyst to use the data accurately. The metadata will follow the existing community standard and convention.

8.3 Confidentiality and coding

Trial and participant data will be handled with uttermost discretion and is only accessible to authorised personnel who require the data to fulfil their duties within the scope of the study. On the CRFs and other study specific documents, participants are only identified by a unique participant number.

For peer review only - http://bmjopen.bmj.com/site/about/guidelines.xhtml

Data anonymization:
Anonymity of the participants will be guaranteed when presenting the data at scientific meetings or publishing them in scientific journals, per CNIL recommendations. Individual participant information obtained as a result of this study is considered confidential and disclosure to third parties is prohibited. Subject confidentiality will be ensured by utilizing subject identification code numbers to correspond to treatment data in the computer files. Only a minority of personnel (i.e., the principal investigator and chief medical officer) will have access to the data in a non-coded form.

Data storage:
Participant data on a secure database in accordance with the General Data Protection Regulations (2018). Three copied of the data will be stored. First, original data will be stored on the principal investigator' computer, which will be backed up daily, and protected by a password. Additionally, data will be stored on a secure server hosted by the University of Geneva. Finally, data will be stored on an external device at a different location and password protected. Original notebook will be stored in the principal investigator' laboratory. Local version of the data for statistical analysis will remain on a University computer, and be password protected. Each person who collected the data will have the responsibility to annotate their data within the metadata. Nevertheless, the principal investigator will have the responsibility to weekly check that the data is properly processed, documented, and stored. All study data will be archived for a minimum of 10 years beyond the end of the randomized controlled trial.

No biological material is collected in this study.

8.4 Retention and destruction of study data and biological material

All study data are archived for 10 years after study termination or premature termination of the study.

Data Sharing and reuse:
Datasets and metadata from this trial will be deposited in ZENODO (a generic and free repository based at CERN, Geneva) for a duration of 20 years, and made public at the time of publication. Data in the repository will be stored in accordance with funder and University data policies. In particular original data sets, original software script and code, and original raw data will be deposited. However, as stressed above, personal data will be anonymized before diffusion, per CNIL recommendations.

In addition, the data sets and publications will be also deposited in Archive ouverte UNIGE.

Files deposited in ZENODO will be given a Digital Object Identifier (DOI). The retention schedule for data in ZENODO will be 20 years from date of deposition in the first instance.

The DOI issued to data sets in the repository can be included as part of a data citation in publications, allowing the data sets underpinning a publication to be identified and accessed.

9 MONITORING AND REGISTRATION

The principal investigator will weekly check the storage of the five main types of raw data. He will ensure that a copy of the original data is stored daily on an external device as well as stored on a secure server hosted by the University of Geneva. Additionally, a research assistant working at the University Hospital of Geneva, not involved in the data collection, will check the monitoring duties are correctly done. All the anonymized data and documents will be accessible to monitors and questions will be answered during the monitoring.

For peer review only - http://bmjopen.bmj.com/site/about/guidelines.xhtml

The IMPACT trial will be registered in French in the Swiss National Clinical trial Portal (SNCTP). Moreover, the study will be registered in the EU Clinical Trials Register (EU-CTR; https://www.clinicaltrialsregister.eu), a registry listed in the WHO International Clinical Trials Registry Platform (ICTRP; http://www.who.int/ictrp/en/).

10. FUNDING / PUBLICATION / DECLARATION OF INTEREST

The current project is related to the clinical part of a project funded by the Swiss National Science Foundation (699'915 CHF). Project Ambizione PZ00P1_180040.
The purchase of protocol-related material (i.e. 30 Polar watches) is provided by the non-operating fund of the Division of General Medical Rehabilitation of Beau-Séjour. The computer equipment (3 laptops) will be made available by the Division of General Medical Rehabilitation of Beau-Séjour, Department of Rehabilitation and Geriatrics, University Hospital of Geneva.

There is no conflict of interest.

The protocol will be pre-registered and for the interest of research transparency the data will be published whether the hypotheses were confirmed or not.

10 REFERENCES

1. Common Terminology Criteria for Adverse Events (CTCAE) https://www.eortc.be/services/doc/ctc/CTCAE_4.03_2010-06-14_QuickReference_5x7.pdf
2. Declaration of Helsinki https://www.wma.net/policies-post/wma-declaration-of-helsinki-ethical-principles-for-medical-research-involving-human-subjects/
3. Federal Act on Data Protection (FADP) https://www.admin.ch/opc/en/classified-compilation/19920153/index.html
4. Human Research Act (HRA) https://www.admin.ch/opc/de/classified-compilation/20061313/index.html
5. International Conference on Harmonization (ICH) E6(R2) Guideline for Good Clinical Practice http://www.ich.org/fileadmin/Public_Web_Site/ICH_Products/Guidelines/Efficacy/E6/E6_R2__Step_4_2016_1109.pdf
6. International Conference on Harmonization (ICH) E2A Clinical Safety Data Management: Definitions and Standards for Expedited Reporting http://www.ema.europa.eu/docs/en_GB/document_library/Scientific_guideline/2009/09/WC500002749.pdf
7. Ordinance on Clinical Trials in Human Research (ClinO) https://www.admin.ch/opc/de/classified-compilation/20121176/index.html

For peer review only

For peer review only - http://bmjopen.bmj.com/site/about/guidelines.xhtml

References

1. Wahid A, Manek N, Nichols M, et al. Quantifying the association between physical activity and cardiovascular disease and diabetes: a systematic review and meta‐analysis. *Journal of the American Heart Association* 2016;5(9):e002495.
2. Moore SC, Lee I-M, Weiderpass E, et al. Association of leisure-time physical activity with risk of 26 types of cancer in 1.44 million adults. *JAMA internal medicine* 2016;176(6):816-25.
3. Liu X, Zhang D, Liu Y, et al. Dose–response association between physical activity and incident hypertension: a systematic review and meta-analysis of cohort studies. *Hypertension* 2017;69(5):813-20.
4. Aune D, Norat T, Leitzmann M, et al. Physical activity and the risk of type 2 diabetes: a systematic review and dose–response meta-analysis: Springer, 2015.
5. Bleich SN, Vercammen KA, Zatz LY, et al. Interventions to prevent global childhood overweight and obesity: a systematic review. *The Lancet Diabetes & Endocrinology* 2018;6(4):332-46.
6. Schuch F, Vancampfort D, Firth J, et al. Physical activity and sedentary behavior in people with major depressive disorder: a systematic review and meta-analysis. *Journal of affective disorders* 2017;210:139-50.
7. Ekelund U, Tarp J, Steene-Johannessen J, et al. Dose-response associations between accelerometry measured physical activity and sedentary time and all cause mortality: systematic review and harmonised meta-analysis. *bmj* 2019;366:l4570.
8. Haseler C, Crooke R, Haseler T. Promoting physical activity to patients. *BMJ* 2019;366:l5230.
9. Godlee F. The miracle cure: British Medical Journal Publishing Group, 2019.
10. Anderson L, Oldridge N, Thompson DR, et al. Exercise-based cardiac rehabilitation for coronary heart disease: Cochrane systematic review and meta-analysis. *Journal of the American College of Cardiology* 2016;67(1):1-12.
11. Exercise: the miracle cure and the role of the doctor in promoting it. https://www.aomrc.org.uk/wp-content/uploads/2016/05/Exercise_the_Miracle_Cure_0215.pdf. 2015
12. Johansen KL, Chertow GM, Ng AV, et al. Physical activity levels in patients on hemodialysis and healthy sedentary controls. *Kidney international* 2000;57(6):2564-70.
13. Watz H, Waschki B, Meyer T, et al. Physical activity in patients with COPD. *European Respiratory Journal* 2009;33(2):262-72.
14. Rhodes RE, Nigg CR. Advancing physical activity theory: A review and future directions. *Exerc Sport Sci Rev* 2011;39(3):113-19.
15. Bandura A. Self-efficacy: The exercise of control (1st edition). . New York: Worth Publishers 1997.
16. Carver CS, Scheier MF. On the self-regulation of behavior. New York:: Cambridge University Press 1998.
17. Sheeran P, Gollwitzer PM, Bargh JA. Nonconscious processes and health. *Health Psychol* 2013;32(5):460-73. doi: 10.1037/a0029203
18. Brand R, Cheval B. Theories to explain exercise motivation and physical inactivity: Ways of expanding our current theoretical perspective. *Front Psychol* 2019;10:1147.
19. Hillsdon M, Foster C, Thorogood M. Interventions for promoting physical activity: Europe PMC Funders, 2005.

For peer review only

For peer review only - http://bmjopen.bmj.com/site/about/guidelines.xhtml

20. Rhodes RE, Dickau L. Experimental evidence for the intention–behavior relationship in the physical activity domain: a meta-analysis. *Health Psychol* 2012;31:724-27.
21. Evans JSB. Dual-processing accounts of reasoning, judgment, and social cognition. *Annu Rev Psychol* 2008;59:255-78.
22. Hofmann W, Friese M, Wiers RW. Impulsive versus reflective influences on health behavior: A theoretical framework and empirical review. *Health Psychol Rev* 2008;2(2):111-37.
23. Strack F, Deutsch R. Reflective and impulsive determinants of social behavior. *Pers Soc Psychol Rev* 2004;8(3):220-47.
24. Berry TR. Who's even interested in the exercise message? Attentional bias for exercise and sedentary-lifestyle related words. *J Sport Exerc Psychol* 2006;28(1):4-17.
25. Berry TR, Spence JC, Stolp SM. Attentional bias for exercise-related images. *Res Q Exerc Sport* 2011;82(2):302-9.
26. Calitri R, Lowe R, Eves FF, et al. Associations between visual attention, implicit and explicit attitude and behaviour for physical activity. *Psychol Health* 2009;24(9):1105-23.
27. Cheval B, Miller MW, Orsholits D, et al. Physically active individuals look for more: An eye tracking study of attentional bias. *SportArxiv* 2019
28. Conroy DE, Hyde AL, Doerksen SE, et al. Implicit attitudes and explicit motivation prospectively predict physical activity. *Ann Behav Med* 2010;39(2):112-18.
29. Rebar AL, Ram N, Conroy DE. Using the EZ-diffusion model to score a single-category implicit association test of physical activity. *Psychology of Sport & Exercise* 2015;16:96-105.
30. Bluemke M, Brand R, Schweizer G, et al. Exercise might be good for me, but I don't feel good about it: do automatic associations predict exercise behavior? *Journal of Sport and Exercise Psychology* 2010;32(2):137-53.
31. Chevance G, Caudroit J, Romain AJ, et al. The adoption of physical activity and eating behaviors among persons with obesity and in the general population: the role of implicit attitudes within the Theory of Planned Behavior. *Psychol Health Med* 2017;22(3):319-24.
32. Hannan TE, Moffitt RL, Neumann DL, et al. Implicit approach–avoidance associations predict leisure-time exercise independently of explicit exercise motivation. *Sport Exerc Perform Psychol* 2019;8(2):210-22.
33. Cheval B, Sarrazin P, Isoard-Gautheur S, et al. Reflective and impulsive processes explain (in)effectiveness of messages promoting physical activity: a randomized controlled trial. *Health Psychol* 2015;34(1):10-19.
34. Cheval B, Sarrazin P, Isoard-Gautheur S, et al. How impulsivity shapes the interplay of impulsive and reflective processes involved in objective physical activity. *Pers Individ Dif* 2016;96:132-7.
35. Friese M, Hofmann W, Wiers RW. On taming horses and strengthening riders: Recent developments in research on interventions to improve self-control in health behaviors. *Self and Identity* 2011;10(3):336-51.
36. Marteau TM, Hollands GJ, Fletcher PC. Changing human behavior to prevent disease: the importance of targeting automatic processes. *Science* 2012;337(6101):1492-95.
37. Wiers RW, Eberl C, Rinck M, et al. Retraining automatic action tendencies changes alcoholic patients' approach bias for alcohol and improves treatment outcome. *Psychol Sci* 2011;22(4):490-97.

For peer review only - http://bmjopen.bmj.com/site/about/guidelines.xhtml

38. Eberl C, Wiers RW, Pawelczack S, et al. Approach bias modification in alcohol dependence: do clinical effects replicate and for whom does it work best? *Developmental cognitive neuroscience* 2013;4:38-51.
39. Gladwin TE, Rinck M, Eberl C, et al. Mediation of cognitive bias modification for alcohol addiction via stimulus ‐ specific alcohol avoidance association. *Alcoholism: Clinical and Experimental Research* 2015;39(1):101-07.
40. Wittekind CE, Feist A, Schneider BC, et al. The approach-avoidance task as an online intervention in cigarette smoking: A pilot study. *Journal of behavior therapy and experimental psychiatry* 2015;46:115-20.
41. Taylor CT, Amir N. Modifying automatic approach action tendencies in individuals with elevated social anxiety symptoms. *Behaviour research and therapy* 2012;50(9):529-36.
42. Fishbach A, Friedman RS, Kruglanski AW. Leading us not unto temptation: Momentary allurements elicit overriding goal activation. *J Pers Soc Psychol* 2003;84(2):296-309. doi: 10.1037/0022-3514.84.2.296
43. Fishbach A, Shah JY. Self-control in action: implicit dispositions toward goals and away from temptations. *J Pers Soc Psychol* 2006;90(5):820.
44. Aulbach MB, Knittle K, Haukkala A. Implicit process interventions in eating behaviour: a meta-analysis examining mediators and moderators. *Health psychology review* 2019;13(2):179-208.
45. Cheval B, Sarrazin P, Pelletier L, et al. Effect of retraining approach-avoidance tendencies on an exercise task: A randomized controlled trial. *J Phys Act Health* 2016;13(12):1396-403.
46. Cheval B, Sarrazin P, Pelletier L. Impulsive approach tendencies towards physical activity and sedentary behaviors, but not reflective intentions, prospectively predict non-exercise activity thermogenesis. *Plos One* 2014;9(12):e115238. doi: 10.1371/Journal.Pone.0115238
47. Krieglmeyer R, Deutsch R. Comparing measures of approach–avoidance behaviour: The manikin task vs. two versions of the joystick task. *Cognition and emotion* 2010;24(5):810-28.
48. Chan A-W, Tetzlaff JM, Altman DG, et al. SPIRIT 2013 statement: defining standard protocol items for clinical trials. *Annals of internal medicine* 2013;158(3):200-07.
49. Rougier M, Muller D, Ric F, et al. A new look at sensorimotor aspects in approach/avoidance tendencies: The role of visual whole-body movement information. *Journal of Experimental Social Psychology* 2018;76:42-53.
50. Wiers RW, Rinck M, Kordts R, et al. Retraining automatic action ‐ tendencies to approach alcohol in hazardous drinkers. *Addiction* 2010;105(2):279-87.
51. Boisgontier MP, Cheval B. The anova to mixed model transition. *Neurosci Biobehav Rev* 2016
52. Grimby G, Börjesson M, Jonsdottir I, et al. The “Saltin–Grimby Physical Activity Level Scale” and its application to health research. *Scandinavian journal of medicine & science in sports* 2015;25:119-25.
53. Gosling SD, Rentfrow PJ, Swann Jr WB. A very brief measure of the Big-Five personality domains. *J Res Pers* 2003;37(6):504-28.
54. Lowe R, Eves F, Carroll D. The influence of affective and instrumental beliefs on exercise intentions and behavior: A longitudinal analysis. *Journal of Applied Social Psychology* 2002;32(6):1241-52.
55. French DP, Sutton S, Hennings SJ, et al. The Importance of Affective Beliefs and Attitudes in the Theory of Planned Behavior: Predicting Intention to Increase Physical Activity 1. *Journal of Applied Social Psychology* 2005;35(9):1824-48.

For peer review only

For peer review only - http://bmjopen.bmj.com/site/about/guidelines.xhtml

56. Leong DP, Teo KK, Rangarajan S, et al. Prognostic value of grip strength: findings from the Prospective Urban Rural Epidemiology (PURE) study. *Lancet* 2015;386(9990):266-73.

For peer review only

For peer review only - http://bmjopen.bmj.com/site/about/guidelines.xhtml

Appendix 1: Example of a polar' output (Heathy individual)

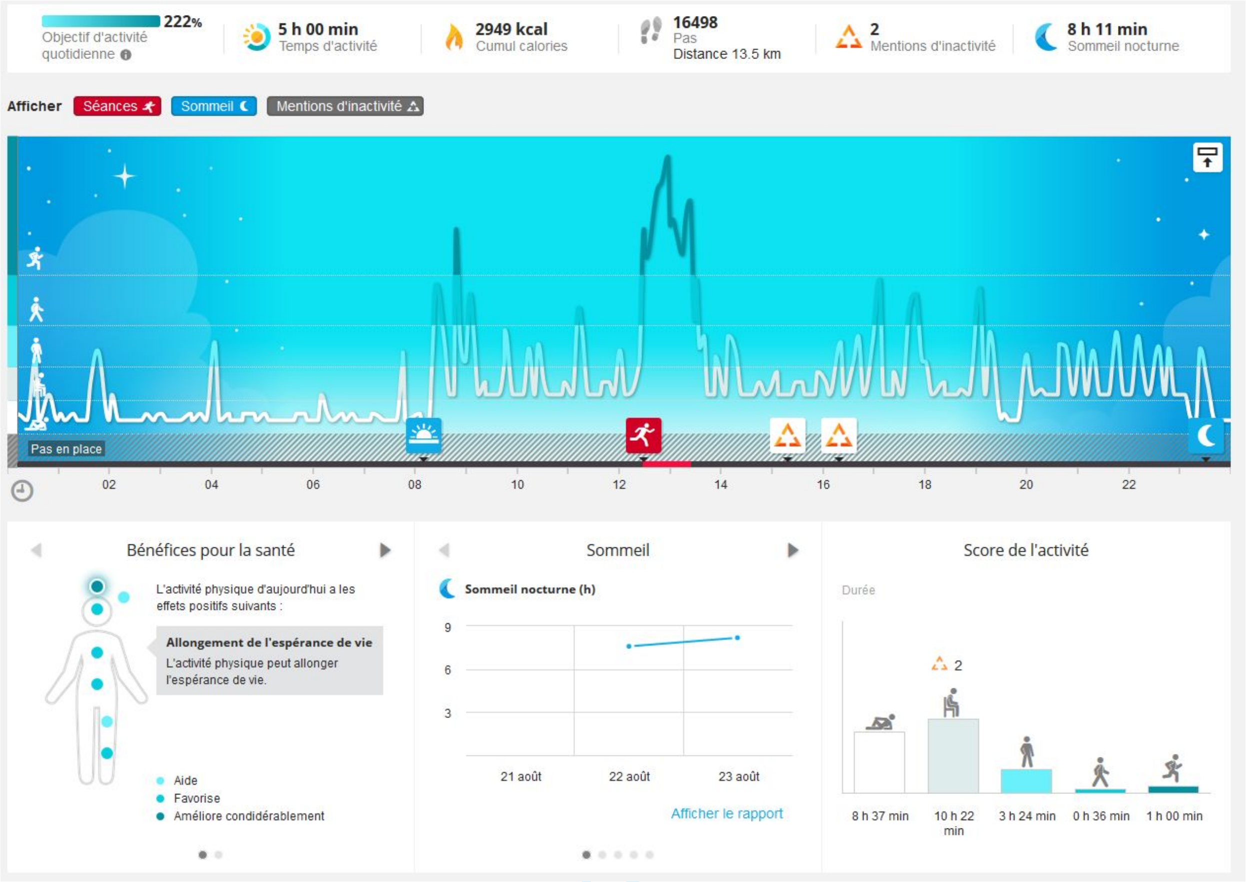

For peer review only - http://bmjopen.bmj.com/site/about/guidelines.xhtml

Appendix 2: Sample of images used in the approach-avoidance task

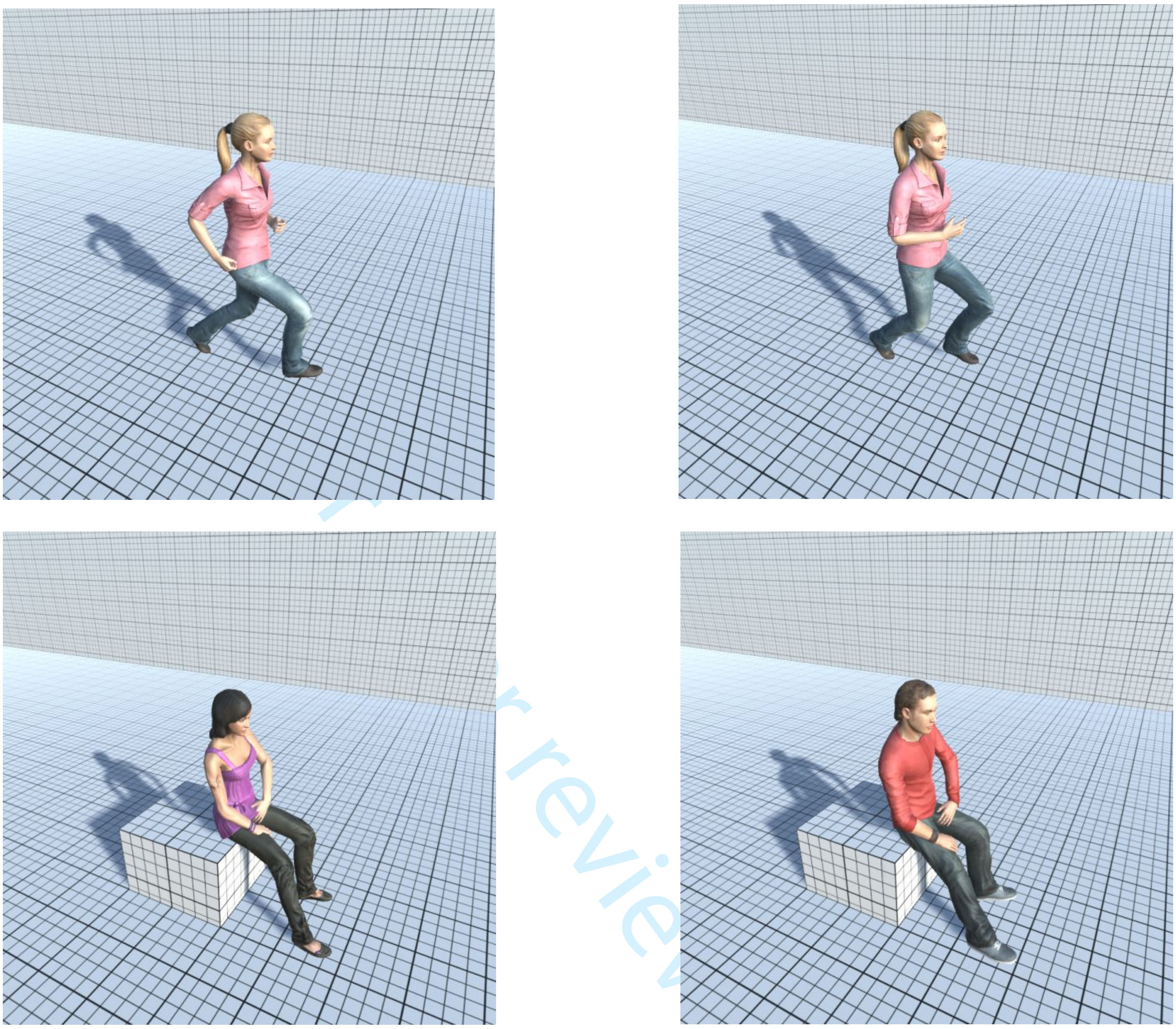

For peer review only

For peer review only - http://bmjopen.bmj.com/site/about/guidelines.xhtml

Appendix 3: Overview of the baseline screening measures

Measures	Assessment method
**Inclusion criteria**	
Patients treated in ward 3DK of the General Medical Rehabilitation Division.	During the first meeting with the research assistant
≥ 18years of age	
Can comply with study protocol	
Able to provide a written consent	
**Exclusion criteria**	
Contraindication to physical activity in the view of the health status	During the first meeting with the research assistant
**Additional baseline screening assessment**	
Medical evaluation (Questionnaires and objective tests)	Patients diseases and treatment characteristics (medical burden, co-morbidity, body mass index, mobility test, functional independence, health-related quality of life)
Sociodemographic characteristics	Questionnaires
Usual level of physical activity	The Saltin-Grimby Physical Activity Level Scale (SGPALS)[52]
Personality	Ten-Item Personality Inventory (TIPI)[53]

For peer review only

For peer review only - http://bmjopen.bmj.com/site/about/guidelines.xhtml

Appendix 4: Outcomes measures of the IMPACT trial

Outcome	Assessment method
**Primary outcome**	
Physical activity and sedentary behaviors	Devices-based measures (Polar) assessing the time spent lying down, sit, standing-up, walking, and running, as well as the number of steps and the total activity time.
**Secondary outcomes**	
Reflective and automatic precursors of physical activity	
Attitudes	Instrumental (i.e., useful, beneficial) and affective (i.e., enjoyable, interesting) attitudes toward physical activity using a short, self-reported questionnaire.[54 55]
Approach tendencies	The visual approach/avoidance by the Self Task (VAAST).[49] A reaction time task assessing automatic approach tendencies toward physical activity and sedentary behaviors
Physical Health	
Weight	Weight (accuracy 0.1 kg) was assessed with participants clothed (lightweight clothing)
Muscle strength	Grip strength measured with a handheld dynamometer.[56]
Perceived global physical health	Global physical health Patient-Reported Outcomes Measurement Information System (PROMIS)[52] scale.
Mental health	
Perceived physical functioning	
Perceived pain-interference	Pain interference and pain intensity PROMIS scales.
Depression, anxiety, general life satisfaction	Anxiety, depression, general life satisfaction PROMIS scales.
Self-efficacy	Self-efficacy for managing chronic conditions PROMIS scales.
Social role	Ability to participants in social roles and activities PROMIS scale.
Healthcare system uses	
Hospitalization	Number of days of hospitalization during the rehabilitation program
Use of human resources and expendables	Time spent by healthcare professionals with the patient and quantity of expendables (bandages, compresses, etc.) used.

For peer review only - http://bmjopen.bmj.com/site/about/guidelines.xhtml

Appendix 5: Schedule of assessment

WEEK 1	>-1 day	0	+1	+2	+3	+4	+5
Visit	Information	Screening	1st training session	2nd training session	3th training session	4th training session	5th training session
Oral and written patient information	+						
Written consent		+					
Inclusion-/ exclusion criteria		+					
Additional baseline screening assessment		+					
Self-reported physical activity (usual week)		+					
Intervention			+	+	+	+	+
Motivation to be active			+				
Approach tendencies			+				
Physical health			+				
Mental health			+				

WEEK 2	+1	+2	+3	+4	+5
Visit	1st training session	2nd training session	3th training session	4th training session	5th training session
Intervention	+	+	+	+	+
Motivation to be active	+				
Approach tendencies	+				
Physical health	+				
Mental health	+				

WEEK 3	+1	+2	+3	+4	+5
Visit	1st training session	2nd training session	3th training session	4th training session	5th training session
Intervention	+	+	+	+	+
Motivation to be active	+				
Approach tendencies	+				
Physical health	+				
Mental health	+				

For peer review only - http://bmjopen.bmj.com/site/about/guidelines.xhtml

Appendix 6: The flow chart of the study design

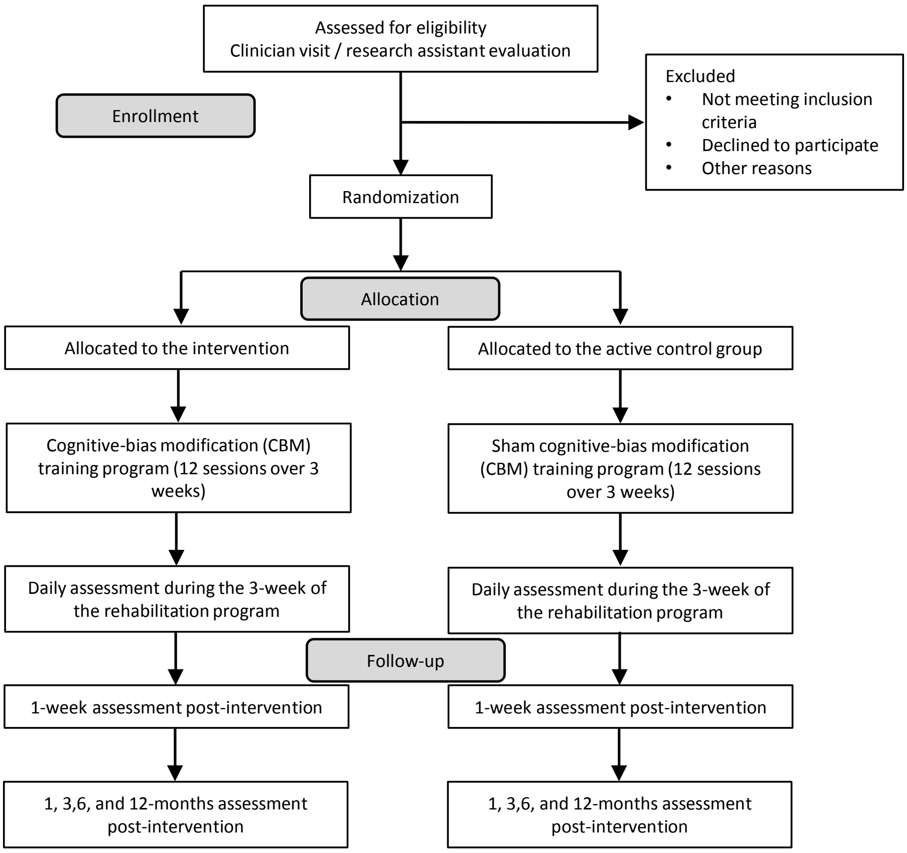

For peer review only - http://bmjopen.bmj.com/site/about/guidelines.xhtml

BMJ Open

A cognitive-bias modification intervention to improve physical activity in patients following a rehabilitation program: protocol for the randomized controlled IMPACT trial

Journal:	*BMJ Open*
Manuscript ID	bmjopen-2021-053845.R1
Article Type:	Protocol
Date Submitted by the Author:	04-Aug-2021
Complete List of Authors:	Cheval, Boris; University of Geneva; University of Geneva, Department of Psychology<br>Finckh, Axel; Geneva University Hospitals, Department of Medicine<br>Maltagliati, Silvio; University Grenoble Alpes<br>Fessler, Layan; University Grenoble-Alpes<br>Cullati, Stéphane ; University of Fribourg,<br>Sander, David; University of Geneva; University of Geneva, Department of Psychology<br>Friese, Malte; Saarland University<br>Wiers, Reinout W.; Univ Amsterdam<br>Boisgontier, Matthieu P.; Faculty of Health Sciences University of Ottawa; Bruyère Research Institute<br>Courvoisier, Delphine; Geneva University Hospitals, Division of clinical epidemiology<br>Luthy, C; Division of General Medical Rehabilitation, Geneva University Hospitals & University of Geneva
**Primary Subject Heading**:	Sports and exercise medicine
Secondary Subject Heading:	Rehabilitation medicine
Keywords:	SPORTS MEDICINE, Quality in health care < HEALTH SERVICES ADMINISTRATION & MANAGEMENT, REHABILITATION MEDICINE

SCHOLARONE™
Manuscripts

I, the Submitting Author has the right to grant and does grant on behalf of all authors of the Work (as defined in the below author licence), an exclusive licence and/or a non-exclusive licence for contributions from authors who are: i) UK Crown employees; ii) where BMJ has agreed a CC-BY licence shall apply, and/or iii) in accordance with the terms applicable for US Federal Government officers or employees acting as part of their official duties; on a worldwide, perpetual, irrevocable, royalty-free basis to BMJ Publishing Group Ltd ("BMJ") its licensees and where the relevant Journal is co-owned by BMJ to the co-owners of the Journal, to publish the Work in this journal and any other BMJ products and to exploit all rights, as set out in our licence.

The Submitting Author accepts and understands that any supply made under these terms is made by BMJ to the Submitting Author unless you are acting as an employee on behalf of your employer or a postgraduate student of an affiliated institution which is paying any applicable article publishing charge ("APC") for Open Access articles. Where the Submitting Author wishes to make the Work available on an Open Access basis (and intends to pay the relevant APC), the terms of reuse of such Open Access shall be governed by a Creative Commons licence – details of these licences and which Creative Commons licence will apply to this Work are set out in our licence referred to above.

Other than as permitted in any relevant BMJ Author's Self Archiving Policies, I confirm this Work has not been accepted for publication elsewhere, is not being considered for publication elsewhere and does not duplicate material already published. I confirm all authors consent to publication of this Work and authorise the granting of this licence.

For peer review only

A cognitive-bias modification intervention to improve physical activity in patients following a rehabilitation program: protocol for the randomized controlled IMPACT trial

Boris Cheval[1,2], Axel Finckh[3], Silvio Maltagliati[4], Layan Fessler[4], Stéphane Cullati[5,6], David Sander[1,2] Malte Friese[7], Reinout W. Wiers[8], Matthieu P. Boisgontier[9,10], Delphine Courvoisier[11], Christophe Luthy[12]

[1]Swiss Center for Affective Sciences, University of Geneva, Geneva, Switzerland

[2]Laboratory for the Study of Emotion Elicitation and Expression (E3Lab), Department of Psychology, University of Geneva, Geneva, Switzerland

[3]Division of Rheumatology, Department of Medicine, Geneva University Hospitals, Geneva, Switzerland

[4]Univ. Grenoble Alpes, SENS laboratory, Grenoble, France

[5]Population Health Laboratory, University of Fribourg, Switzerland.

[6]Department of Readaptation and Geriatrics, University of Geneva, Switzerland.

[7]Department of Psychology, Saarland University, Germany.

[8]Department of Psychology and center for Urban Mental Health, University of Amsterdam, The Netherlands.

[9]School of Rehabilitation Sciences, Faculty of Health Sciences, University of Ottawa, Ottawa, ON, Canada

[10]Bruyère Research Institute, Ottawa, ON, Canada

[11]Quality of Care Unit, Geneva University Hospitals, Geneva, Switzerland

[12]Division of General Medical Rehabilitation, University Hospitals of Geneva (HUG), Switzerland

For peer review only - http://bmjopen.bmj.com/site/about/guidelines.xhtml

Corresponding author: Campus, Biotech, Chemin des mines 9, 1202, Genève, Switzerland; boris.cheval@unige.ch; @ChevalBoris (B. Cheval).

Word count: 4800

Tables/Figures: 5

For peer review only

For peer review only - http://bmjopen.bmj.com/site/about/guidelines.xhtml

Abstract

Introduction. Being physically active is associated with a wide range of health benefits in patients. However, many patients do not engage in the recommended levels of physical activity (PA). To date, interventions promoting PA in patients mainly rely on providing knowledge about the benefits associated with PA to develop their motivation to be active. Yet, these interventions focusing on changing patients' conscious goals have proven to be rather ineffective in changing behaviors. Recent research on automatic factors (e.g., automatic approach tendencies) may provide additional targets for interventions. However, the implementation and evaluation of intervention designed to change these automatic bases of PA are rare. Consequently, little is known about whether and how interventions that target automatically activated processes toward PA can be effective in changing PA behaviors. The Improving physical activity (IMPACT) trial proposes to fill this knowledge gap by investigating the effect of a cognitive-bias modification intervention aiming to modify the automatic approach toward exercise-related stimuli on PA among patients.

Methods and analysis. The IMPACT trial is a single-center, placebo (sham controlled), triple-blinded, phase 3 randomized controlled trial that will recruit 308 patients enrolled in a rehabilitation program in the Division of General Medical Rehabilitation at the University Hospital of Geneva (Switzerland) and intends to follow them up for up to one year after intervention. Immediately after starting a rehabilitation program, patients will be randomized (1:1 ratio) to receive either the cognitive-bias modification intervention consisting of a 12-session training program performed over three weeks or a control condition (placebo). The cognitive-bias modification intervention aims to improve PA levels through a change in automatic approach tendencies toward PA and sedentary behaviors. The primary outcome is accelerometer-based PA. Secondary outcomes are related to changes in (1) automatic approach tendencies and self-reported motivation to be active, (2) physical health and (3) mental health. Sedentary behaviors and self-reported PA will also be examined. The main timepoint of the analysis will be the week after the end of the intervention. These outcomes will also be assessed during the rehabilitation program, as well as one, three, six, and 12 months after the intervention for secondary analyses.

Ethics and dissemination. The study will be conducted in accordance with the Declaration of Helsinki. This trial was approved by the Ethics Committee of Geneva Canton, Switzerland (reference number: CCER2019-02257). All participants will give an informed consent to participate in the study. Results will be published in relevant scientific journals and be disseminated in international conferences.

For peer review only - http://bmjopen.bmj.com/site/about/guidelines.xhtml

Strengths and limitations of this study

- The randomized controlled IMPACT trial will test the effects of an intervention based on cognitive-bias modification (CBM) to improve physical activity among patients following a rehabilitation program.

- Physical activity, sedentary behaviors, physical health, and mental health will be measured at multiple time points over one year.

- The findings from this well-powered study will provide evidence-based recommendations for clinical interventions aiming to promote physical activity among patients in rehabilitation.

- The reliance of a single center trial and the selection bias due to loss-to-follow-up and the volunteer participation are keys limitations that may reduce our ability to generalize the results to other populations.

For peer review only

Introduction

The health benefits of physical activity (PA) are well established and extensive. PA can reduce rates of cardiovascular diseases,[1] cancers,[2] hypertension,[3] diabetes,[4] obesity,[5] depression,[6] and all-cause mortality,[7] even more effectively than medication.[8] PA is safe and beneficial for almost everyone, while the risk of harm from moderate PA is small.[8 9] A recent systematic review and meta-analysis suggests that any PA, irrespective of the intensity, is beneficial for health.[7] In patients suffering from chronic diseases, increased PA is associated with reduced hospital admissions, decrease in pain, greater quality of life and mental health, and improvement in physical function.[8 10-13] These myriads of benefits even led the Academy of Medical Sciences to consider PA as a miracle cure.[14] Nevertheless, patients, similarly to the general population, remain largely physically inactive.[15-17]

Healthcare professionals are uniquely placed to promote PA among patients. Today, interventions aiming to enhance PA in patients largely relies on providing rational information about the benefits associated with PA. For example, a practical guide to help clinicians discussing about PA within a consultation has been recently proposed.[8] In this guide, clinicians are encouraged to rationally address patients' concerns about PA, to explain that there are more benefits to become active than to remain sedentary, to set an achievable goal, to identify barriers to be overcome, and finally to set a plan. This type of intervention guide is grounded in the dominant social-cognitive theories,[18] which contend that goals are proximal determinants of behaviors.[19 20] From these perspectives, changing patients' conscious goals should lead to substantial changes in their behaviors.[21 22] While these types interventions have proven to be effective to change PA behaviors to some extent,[23] meta-analyses also indicate that these approaches are more effective in changing intentions than in changing actual behavior.[24] Thus, developing additional interventions targeting alternative mechanisms is needed.

Recent research focusing on automatic mechanisms may provide additional targets for interventions.[25-29] For example, studies showed that in physically active individuals stimuli associated with PA attract attention,[30-33] trigger positive affective reactions,[34-37] and activate approach tendencies toward PA.[38-41] These automatically activated processes are thought to facilitate the translation of conscious goals into actual PA behaviors. Importantly, these automatic reactions predict PA behaviors above and beyond self-reported measures, such as the intention to be physically active[39], and are stronger predictors of spontaneous and unplanned actions that often consist of light-intensity physical activities.[42] As such, from this perspective,

For peer review only - http://bmjopen.bmj.com/site/about/guidelines.xhtml

physical inactivity is thought to also result from an imbalance between a strong motivation to be physically active, but weak automatic approach tendencies toward PA. Crucially, this imbalance between automatic and reflective processes may be particularly pronounced in patients, whose automatic reactions toward PA may be negatively biased by the fear, pain and discomfort felt during some exercises.[43] Thus, in comparison with the general population, patients may demonstrate more negative automatic reactions toward PA, including, for example, stronger negative affective reactions and weaker approach tendencies toward PA. One practical implication of these findings is that interventions designed to promote PA in patients might particularly benefit from directly targeting automatically activated processes toward PA.

What kinds of interventions can target automatically activated processes? New types of interventions have been developed to directly target these automatic reactions toward a given health behavior.[44 45] For example, in alcohol addiction, studies have used a cognitive-bias modification (CBM) intervention aimed at retraining automatic approach reactions toward alcohol using a computerized task.[46] In a CBM intervention, patients were repeatedly asked to push a joystick when exposed to alcohol-related pictures, simulating an avoidance movement. Specifically, in this computerized-based task, participants were asked to push or pull a joystick in response to the format of the pictures. For example, they were instructed to make a pushing movement when the picture presented on the screen was in the landscape format (i.e., avoidance), and to make a pulling movement when the picture was in the portrait format (i.e., approach). To ensure congruence with the participant's actions on the joystick, the picture became smaller when the participant pushed the joystick, and it became larger when the participant pulled the joystick. Participants received training in which they had to push the joystick away in response to pictures of alcohol (i.e., all alcohol pictures were presented in the push format) and to pull the joystick toward them in response to non-alcohol pictures (i.e., all non-alcohol pictures were presented in the pull format). Two large studies conducted in patients showed that adding a CBM intervention to a regular cognitive-behavior treatment yielded a beneficial effect on the relapse rates one year after treatment discharge, with a reduction of 9%,[47] 13%,[46] and 12%,[48] which could be attributed to changes in approach tendencies.[47 49] These interventions have also proven to be useful in impacting cigarette smoking,[50] social anxiety,[51] or eating behaviors.[52-54] Yet, it should be noted, the clinical effectiveness of CBM interventions has been criticized,[55 56] especially for anxiety and depression-related outcomes.[57-60]

For peer review only - http://bmjopen.bmj.com/site/about/guidelines.xhtml

To the best of our knowledge, however, only a handful set of studies have been conducted to target automatic processes toward physical activity.[61-64] Crucially, only one study has been conducted to examine the effect of a brief CBM intervention targeting approach-avoidance tendencies on an exercise task in a sample of healthy young adults.[64] Specifically, using a manikin task,[42 65] a variant of the approach-avoidance joystick task, participants were explicitly trained to repeatedly approach a manikin toward pictures depicting PA and to avoid pictures depicting sedentary behaviors, by pressing keys on the keyboard. Results revealed that participants spent more time exercising during a laboratory exercise task of moderate intensity (i.e., doing squat), in comparison with control groups either trained to approach stimuli depicting sedentary behaviors and avoid stimuli depicting PA (i.e., reverse contingencies) or to approach and avoid stimuli depicting PA and sedentary behaviors equally often (sham controlled). These findings suggest that a single and brief CBM session targeting automatic approach tendencies toward PA and sedentary behaviors can have beneficial effect on laboratory-based PA behaviors. However, this study has at least two important limitations. First, it is unclear if and to what extent the PA behavior performed in the laboratory extends to behaviors performed in everyday life, thereby preventing the possibility to determine whether CMB manipulations can be effective in changing daily-life behaviors. Second, the study was conducted on a sample of rather physically active college students. As such the potential beneficial effect of adding a CBM intervention to a regular treatment in patients, a population which may particularly benefit from such manipulation, remains unknown.

Objectives

In sum, while recent research highlights the importance of targeting automatically activated processes related to PA, the effectiveness of interventions designed to change these presumed automatic bases of PA behaviors has been largely overlooked. Consequently, little is known about whether and how interventions that target automatically activated processes toward PA can be effective in changing behaviors. The primary objective of the IMPACT trial is to investigate the effectiveness of a CBM intervention targeting automatic approach tendencies toward exercise-related stimuli on PA patients in a rehabilitation program. This trial will be performed using a placebo, triple-blinded, phase 3 randomized controlled trial. The secondary objectives are to evaluate the effect of this CBM intervention on changes in (1) automatic approach tendencies and self-reported motivation to be active, (2) physical health and (3) mental health. We hypothesize that the CBM intervention will be associated with higher levels of PA (pre vs. 1-week post intervention) (H1). Moreover, we hypothesize that the CBM

For peer review only

intervention will increase automatic approach tendencies toward PA (H2a), but will decrease automatic approach tendencies toward sedentary behaviors (H2b). Finally, we predict that the CBM intervention will improve patients' physical and mental health (H3). All these hypotheses will also be tested during the rehabilitation program as well as one, three, six, and 12 months after the intervention (secondary analyses).

Methods and data analysis

Study design

The IMPACT trial is a single-center, placebo (sham controlled), triple-blinded, phase 3 randomized controlled trial. The trial will start (First-Participant-In) January 2022 in the ward 3DK of the Division of General Medical Rehabilitation (University Hospitals of Geneva; Switzerland) and will finish (Last-Participant-Out) in January 2024. The ward 3DK admits and manages patients for treatments or diagnostics evaluations, especially after being in acute care for several reasons, such as serious infections, cancer, heart failure, or post-surgery follow-up treatments. This ward offers multidisciplinary treatment in rehabilitation (e.g., physiotherapists, occupational therapists, nutritionists) and does not focus on improving PA engagement. In other words, within the usual care, there is not any content specifically devoted to improve patients' PA level. Eligible patients will be randomly assigned to either the CBM intervention or the active control condition (placebo) in a 1:1 ratio. The current study follows the Standard Protocol Items: Recommendations for Interventional Trials (SPIRIT) statement.[66] The clinical trial was registered at the German clinical trials register (reference number: DRKS00023617).

Eligibility criteria

The eligibility criteria are listed in Box 1. Participants fulfilling all the inclusion criteria are eligible for the study. The presence of the exclusion criterion will lead to the exclusion of the participant.

Box 1. Inclusion and exclusion criteria

Inclusion criteria

- Patients treated in the ward 3DK of the Division of General Medical Rehabilitation
- Aged 18 years or older
- Can comply with the study protocol
- Able to provide a written consent of participation in the trial.

For peer review only

For peer review only - http://bmjopen.bmj.com/site/about/guidelines.xhtml

Exclusion criteria
• Contraindication to PA in the view of the health status

Decision to include/exclude a participant

The decision to include/exclude a participant from this study will be jointly decided by the chief medical officer and the research assistant.

Participant screening, recruitment, and consent

All patients starting rehabilitation program in the ward 3DK of the Division of General Medical Rehabilitation, University Hospital of Geneva, Switzerland (from January 2022 to January 2024) will be approached during the first consultation with the chief medical officer and will receive an information sheet explaining the main objective of the IMPACT trial. The investigators will explain to each participant the nature of the study, its purpose, the procedures involved, the expected duration, the potential risks and benefits and any discomfort it may entail. Each participant will be informed that the participation in the study is voluntary and that he or she may withdraw from the study at any time and that withdrawal of consent will not affect his or her subsequent medical assistance and treatment. The participant will be informed that his or her medical records may be examined by authorized individuals other than their treating physician. All participants will be provided a participant information sheet and a consent form describing the study and providing sufficient information for participants to make an informed decision about their participation in the study. Participants will have time to carefully read the documents and can give their responses up to 24 hours after having received the documents. The formal consent of a participant, using the approved consent form, will be obtained before the participant is submitted to any study procedure. Participants will then complete a first questionnaire assessing the exclusion and inclusion criteria, as well as other screening measures. All the questionnaires will be assessed electronically using REDCap software. Finally, patients' expectations regarding the effects of the intervention will be assessed.[67] Table 1 provides an overview of all the baseline screening measures available. The study patient flow chart is provided in Figure 1.

Sample size

For power calculation, our intervention implements a between-subject design and random-effects statistical models (i.e., t-tests). Considering a conservative medium effect size (Cohen's d = 0.45), a sample size calculation indicates that a minimum of 220 patients (110 per arm)

For peer review only - http://bmjopen.bmj.com/site/about/guidelines.xhtml

would be needed to demonstrate efficacy of the intervention on the device-based PA during the week following the intervention, with a probability of committing a type I error < 5% and a probability of committing a type II error < 10%. We expect a loss to follow-up of 10 to 20% at one week after the intervention, and a loss of 30 to 40% over one year. Thus, a minimum of 308 patients will be recruited.

Feasibility

The ward 3DK of the Division of General Medical Rehabilitation has 24 beds and treats on average 40 patients per month. Based on the chief medical officer's experiences and a first presentation of the study to the patients treated in this unit, we expect that 3 patients out of 5 will not agree (for various reasons) to participate in the study, thereby leading to a total of about 24 participants recruited per month. Consequently, we should be able to collect the target sample size in approximately 12-14 months. The average duration of participants hospitalization in the ward 3DK is about three weeks. As such, though this duration can vary between patients (i.e., some patients only stay a few days), this duration allows for the implementation of the whole intervention (i.e., 12-session training program performed over three weeks). Of note, participants who will not complete all the training sessions will still be included in the analysis. Sensibility analyses will be conducted to examine whether the number of completed sessions influence the effects of the intervention. To accelerate and facilitate knowledge dissemination, all articles will be preprinted, and data and code shared on public repositories.

Patients adherence to the IMPACT trial

Patients adherence to the training program (i.e., if the planned training session is completed or not, and why in case of no completion) and to the other measures are documented in an electronic Case Report Form (eCRF) powered by REDCap.[68] To promote patient retention and complete follow-up (i.e., one, three, six and 12 months after the end of the intervention), participants will be contacted by phone by a research assistant two weeks before the follow-up measurement. If they do not answer, they will receive up to two additional phone calls this week. If they do not answer, this procedure will be repeated the following week. If they still do not answer, this time of measurement will be considered as missing. Patients with missing data at a given wave, will be contacted for the following waves through the above-mentioned procedure.

For peer review only

Patients who did not answer a given time of measurement, will still be contacted to participate in the following timepoints.

Interventions

All newly admitted patients will attend a meeting organized in the unit. The objective of this meeting will be to present and illustrate the health benefits of PA. Consistent with the recent practical guide to help healthcare professionals promoting PA to patients,[8] research assistants will follow the "Ask-Assess-Advise" structure for discussing PA behavior change in the consultation. Patients will also receive a watch tracking (i.e., polar) during the rehabilitation period and giving personalized feedback on their PA and sedentary behaviors. This procedure aims at increasing their self-reported motivation to be active, thereby allowing to examine the additional effects of the CBM intervention.

Intervention group: Training program of 12 sessions over 3 weeks (i.e., 4 sessions by week on average) using an adapted version of the Visual-Approach/Avoidance-by-the-Self Task (VAAST),[69] a task that have shown to produce large and replicable effects, compared with the manikin task. Specifically, patients will be asked to react to the format (i.e., portrait vs. landscape format) of the pictures depicting PA and of sedentary behaviors by pressing twice the "move forward" or "move backward" key press to approach or avoid the pictures, respectively. Participants will be instructed to approach the picture when it appears in a portrait format, and to avoid it when the picture appears in a landscape format (the rule will be counterbalanced between participants). Of note, unlike the previous study that relied on an explicit instruction task (i.e., participants were asked to respond to the content of the pictures),[64] the current study uses an irrelevant feature task (i.e., participants were asked to respond to the format of the pictures). This irrelevant feature task allows a training without explicit instruction. Congruent with the patient's approach or avoidance response, the whole visual environment will zoom in on the picture to simulate an approach movement and zoom out to simulate an avoidance movement. A change by 10% after each key press will be used to give the impression to walk forward or backward as a consequence of the responses. Participants in the intervention group will receive training in which 90% of pictures depicting PA will be presented in the approach format (and 10% in the avoidance format), and 90% pictures depicting sedentary behaviors will be presented in the avoidance format (and 10% in the approach format). This 90/10 split aims to increase the patients blinding to the condition in which they will be assigned. Each training session will consist of 144 trials for a total duration of approximately 10 minutes.

For peer review only - http://bmjopen.bmj.com/site/about/guidelines.xhtml
For peer review only

At the first session and at the beginning of each week, the training session will be preceded by 96 assessment trials in which the contingency of approaching or avoiding PA or sedentary behaviors will be 50%. Assessment trials will allow to measure patients' automatic approach-avoidance tendencies toward PA and sedentary behaviors (see Figure 2).

Comparator group: Patients in the comparator group (placebo; sham controlled) will not be trained to approach PA and to avoid sedentary behaviors. Specifically, the retraining sessions will also consist of 144 trials, but the task will require an equal number of approach and avoidance responses to both stimuli depicting PA and sedentary behaviors (see Figure 2). The use of a placebo was chosen to ensure that the potential effects of the experimental condition will be attributable to the content of the training program (i.e., learn to systematically approach PA-related stimuli and avoid sedentary behaviors-related stimuli) rather than because of a simple exposition effect (i.e., the fact to be exposed longer to contents related to physical activity and sedentary behaviors).

Stimuli: Stimuli representing PA and sedentary behaviors will be created using the Unity software ®. A set of 195 pictures including 14 avatars (50% women) in either active (walking and running) and inactive posture (sit on a cubicle) will be tested in a pilot study to identify the 48 pictures the most associated with "movement and physically active behaviors" and the 48 pictures the most associated with "rest and physically inactive behaviors" using two visual analogic scales (VAS 1; *"please indicate how this image is, in your opinion, associated with a behavior that requires: 0 = no physical exertion at all, 100 = a lot of physical exertion*"; VAS 2; *"Please indicate how closely this image is associated with: 0 = resting, sedentary behavior, 100 = movement, very active behavior").* The credibility of the pictures will also be tested ("*how realistic do you think this person's behavior is? Realistic meaning that the images may resemble to a real-life behavior*"; on a VAS from 0 = behavior not at all realistic; 100 = behavior very realistic) and for agreeableness *("how pleasant/sympathetic do you find the person in this image? For example, would you like to talk to her/him"*; from 0 = very unpleasant/antipathetic, 100 = very pleasant/sympathetic). The aim of this pilot study was twofold. First, to ensure that the selected pictures reflect the concept of interest (i.e., movement and physical activity vs. rest and physical inactivity). Second, to check that the selected pictures were equivalent in term of credibility and agreeableness across categories (i.e., movement vs. rest). Pictures will be built to match for color, brightness, and visual complexity. To examine the generalization of training effects,[70] in both the intervention and comparator group, only half of the pictures used in the

For peer review only - http://bmjopen.bmj.com/site/about/guidelines.xhtml

assessment phase will be included on the training phase (the selected pictures will be counterbalanced across participants).

Randomization and blinding

The research assistants and the participants will be blinded to the allocation of the groups. At the end of the trial, the success of the participant blinding will be examined by asking the participants to guess in what group there were, including a percentage of certainty. Moreover, the success of research assistants blinding will be examined by asking each research assistant if they were able to detect the group (comparator vs. intervention) when they conducted the data collection.

The randomization will be generated on a computer and will be performed using permuted blocks (size = 8). To ensure that the research team will be blinded to the randomization, an independent co-worker will carry out the randomization. The patient's identification number will be used to determine the sequence of randomization. Patients will be randomized in a 1:1 ratio between the intervention and active control condition. Unblinding is not planned during the trial as we do not see any reasons that would require either the patients or the researchers to know the group in which the patients were allocated. However, if requested by the patients, unblinding is permissible at the end of the trial.

Outcomes

Primary outcomes

The primary outcome will be the accelerometer-based time spent in PA. Following recommendations in patients,[71] a three-axis accelerometer (Actigraph GT3X+; Pensacola, USA) will be used to assess PA. Patients will be given the accelerometer and related indications during the first training session. They will be asked to wear the accelerometer for the full week and to return during the next appointment. They will be instructed on how to wear the device (i.e., over the right hip, affixed to an elastic belt, preferably worn under their waistbands). Currently, the waist-mounted Actigraph is the most used device to objectively measure physical activity.[72] One-minute epochs will be used for data analyses and non-wear time will be defined as ≥ 59 consecutive minutes of zero counts. Daily data will be included if the wear time is ≥ 10 waking hours per day [73]. Data will be included if ≥ 4 days met the aforementioned conditions.[74] The times spent in light, moderate, and vigorous PA will be determined through previously

For peer review only - http://bmjopen.bmj.com/site/about/guidelines.xhtml

validated cut points,[75] in bouts lasting at least 10 min, and will be used as an outcome. Then, in the week following the rehabilitation period, participants will be asked to wear the accelerometer for one week. Of note, because the duration of the rehabilitation period may strongly vary between patients, it is possible that some patients will be still in the hospital after three weeks, while other will leave the service sooner (e.g., at two weeks). As such, to account for this feature and to allow comparisons between patients, the accelerometer will be scheduled to start on the Monday following their discharge from the rehabilitation unit, regardless the lengths of stay in the ward. Finally, participants will be asked to wear the accelerometer for one week at one, three, six, and 12 months post-intervention.

Secondary outcomes

The secondary outcomes will be the changes in (1) automatic approach tendencies and self-reported motivation to be active, (2) physical health, and (3) mental health. Sedentary behaviors and self-reported PA will also be examined. Table 2 provides an overview of all the outcomes measures and Table 3 provides the schedule of assessment.

Data analysis

Primary analyses

Statistical analyses will be performed according to the intention-to-treat (ITT) principle and will abide by the Consolidated Standards of Reporting Trials (CONSORT) guidelines. Analysis will be conducted in a blinded way. We will use mean, standard deviation (SD), median, and range values to summarize the continuous data. The primary outcome will be analyzed using multiple linear regressions. Specifically, to test H1, we will test whether the patients' PA level during the week after the end of the intervention will be higher in the intervention group relative to the comparator group, after adjustment for covariates (i.e., age, sex, and indicators of the medical evaluation during the screening assessment). To test H2a and H2b, we will test whether patients' automatic approach tendencies toward PA will be higher and patients' automatic approach tendencies toward sedentary behaviors will be lower in the intervention group relative to the comparator group, after adjustment for covariates. Finally, to test H3, we will test whether patients' physical and mental health during the week after the end of the intervention will be higher in the intervention group relative to the comparator group, after adjustment for covariates. Moderator analyses (i.e., for motivation to change, usual level of PA, personality, expectations for improvement) will be conducted.

For peer review only - http://bmjopen.bmj.com/site/about/guidelines.xhtml

For peer review only

Secondary analyses

The aforementioned models will be tested at one, three, six, and 12 months after the intervention. Moreover, to examine the effect of the intervention during the rehabilitation period, mixed effects models will be used. These models account for the nested structure of the data (i.e., multiple observations within a single participant), thereby providing accurate parameter estimates with acceptable Type I error rates.[76] Moreover, these models do not require an equal number of observations across participants, thereby allowing participants with missing observations to be included in the analyses without the need to impute those missing data. To formally examine the effect of the intervention on the evolution of PA within the rehabilitation period, models will include interaction terms between conditions (intervention group vs. comparator group) and number of days within the rehabilitation program (linear and quadratic). The number of days should be relatively equal between patients (about 21 days) but may differ to some extent (some patients can leave earlier or other later than 21 days). A statistically significant interaction will indicate that the rate of PA change throughout the rehabilitation program would be different across the conditions. The quadratic effect of the number of days will be included to account for potential non-linear change of PA across the rehabilitation period. This will allow, for instance, to model the possibility that the effect of the intervention will take some sessions before becoming effective or that no additional effect could be hoped after a certain number of sessions. The continuous secondary outcomes will be treated in the similar way to the primary outcome. All analyses will be conducted using R software®. Any deviation from the original statistical plan will be described and justified in the final trial report.

Data security, management, and monitoring

Project data will be handled with uttermost discretion and will be only accessible to authorized personnel who require the data to fulfill their duties within the scope of the research project. On the online Case Report Forms (CRFs) and other specific documents, participants are only identified by a unique participant number. The online CRF will be created using Redcap.

Data recording: The dataset will be accompanied by a README file, which will describe the directory hierarchy and file naming convention. The directory will contain an INFO file describing the experimental protocol used in that experiment. This INFO file will also record any deviation from the protocol and other useful contextual information. This procedure should allow the data to be easily understood by other researchers and should support future reuse of the data. Metadata will be created to provide contextual information required to interpret data.

For peer review only - http://bmjopen.bmj.com/site/about/guidelines.xhtml

This metadata file will be created in accordance with the Data Documentation Initiative (DDI). In particular, the metadata file will include short unique identifier, the name of the author(s), the content, the date of creation, the locations, the reason why the data was generated, and how the data was created. The codebook will explicitly indicate the name, explanations, and the modalities of the different variables measured in the experiment. In addition, it will include information on the study design and contain all information necessary for another analyst to use the data accurately.

Data anonymization: Individual participant information collected during the study is considered confidential and disclosure to third parties is prohibited. Subject confidentiality will be ensured by utilizing subject identification code numbers to correspond to treatment data in the computer files. Only a minority of personnel (i.e., the principal investigator and chief medical officer) will have access to the data in a non-coded form.

Data storage: Participant data on a secure database in accordance with the General Data Protection Regulations (2018). Three copies of the data will be stored. First, original data will be stored on the principal investigator's computer, which will be backed up daily, and protected by a password. Additionally, data will be stored on a secure server hosted by the University of Geneva. Finally, data will be stored on an external device at a different location and be protected by a password. The original notebook will be stored in the principal investigator's laboratory. Local version of the data for statistical analysis will remain on a University computer, and be password protected. Each person who collected the data will have the responsibility to annotate their data within the metadata. Nevertheless, the principal investigator will have the responsibility to weekly check that the data is properly processed, documented, and stored. All study data will be archived for a minimum of 10 years beyond the end of the randomized controlled trial.

Trial monitoring: The PI will organize a proper training of all involved study personnel to ensure that the study will be conducted according to the protocol. Research assistants should understand the detailed contents of the protocol before starting the data collection. For quality assurance the Ethics Committee may visit the research sites. Direct access to the source data and all project-related files and documents must be granted on such occasions. The principal investigator or any other competent authority may terminate the study prematurely according to the following circumstances: ethical concerns, insufficient participants recruitment, early

For peer review only

For peer review only - http://bmjopen.bmj.com/site/about/guidelines.xhtml

evidence of harm or benefit of the experimental intervention through the interim analysis planned at six months after the start of the trial. Although no serious adverse event resulting from the intervention is expected, all potential adverse events will be documented within the eCRF.

Patient and public involvement in the trial design

No patient or public was involved in the present study.

Ethics and dissemination

The study was approved by the Ethics Committee of Geneva Canton, Switzerland (reference number: CCER2019-02257). All participants will give an informed consent to participate in the study.

Results will be published in relevant scientific journals and be disseminated in international conferences. Anonymity of the participants will be guaranteed when presenting the data at scientific meetings or publishing them in scientific journals. Individual participant information collected during the study is considered confidential and disclosure to third parties is prohibited.

Data sharing and reuse: Datasets and metadata from this trial will be deposited in ZENODO (a generic and free repository based at CERN, Geneva), and made public at the time of publication. Data in the repository will be stored in accordance with funder and university data policies. Particularly, original datasets, original software script and code, and original raw data will be deposited. However, as stressed above, personal data will be anonymized before diffusion.

Discussion

PA is associated with a wide range of health benefits,[1-7] but patients, similarly to the general population, remain largely physically inactive. Promoting PA to patients is thus urgently warranted, and healthcare professionals are uniquely placed to do so.[8] To date, interventions mainly rely on providing rational information to change patients' conscious goals and motivation to be active. Yet, these approaches are insufficient to substantially impact actual behaviors.[24] One explanation for this lack of effectiveness draws on recent observations suggesting that automatic reactions toward exercise-related stimuli are involved in the regulation of PA.[33 34 39 77 78] As such, developing interventions targeting both reflective (e.g.,

For peer review only

motivation) and automatic (e.g., approach tendencies) precursors of PA could be particularly effective. This protocol paper outlines the design of the IMPACT trial, the first placebo, triple-blinded, randomized controlled trial examining the effectiveness of a CBM intervention targeting automatic approach tendencies toward exercise-related stimuli on PA in patients in rehabilitation program remains. The IMPACT trial will focus on an accelerometer-based measure of PA as the primary outcome due to all the extensive benefits associated with being physically active. The secondary outcomes will allow examining other positive-side effects of the intervention on physical and mental health.

Strengths and limitations

The IMPACT randomized controlled trial has several strengths. Firstly, it is the first randomized controlled trial investigating the beneficial effect of an easy deliverable CBM intervention promoting physical activity among patients enrolled in a multidisciplinary rehabilitation program. Secondly, this CBM intervention is anchored within the dual-process models of behavior, arguing that automatic reactions toward physical activity represent additional targets for interventions. Accordingly, this trial will examine for the first time the efficacy of these new types of interventions, which directly targets the automatic precursors of physical activity behavior. Thirdly, we relied on an accelerometer-based measure of physical activity, which guarantee the validity and reliability of our primary outcome. Finally, in addition to physical activity behavior, we will collect data on physical and mental health at multiple time points over one year. However, potential limitations should be noted. The first limitation is related to the fact that the trial is based on a single center, which will limit the generalization of the results to other centers. Second, because of the longitudinal design (i.e., the main time point for the main analysis is assessed four weeks after the start of the intervention and additional time point for secondary analyses are assessed one, three, six, and 12 months after the start of the intervention), we cannot exclude a selection bias due to attrition. Likewise, as participation in our study is voluntary, it may favor the selection of patients with a higher health status or the most motivated to engage in PA. These features are key limitations that may reduce our ability to generalize the results to other populations. Third, to reduce patients' burden, the measure of physical and mental health is based on a single or few items, which may reduce the reliability and validity of these secondary outcomes' measurement. Finally, the rehabilitation program in the Division of General Medical Rehabilitation is a program receiving patients that have been in acute care for different reasons such as serious infections, cancer, heart or lung failure, or post-surgery follow-up treatments. Accordingly, the profiles of the

For peer review only - http://bmjopen.bmj.com/site/about/guidelines.xhtml

patients included in the trial may strongly differ from one patient to another. Therefore, although patients' profile (e.g., age, sex, or features of the medical evaluation) will be adjusted in the model, the diversity of those profiles may still produce a level of variability likely to influence the effects of the intervention.

Conclusion

In conclusion, PA is a key factor to improve the management of patients' diseases. Helping patients to become more active is likely to promote their recovery, their physical and mental health, as well as to reduce the development of other comorbidities. Targeting automatic reactions toward PA, which may be negatively biased in patients, is particularly innovative. Furthermore, this low cost and easily deliverable intervention could be rapidly implemented on a large scale to help patients become more physically active. The findings from this study will provide evidence-based conclusions for future interventions promoting PA in patients.

For peer review only

Contributors: B.C.: conceptualization, writing – original draft; A.F.: conceptualization, writing – review & editing; S.M.: Methodology – creation of the tasks, writing – review & editing; L.S.: writing – review & editing; S.C.: writing – review & editing; D.S.: writing – review & editing; M.F.: writing – review & editing; R.W.W.: writing – review & editing; M.P.B.: conceptualization, writing – original draft; D.C.: supervision, resources – provision on instrumentation, writing – review & editing; C.L.: supervision, resources – provision of study materials, writing – original draft

Funding: This work was supported by the Swiss National Science Foundation, grant number PZ00P1_180040. The purchase of protocol-related material (i.e., 30 Polar watches and accelerometers) is provided by the non-operating fund of the Division of General Medical Rehabilitation, University Hospital of Geneva, Switzerland. The computer equipment (3 laptops) will be made available by Division of General Medical Rehabilitation, University Hospital of Geneva. M.P.B. is supported by the Natural Sciences and Engineering Research Council of Canada (RGPIN-2021-03153) and by a grant from The Banting Research Foundation.

Competing interests: None declared

Participants consent: Not obtained

Ethics approval: Ethics Committee of the Geneva University Hospitals, Switzerland

Provenance and peer review: Not commissioned; peer reviewed for ethical and funding approval prior to submission.

Data availability statement: Datasets and metadata from this trial will be deposited in ZENODO (a generic and free repository based at CERN, Geneva) for the duration of 20 years, and made public at the time of publication.

For peer review only

References

1. Wahid A, Manek N, Nichols M, et al. Quantifying the association between physical activity and cardiovascular disease and diabetes: a systematic review and meta-analysis. *J Am Heart Assoc* 2016;5:e002495.
2. Moore SC, Lee I-M, Weiderpass E, et al. Association of leisure-time physical activity with risk of 26 types of cancer in 1.44 million adults. *JAMA Intern Med* 2016;176:816-25.
3. Liu X, Zhang D, Liu Y, et al. Dose–response association between physical activity and incident hypertension: a systematic review and meta-analysis of cohort studies. *Hypertension* 2017;69:813-20.
4. Aune D, Norat T, Leitzmann M, et al. Physical activity and the risk of type 2 diabetes: a systematic review and dose–response meta-analysis. *Eur J Epidemiol* 2015;30:529-42.
5. Bleich SN, Vercammen KA, Zatz LY, et al. Interventions to prevent global childhood overweight and obesity: a systematic review. *Lancet Diabetes Endocrinol* 2018;6:332-46.
6. Schuch F, Vancampfort D, Firth J, et al. Physical activity and sedentary behavior in people with major depressive disorder: a systematic review and meta-analysis. *J Affect Disord* 2017;210:139-50.
7. Ekelund U, Tarp J, Steene-Johannessen J, et al. Dose-response associations between accelerometry measured physical activity and sedentary time and all cause mortality: systematic review and harmonised meta-analysis. *BMJ* 2019;366:l4570.
8. Haseler C, Crooke R, Haseler T. Promoting physical activity to patients. *BMJ* 2019;366:l5230.
9. Godlee F. The miracle cure. *BMJ* 2019;366:l5605.
10. Anderson L, Oldridge N, Thompson DR, et al. Exercise-based cardiac rehabilitation for coronary heart disease: Cochrane systematic review and meta-analysis. *J Am Coll Cardiol* 2016;67:1-12.
11. Stevinson C, Lawlor DA, Fox KR. Exercise interventions for cancer patients: systematic review of controlled trials. *Cancer causes & control* 2004;15:1035-56.
12. Taylor RS, Brown A, Ebrahim S, et al. Exercise-based rehabilitation for patients with coronary heart disease: systematic review and meta-analysis of randomized controlled trials. *The American journal of medicine* 2004;116:682-92.
13. Zanuso S, Jimenez A, Pugliese G, et al. Exercise for the management of type 2 diabetes: a review of the evidence. *Acta Diabetol* 2010;47:15-22.
14. Exercise: the miracle cure and the role of the doctor in promoting it. https://www.aomrc.org.uk/wp-content/uploads/2016/05/Exercise_the_Miracle_Cure_0215.pdf. 2015
15. Johansen KL, Chertow GM, Ng AV, et al. Physical activity levels in patients on hemodialysis and healthy sedentary controls. *Kidney Int* 2000;57:2564-70.
16. Watz H, Waschki B, Meyer T, et al. Physical activity in patients with COPD. *Eur Respir J* 2009;33:262-72.
17. Jeong S-W, Kim S-H, Kang S-H, et al. Mortality reduction with physical activity in patients with and without cardiovascular disease. *Eur Heart J* 2019;40:3547-55.
18. Rhodes RE, Nigg CR. Advancing physical activity theory: a review and future directions. *Exerc Sport Sci Rev* 2011;39:113-19.
19. Bandura A. Self-efficacy: The exercise of control (1st edition). New York: Worth Publishers 1997.
20. Carver CS, Scheier MF. On the self-regulation of behavior. New York: Cambridge University Press 1998.

For peer review only

21. Sheeran P, Gollwitzer PM, Bargh JA. Nonconscious processes and health. *Health Psychol* 2013;32:460-73.
22. Brand R, Cheval B. Theories to explain exercise motivation and physical inactivity: Ways of expanding our current theoretical perspective. *Front Psychol* 2019;10:1147.
23. Hillsdon M, Foster C, Thorogood M. Interventions for promoting physical activity. *Cochrane Database Syst Rev* 2005;25:CD003180.
24. Rhodes RE, Dickau L. Experimental evidence for the intention–behavior relationship in the physical activity domain: a meta-analysis. *Health Psychol* 2012;31:724-27.
25. Cheval B, Radel R, Neva JL, et al. Behavioral and neural evidence of the rewarding value of exercise behaviors: a systematic review. *Sports Med* 2018;48:1389-404.
26. Brand R, Ekkekakis P. Affective–Reflective Theory of physical inactivity and exercise. *Ger J Exerc Sport Res* 2018;48:48-58.
27. Conroy DE, Berry TR. Automatic affective evaluations of physical activity. *Exerc Sport Sci Rev* 2017;45:230-37.
28. Cheval B, Sarrazin P, Boisgontier MP, et al. Temptations toward behaviors minimizing energetic costs (BMEC) automatically activate physical activity goals in successful exercisers. *Psychol Sport Exerc* 2017;30:110-17.
29. Cheval B, Boisgontier MP. The theory of effort minimization in physical activity. *Exerc Sport Sci Rev* 2021
30. Berry TR. Who's even interested in the exercise message? Attentional bias for exercise and sedentary-lifestyle related words. *J Sport Exerc Psychol* 2006;28:4-17.
31. Berry TR, Spence JC, Stolp SM. Attentional bias for exercise-related images. *Res Q Exerc Sport* 2011;82:302-9.
32. Calitri R, Lowe R, Eves FF, et al. Associations between visual attention, implicit and explicit attitude and behaviour for physical activity. *Psychol Health* 2009;24:1105-23.
33. Cheval B, Miller MW, Orsholits D, et al. Physically active individuals look for more: an eye-tracking study of attentional bias. *Psychophysiology* 2020;57:e13582.
34. Conroy DE, Hyde AL, Doerksen SE, et al. Implicit attitudes and explicit motivation prospectively predict physical activity. *Ann Behav Med* 2010;39:112-18.
35. Rebar AL, Ram N, Conroy DE. Using the EZ-diffusion model to score a single-category implicit association test of physical activity. *Psychol Sport Exerc* 2015;16:96-105.
36. Bluemke M, Brand R, Schweizer G, et al. Exercise might be good for me, but I don't feel good about it: do automatic associations predict exercise behavior? *J Sport Exerc Psychol* 2010;32:137-53.
37. Chevance G, Caudroit J, Romain AJ, et al. The adoption of physical activity and eating behaviors among persons with obesity and in the general population: the role of implicit attitudes within the Theory of Planned Behavior. *Psychol Health Med* 2017;22:319-24.
38. Hannan TE, Moffitt RL, Neumann DL, et al. Implicit approach–avoidance associations predict leisure-time exercise independently of explicit exercise motivation. *Sport Exerc Perform Psychol* 2019;8:210-22.
39. Cheval B, Sarrazin P, Isoard-Gautheur S, et al. Reflective and impulsive processes explain (in)effectiveness of messages promoting physical activity: a randomized controlled trial. *Health Psychol* 2015;34:10-19.
40. Cheval B, Sarrazin P, Isoard-Gautheur S, et al. How impulsivity shapes the interplay of impulsive and reflective processes involved in objective physical activity. *Pers Individ Dif* 2016;96:132-7.
41. Cheval B, Tipura E, Burra N, et al. Avoiding sedentary behaviors requires more cortical resources than avoiding physical activity: an EEG study. *Neuropsychologia* 2018;119:68-80.

For peer review only - http://bmjopen.bmj.com/site/about/guidelines.xhtml

42. Cheval B, Sarrazin P, Pelletier L. Impulsive approach tendencies towards physical activity and sedentary behaviors, but not reflective intentions, prospectively predict non-exercise activity thermogenesis. *Plos One* 2014;9:e115238.
43. Shelby RA, Somers TJ, Keefe FJ, et al. Domain specific self-efficacy mediates the impact of pain catastrophizing on pain and disability in overweight and obese osteoarthritis patients. *J Pain* 2008;9:912-19.
44. Friese M, Hofmann W, Wiers RW. On taming horses and strengthening riders: Recent developments in research on interventions to improve self-control in health behaviors. *Self Identity* 2011;10:336-51.
45. Marteau TM, Hollands GJ, Fletcher PC. Changing human behavior to prevent disease: the importance of targeting automatic processes. *Science* 2012;337:1492-95.
46. Wiers RW, Eberl C, Rinck M, et al. Retraining automatic action tendencies changes alcoholic patients' approach bias for alcohol and improves treatment outcome. *Psychol Sci* 2011;22:490-97.
47. Eberl C, Wiers RW, Pawelczack S, et al. Approach bias modification in alcohol dependence: do clinical effects replicate and for whom does it work best? *Dev Cogn Neurosci* 2013;4:38-51.
48. Manning V, Garfield JB, Staiger PK, et al. Effect of cognitive bias modification on early relapse among adults undergoing inpatient alcohol withdrawal treatment: a randomized clinical trial. *JAMA Psychiat* 2021;78:133-40.
49. Gladwin TE, Rinck M, Eberl C, et al. Mediation of cognitive bias modification for alcohol addiction via stimulus-specific alcohol avoidance association. *Alcohol Clin Exp Res* 2015;39:101-07.
50. Wittekind CE, Feist A, Schneider BC, et al. The approach-avoidance task as an online intervention in cigarette smoking: A pilot study. *J Behav Ther Exp* 2015;46:115-20.
51. Taylor CT, Amir N. Modifying automatic approach action tendencies in individuals with elevated social anxiety symptoms. *Behaviour research and therapy* 2012;50:529-36.
52. Fishbach A, Friedman RS, Kruglanski AW. Leading us not unto temptation: Momentary allurements elicit overriding goal activation. *J Pers Soc Psychol* 2003;84:296-309. doi: 10.1037/0022-3514.84.2.296
53. Fishbach A, Shah JY. Self-control in action: implicit dispositions toward goals and away from temptations. *J Pers Soc Psychol* 2006;90:820.
54. Aulbach MB, Knittle K, Haukkala A. Implicit process interventions in eating behaviour: a meta-analysis examining mediators and moderators. *Health Psychol Rev* 2019;13:179-208.
55. Field M, Duka T, Eastwood B, et al. Experimental manipulation of attentional biases in heavy drinkers: do the effects generalise? *Psychopharmacology* 2007;192:593-608.
56. Schoenmakers T, Wiers RW, Jones BT, et al. Attentional re-training decreases attentional bias in heavy drinkers without generalization. *Addiction* 2007;102:399-405.
57. Cristea IA, Kok RN, Cuijpers P. The effectiveness of cognitive bias modification interventions for substance addictions: a meta-analysis. *Plos One* 2016;11:e0162226.
58. Cristea IA, Kok RN, Cuijpers P. Efficacy of cognitive bias modification interventions in anxiety and depression: meta-analysis. *Br J Psychiatry* 2015;206:7-16.
59. Mogoaşe C, David D, Koster EH. Clinical efficacy of attentional bias modification procedures: An updated meta-analysis. *Journal of Clinical Psychology* 2014;70:1133-57.
60. Boffo M, Zerhouni O, Gronau QF, et al. Cognitive bias modification for behavior change in alcohol and smoking addiction: Bayesian meta-analysis of individual participant data. *Neuropsychol Rev* 2019;29:52-78.

For peer review only

61. Markland D, Hall CR, Duncan LR, et al. The effects of an imagery intervention on implicit and explicit exercise attitudes. *Psychol Sport Exerc* 2015;17:24-31.
62. Antoniewicz F, Brand R. Learning to like exercising: evaluative conditioning changes automatic evaluations of exercising and influences subsequent exercising behavior. *J Sport Exerc Psychol* 2016;38:138-48.
63. Qiu Y, Zhang G. Make exercise easier: A brief intervention to influence implicit attitudes towards exercise and physical activity behavior. *Learning and Motivation* 2020;72:101660.
64. Cheval B, Sarrazin P, Pelletier L, et al. Effect of retraining approach-avoidance tendencies on an exercise task: A randomized controlled trial. *J Phys Act Health* 2016;13:1396-403.
65. Krieglmeyer R, Deutsch R. Comparing measures of approach–avoidance behaviour: The manikin task vs. two versions of the joystick task. *Cogn Emot* 2010;24:810-28.
66. Chan A-W, Tetzlaff JM, Altman DG, et al. SPIRIT 2013 statement: defining standard protocol items for clinical trials. *Ann Intern Med* 2013;158:200-07.
67. Boot WR, Simons DJ, Stothart C, et al. The pervasive problem with placebos in psychology: Why active control groups are not sufficient to rule out placebo effects. *Perspect Psychol Sci* 2013;8:445-54.
68. Harris PA, Taylor R, Thielke R, et al. A metadata-driven methodology and workflow process for providing translational research informatics support. *J Biomed Inform* 2009;42:377-81.
69. Rougier M, Muller D, Ric F, et al. A new look at sensorimotor aspects in approach/avoidance tendencies: The role of visual whole-body movement information. *J Exp Soc Psychol* 2018;76:42-53.
70. Wiers RW, Rinck M, Kordts R, et al. Retraining automatic action-tendencies to approach alcohol in hazardous drinkers. *Addiction* 2010;105:279-87.
71. Mantri S, Wood S, Duda JE, et al. Comparing self-reported and objective monitoring of physical activity in Parkinson disease. *Parkinsonism Relat Disord* 2019;67:56-59.
72. Bassett DR, Troiano RP, McClain JJ, et al. Accelerometer-based physical activity: total volume per day and standardized measures. *Med Sci Sports Exerc* 2015;47:833-38.
73. Evenson KR, Terry JW. Assessment of differing definitions of accelerometer nonwear time. *Res Q Exerc Sport* 2009;80:355-62.
74. Matthews CE, Ainsworth BE, Thompson RW, et al. Sources of variance in daily physical activity levels as measured by an accelerometer. *Med Sci Sports Exerc* 2002;34:1376-81.
75. Freedson PS, Melanson E, Sirard J. Calibration of the computer science and applications, inc. accelerometer. *Med Sci Sports Exerc* 1998;30:777-81.
76. Boisgontier MP, Cheval B. The anova to mixed model transition. *Neurosci Biobehav Rev* 2016;68:1004-05.
77. Cheval B, Cabral DAR, Daou M, et al. Inhibitory control elicited by physical activity and inactivity stimuli: an EEG study. *Motiv Sci* 2021
78. Cheval B, Daou M, Cabral DAR, et al. Higher inhibitory control is required to escape the innate attraction to effort minimization. *Psychol Sport Exerc* 2020;51:101781.
79. Grimby G, Börjesson M, Jonsdottir I, et al. The "Saltin–Grimby physical activity level scale" and its application to health research. *Scand J Med Sci Spor* 2015;25:119-25.
80. Gosling SD, Rentfrow PJ, Swann Jr WB. A very brief measure of the Big-Five personality domains. *J Res Pers* 2003;37:504-28.
81. Bravell ME, Zarit SH, Johansson B. Self-reported activities of daily living and performance-based functional ability: a study of congruence among the oldest old. *European journal of ageing* 2011;8:199-209.

For peer review only - http://bmjopen.bmj.com/site/about/guidelines.xhtml

82. Leong DP, Teo KK, Rangarajan S, et al. Prognostic value of grip strength: findings from the Prospective Urban Rural Epidemiology (PURE) study. *Lancet* 2015;386:266-73.
83. Mang CS, McEwen LM, MacIsaac JL, et al. Exploring genetic influences underlying acute aerobic exercise effects on motor learning. *Sci Rep* 2017;7:12123.
84. Rhodes RE. The evolving understanding of physical activity behavior: a multi-process action control approach. Advances in Motivation Science: Elsevier 2017:171-205.
85. Craig CL, Marshall AL, Sjostrom M, et al. International physical activity questionnaire: 12-country reliability and validity. *Med Sci Sports Exerc* 2003;35:1381-95.
86. Lowe R, Eves F, Carroll D. The influence of affective and instrumental beliefs on exercise intentions and behavior: A longitudinal analysis. *J Appl Soc Psychol* 2002;32:1241-52.
87. French DP, Sutton S, Hennings SJ, et al. The Importance of Affective Beliefs and Attitudes in the Theory of Planned Behavior: Predicting Intention to Increase Physical Activity 1. *J Appl Soc Psychol* 2005;35:1824-48.

For peer review only

Table 1. Overview of the baseline screening measures

**Measures**	**Assessment method**
**Inclusion criteria**	
Patients treated in ward 3DK of the Division of General Medical	During the first meeting with the research assistant.
≥ 18 years of age	
Can comply with study protocol	
Able to provide a written consent	
**Exclusion criterion**	
Contraindication to PA in the view of the health status	During the first meeting with the research assistant.
**Additional baseline screening assessment**	
Medical evaluation (questionnaires and objective tests)	Patients' diseases and treatment characteristics (medical burden, comorbidity, body mass index, mobility test, functional independence, health-related quality of life).
Sociodemographic characteristics	Questionnaires (age, sex, height, weight).
Usual level of PA	Saltin-Grimby PA Level Scale (SGPALS).[79]
Personality	Ten-Item Personality Inventory (TIPI).[80]
Expectations for improvement	A questionnaire measuring patients' thoughts about the effects of the intervention (three items: "to what extent do you think that your physical activity behaviors will improve as a result of training on the computerized task?"; "to what extent do you think that your mental health will improve as a result of training on the computerized task?"; "to what extent do you think that your physical health will improve as a result of training on the computerized task?").[67]
Self-reported motivation to change	Questionnaire measuring patients' motivation to change their condition (two items: "how motivated are you to change your health condition?"; "to what extent do you really want to change your health condition?"), to avoid a new treatment (two items: "how motivated are you to avoid a new treatment because your health condition?"; "to what extent do you really want to avoid taking a new medication because of your health condition?", and to engage in more PA in the future(two items: "I intend to carry out more physical activity in the next future"; I am determine to carry out more physical activity in the next future").[66]
Self-reported ability to implement daily-life PA	Questionnaire measuring patients' self-reported ability to adopt regular PA in their daily life. Self-reported function in instrumental activities of daily life (IADL; seven items), in activities of daily living (ADL; seven items), and in mobility (three items).[81]

For peer review only - http://bmjopen.bmj.com/site/about/guidelines.xhtml

Table 2. Outcomes measures of the IMPACT trial and assessment time point

Outcome	Assessment method	Measurement timepoints*
**Primary outcomes**		
PA	Accelerometer-based PA (Actigraph GT3X+) to measure the time spent in light, moderate, and vigorous PA.	During the rehabilitation, one week after, as well as one, three, six and 12 months after the intervention.
**Secondary outcomes**		
Automatic precursors of PA		
Approach tendencies	The Visual-Approach/Avoidance-by-the-Self Task (VAAST).[69] A computerized reaction-time task assessing automatic approach tendencies toward PA and sedentary behaviors.	During the rehabilitation, one week after, as well as one, three, six and 12 months after the intervention.
Physical Health		
Weight	Weight (accuracy 0.1 kg) is assessed with participants clothed (lightweight clothing)	During the rehabilitation, one week after, as well as one, three, six and 12 months after the intervention.
Muscle strength	Grip strength measured with a handheld dynamometer.[82]	
Cardiorespiratory fitness	Maximal graded exercise test.[83]	
Perceived global physical health	Global physical health Patient-Reported Outcomes Measurement Information System (PROMIS) scale (one item: "In general, how would you rate your physical health?").	
Mental health		
Perceived physical functioning, fatigue, self-efficacy toward PA	Physical and fatigue PROMIS scales and perceived capability from the Multi-process action control approach,[84] (six items. e.g., "To what extent are you able to carry out your everyday physical activities such as walking, climbing stairs, carrying groceries, or moving a chair?"; "I have the physical ability to walk around the hospital"; "In the past 7 days, how would you rate your fatigue on average?").	During the rehabilitation, one week after, as well as one, three, six and 12 months after the intervention.
Perceived pain interference	Pain interference and pain intensity PROMIS scales (one item: "In the past 7 days, how would you rate your pain on average?")	
Depression, anxiety, general life satisfaction	Anxiety, depression, general life satisfaction PROMIS scales (eight items. e.g., "In general, how would you rate your mental health, including your mood and your ability to think?"; "In general, how would you rate your quality of life?").	
Sleep	Sleep disturbance PROMIS scales (two items: "In the past 7 days, my quality of sleep was…"; "How satisfied/dissatisfied are you with your current sleep?").	
Social role	Ability to participants in social roles and activities PROMIS scale (one item: "In general, how would you rate your satisfaction with social activities and relationships?").	
Other PA-related measures		

For peer review only

Self-reported behaviors	The International PA Questionnaire to measure the time spent in PA and in sedentary behaviors.[85]	During the rehabilitation, one week after, as well as one, three, six and 12 months after the intervention.
Sedentary behaviors	Accelerometer-based sedentary behaviors (Actigraph GT3X+)	
Attitudes	Instrumental (two items: useful, beneficial) and affective (two items: enjoyable, interesting) attitudes toward PA using a short self-reported questionnaire.[86 87]	
Self-reported motivation	Intention (one item: "To what extent do you intend to do physical activities (such as walking in the hospital or in the park) during your rehabilitation?") and importance (one item: "How important is it for you to engage in physical activity during your rehabilitation?").	

Note. The main timepoint of analysis will be the week after the end of intervention

Table 3. Schedule of assessment

WEEK 1	-1 day	0	+1	+2	+3	+4
**Visit**	Information	Screening	1st training session	2nd training session	3rd training session	4th training session
**Oral and written patient information**	+					
**Informed written consent**		+				
**Inclusion exclusion criteria**		+				
**Additional baseline screening assessment**		+				
**Self-reported PA (usual week)**		+				
**Intervention**			+	+	+	+
**Motivation to be active**			+			
**Approach tendencies**			+			
**Physical health**			+			
**Mental health**			+			
**Accelerometer-based PA**			Continuously across the week			

WEEK 2	+1	+2	+3	+4
**Visit**	1st training session	2nd training session	3rd training session	4th training session
**Intervention**	+	+	+	+
**Motivation to be active**	+			
**Approach tendencies**	+			
**Physical health**	+			
**Mental health**	+			
**Accelerometer-based PA**	Continuously across the week			

WEEK 3	+1	+2	+3	+4

For peer review only - http://bmjopen.bmj.com/site/about/guidelines.xhtml

Visit	1st training session	2nd training session	3rd training session	4th training session
**Intervention**	+	+	+	+
**Motivation to be active**	+			
**Approach tendencies**	+			
**Physical health**	+			
**Mental health**	+			
**Accelerometer-based PA**	Continuously across the week			

**Post-intervention**	**1 week**	**1 month**	**3 months**	**6 months**	**12 months**
**Motivation to be active**	+	+	+	+	+
**Approach tendencies**	+	+	+	+	+
**Physical health**	+	+	+	+	+
**Mental health**	+	+	+	+	+
**Self-reported and accelerometer-based PA (during one week)**	+	+	+	+	+

For peer review only

Figure 1. Flow chart

Note. The daily assessment refers to the measure of PA behaviors that will be continuously assessed during the rehabilitation period. The secondary outcomes will be assessed on a weekly basis.

Figure 2. Study design and of Cognitive-Bias Modification (CBM) task

Note. A. Study design. B. Illustration of the Cognitive-Bias Modification (CBM) task. In the CBM task, participants are asked to approach or avoid the picture appearing on the screen depending on its format (i.e., portrait vs. landscape format, counterbalanced across participants). Participants are asked to approach the picture in the approach conditions and to avoid the picture in the avoidance conditions. In the intervention condition, 90% of the pictures depicting physical activity are presented in the approach format (10% in avoidance format), and 90% of the pictures depicting sedentary behaviors are presented in the avoidance format (10% in approach format). In the control condition, the pictures depicting physical activity and sedentary behaviors are equally distributed across formats (i.e., 50%-50%).

For peer review only

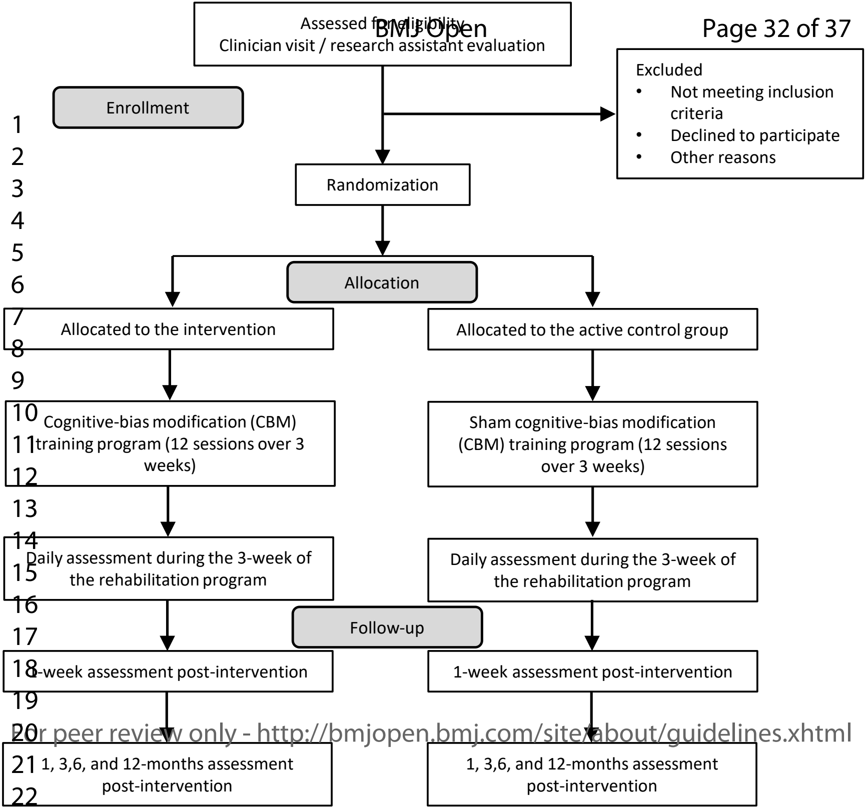

For peer review only - http://bmjopen.bmj.com/site/about/guidelines.xhtml

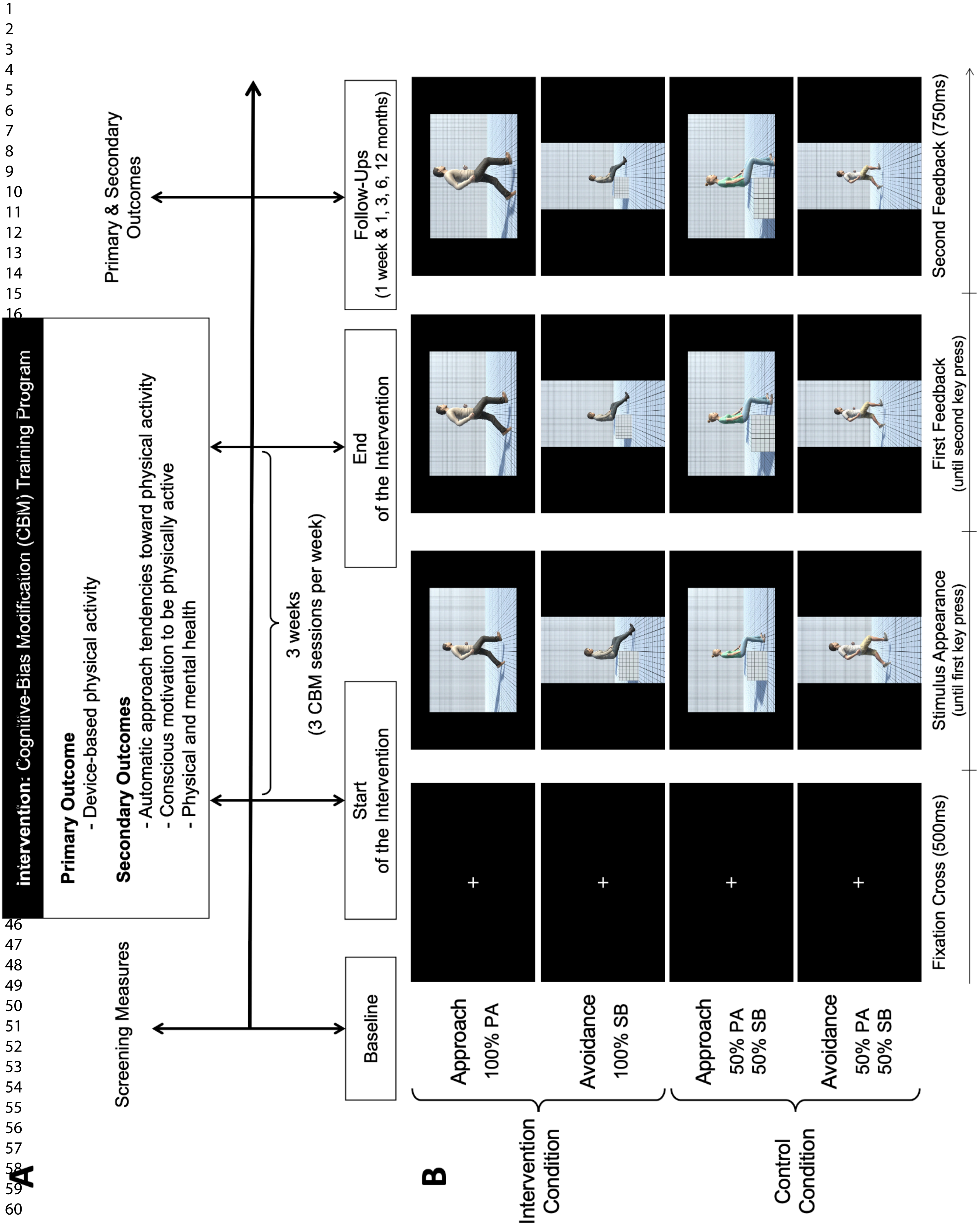
A
Screening Measures
Primary & Secondary Outcomes
Intervention: Cognitive-Bias Modification (CBM) Training Program
Primary Outcome
- Device-based physical activity
Secondary Outcomes
- Automatic approach tendencies toward physical activity
- Conscious motivation to be physically active
- Physical and mental health
3 weeks
(3 CBM sessions per week)
Baseline
Start of the Intervention
End of the Intervention
Follow-Ups (1 week & 1, 3, 6, 12 months)
B
Intervention Condition
Approach 100% PA
Avoidance 100% SB
Control Condition
Approach 50% PA 50% SB
Avoidance 50% PA 50% SB
Fixation Cross (500ms)
Stimulus Appearance (until first key press)
First Feedback (until second key press)
Second Feedback (750ms)

SPIRIT
STANDARD PROTOCOL ITEMS: RECOMMENDATIONS FOR INTERVENTIONAL TRIALS

SPIRIT 2013 Checklist: Recommended items to address in a clinical trial protocol and related documents*

Section/item	Item No	Description	
**Administrative information**			
Title	1	Descriptive title identifying the study design, population, interventions, and, if applicable, trial acronym	Yes, P1
Trial registration	2a	Trial identifier and registry name. If not yet registered, name of intended registry	Yes, P8
	2b	All items from the World Health Organization Trial Registration Data Set	Yes, P8
Protocol version	3	Date and version identifier	Yes, sup. materials
Funding	4	Sources and types of financial, material, and other support	Yes, P19
Roles and responsibilities	5a	Names, affiliations, and roles of protocol contributors	Yes, P19
	5b	Name and contact information for the trial sponsor	Yes, sup. materials
	5c	Role of study sponsor and funders, if any, in study design; collection, management, analysis, and interpretation of data; writing of the report; and the decision to submit the report for publication, including whether they will have ultimate authority over any of these activities	Yes, sup. materials
	5d	Composition, roles, and responsibilities of the coordinating centre, steering committee, endpoint adjudication committee, data management team, and other individuals or groups overseeing the trial, if applicable (see Item 21a for data monitoring committee)	Yes, sup. materials
**Introduction**			
Background and rationale	6a	Description of research question and justification for undertaking the trial, including summary of relevant studies (published and unpublished) examining benefits and harms for each intervention	Yes, P5-p7
	6b	Explanation for choice of comparators	Yes, P11-P12

For peer review only - http://bmjopen.bmj.com/site/about/guidelines.xhtml

Objectives	7	Specific objectives or hypotheses	Yes, P7
Trial design	8	Description of trial design including type of trial (eg, parallel group, crossover, factorial, single group), allocation ratio, and framework (eg, superiority, equivalence, noninferiority, exploratory)	Yes, P7
**Methods: Participants, interventions, and outcomes**			
Study setting	9	Description of study settings (eg, community clinic, academic hospital) and list of countries where data will be collected. Reference to where list of study sites can be obtained	Yes, P8
Eligibility criteria	10	Inclusion and exclusion criteria for participants. If applicable, eligibility criteria for study centres and individuals who will perform the interventions (eg, surgeons, psychotherapists)	Yes, P8-P9
Interventions	11a	Interventions for each group with sufficient detail to allow replication, including how and when they will be administered	Yes, P10-P11
	11b	Criteria for discontinuing or modifying allocated interventions for a given trial participant (eg, drug dose change in response to harms, participant request, or improving/worsening disease)	Yes, P9
	11c	Strategies to improve adherence to intervention protocols, and any procedures for monitoring adherence (eg, drug tablet return, laboratory tests)	Yes, P10
	11d	Relevant concomitant care and interventions that are permitted or prohibited during the trial	Yes, P8
Outcomes	12	Primary, secondary, and other outcomes, including the specific measurement variable (eg, systolic blood pressure), analysis metric (eg, change from baseline, final value, time to event), method of aggregation (eg, median, proportion), and time point for each outcome. Explanation of the clinical relevance of chosen efficacy and harm outcomes is strongly recommended	Yes, P13
Participant timeline	13	Time schedule of enrolment, interventions (including any run-ins and washouts), assessments, and visits for participants. A schematic diagram is highly recommended (see Figure)	Yes, P9
Sample size	14	Estimated number of participants needed to achieve study objectives and how it was determined, including clinical and statistical assumptions supporting any sample size calculations	Yes, P9-P10
Recruitment	15	Strategies for achieving adequate participant enrolment to reach target sample size	Yes, P10
**Methods: Assignment of interventions (for controlled trials)**			
Allocation:			

Sequence generation	16a	Method of generating the allocation sequence (eg, computer-generated random numbers), and list of any factors for stratification. To reduce predictability of a random sequence, details of any planned restriction (eg, blocking) should be provided in a separate document that is unavailable to those who enrol participants or assign interventions	Yes, P13
Allocation concealment mechanism	16b	Mechanism of implementing the allocation sequence (eg, central telephone; sequentially numbered, opaque, sealed envelopes), describing any steps to conceal the sequence until interventions are assigned	Yes, P13
Implementation	16c	Who will generate the allocation sequence, who will enrol participants, and who will assign participants to interventions	Yes, P13
Blinding (masking)	17a	Who will be blinded after assignment to interventions (eg, trial participants, care providers, outcome assessors, data analysts), and how	Yes, P13
	17b	If blinded, circumstances under which unblinding is permissible, and procedure for revealing a participant's allocated intervention during the trial	Yes, P13
**Methods: Data collection, management, and analysis**			
Data collection methods	18a	Plans for assessment and collection of outcome, baseline, and other trial data, including any related processes to promote data quality (eg, duplicate measurements, training of assessors) and a description of study instruments (eg, questionnaires, laboratory tests) along with their reliability and validity, if known. Reference to where data collection forms can be found, if not in the protocol	Yes, P13; Table 1; Table 2
	18b	Plans to promote participant retention and complete follow-up, including list of any outcome data to be collected for participants who discontinue or deviate from intervention protocols	Yes, P10
Data management	19	Plans for data entry, coding, security, and storage, including any related processes to promote data quality (eg, double data entry; range checks for data values). Reference to where details of data management procedures can be found, if not in the protocol	Yes, P10-P16
Statistical methods	20a	Statistical methods for analysing primary and secondary outcomes. Reference to where other details of the statistical analysis plan can be found, if not in the protocol	Yes, P14-p15
	20b	Methods for any additional analyses (eg, subgroup and adjusted analyses)	Yes, P14-p15

	20c	Definition of analysis population relating to protocol non-adherence (eg, as randomised analysis), and any statistical methods to handle missing data (eg, multiple imputation)	Yes, P14-p15
**Methods: Monitoring**			
Data monitoring	21a	Composition of data monitoring committee (DMC); summary of its role and reporting structure; statement of whether it is independent from the sponsor and competing interests; and reference to where further details about its charter can be found, if not in the protocol. Alternatively, an explanation of why a DMC is not needed	Yes, P15-p16
	21b	Description of any interim analyses and stopping guidelines, including who will have access to these interim results and make the final decision to terminate the trial	Yes, P16
Harms	22	Plans for collecting, assessing, reporting, and managing solicited and spontaneously reported adverse events and other unintended effects of trial interventions or trial conduct	Yes, P16
Auditing	23	Frequency and procedures for auditing trial conduct, if any, and whether the process will be independent from investigators and the sponsor	Yes, sup. materials
**Ethics and dissemination**			
Research ethics approval	24	Plans for seeking research ethics committee/institutional review board (REC/IRB) approval	Yes, P17-P18
Protocol amendments	25	Plans for communicating important protocol modifications (eg, changes to eligibility criteria, outcomes, analyses) to relevant parties (eg, investigators, REC/IRBs, trial participants, trial registries, journals, regulators)	Yes, sup. materials
Consent or assent	26a	Who will obtain informed consent or assent from potential trial participants or authorised surrogates, and how (see Item 32)	Yes, P9
	26b	Additional consent provisions for collection and use of participant data and biological specimens in ancillary studies, if applicable	NAN
Confidentiality	27	How personal information about potential and enrolled participants will be collected, shared, and maintained in order to protect confidentiality before, during, and after the trial	Yes, P17
Declaration of interests	28	Financial and other competing interests for principal investigators for the overall trial and each study site	Yes, P20
Access to data	29	Statement of who will have access to the final trial dataset, and disclosure of contractual agreements that limit such access for investigators	Yes, P20

review only

For peer review only - http://bmjopen.bmj.com/site/about/guidelines.xhtml

Ancillary and post-trial care	30	Provisions, if any, for ancillary and post-trial care, and for compensation to those who suffer harm from trial participation	NAN
Dissemination policy	31a	Plans for investigators and sponsor to communicate trial results to participants, healthcare professionals, the public, and other relevant groups (eg, via publication, reporting in results databases, or other data sharing arrangements), including any publication restrictions	Yes, P17
	31b	Authorship eligibility guidelines and any intended use of professional writers	Yes, P20
	31c	Plans, if any, for granting public access to the full protocol, participant-level dataset, and statistical code	Yes, P20
**Appendices**			
Informed consent materials	32	Model consent form and other related documentation given to participants and authorised surrogates	Yes, sup. materials
Biological specimens	33	Plans for collection, laboratory evaluation, and storage of biological specimens for genetic or molecular analysis in the current trial and for future use in ancillary studies, if applicable	NAN

*It is strongly recommended that this checklist be read in conjunction with the SPIRIT 2013 Explanation & Elaboration for important clarification on the items. Amendments to the protocol should be tracked and dated. The SPIRIT checklist is copyrighted by the SPIRIT Group under the Creative Commons "Attribution-NonCommercial-NoDerivs 3.0 Unported" license.

For peer review only

BMJ Open

A cognitive-bias modification intervention to improve physical activity in patients following a rehabilitation program: protocol for the randomized controlled IMPACT trial

Journal:	*BMJ Open*
Manuscript ID	bmjopen-2021-053845.R2
Article Type:	Protocol
Date Submitted by the Author:	03-Sep-2021
Complete List of Authors:	Cheval, Boris; University of Geneva; University of Geneva, Department of Psychology<br>Finckh, Axel; Geneva University Hospitals, Department of Medicine<br>Maltagliati, Silvio; University Grenoble Alpes<br>Fessler, Layan; University Grenoble-Alpes<br>Cullati, Stéphane ; University of Fribourg,<br>Sander, David; University of Geneva; University of Geneva, Department of Psychology<br>Friese, Malte; Saarland University<br>Wiers, Reinout W.; Univ Amsterdam<br>Boisgontier, Matthieu P.; Faculty of Health Sciences University of Ottawa; Bruyère Research Institute<br>Courvoisier, Delphine; Geneva University Hospitals, Division of clinical epidemiology<br>Luthy, C; Division of General Medical Rehabilitation, Geneva University Hospitals & University of Geneva
**Primary Subject Heading**:	Sports and exercise medicine
Secondary Subject Heading:	Rehabilitation medicine
Keywords:	SPORTS MEDICINE, Quality in health care < HEALTH SERVICES ADMINISTRATION & MANAGEMENT, REHABILITATION MEDICINE

SCHOLARONE™
Manuscripts

BMJ

I, the Submitting Author has the right to grant and does grant on behalf of all authors of the Work (as defined in the below author licence), an exclusive licence and/or a non-exclusive licence for contributions from authors who are: i) UK Crown employees; ii) where BMJ has agreed a CC-BY licence shall apply, and/or iii) in accordance with the terms applicable for US Federal Government officers or employees acting as part of their official duties; on a worldwide, perpetual, irrevocable, royalty-free basis to BMJ Publishing Group Ltd ("BMJ") its licensees and where the relevant Journal is co-owned by BMJ to the co-owners of the Journal, to publish the Work in this journal and any other BMJ products and to exploit all rights, as set out in our licence.

The Submitting Author accepts and understands that any supply made under these terms is made by BMJ to the Submitting Author unless you are acting as an employee on behalf of your employer or a postgraduate student of an affiliated institution which is paying any applicable article publishing charge ("APC") for Open Access articles. Where the Submitting Author wishes to make the Work available on an Open Access basis (and intends to pay the relevant APC), the terms of reuse of such Open Access shall be governed by a Creative Commons licence – details of these licences and which Creative Commons licence will apply to this Work are set out in our licence referred to above.

Other than as permitted in any relevant BMJ Author's Self Archiving Policies, I confirm this Work has not been accepted for publication elsewhere, is not being considered for publication elsewhere and does not duplicate material already published. I confirm all authors consent to publication of this Work and authorise the granting of this licence.

For peer review only

A cognitive-bias modification intervention to improve physical activity in patients following a rehabilitation program: protocol for the randomized controlled IMPACT trial

Boris Cheval[1,2], Axel Finckh[3], Silvio Maltagliati[4], Layan Fessler[4], Stéphane Cullati[5,6], David Sander[1,2] Malte Friese[7], Reinout W. Wiers[8], Matthieu P. Boisgontier[9,10], Delphine Courvoisier[11], Christophe Luthy[12]

[1]Swiss Center for Affective Sciences, University of Geneva, Geneva, Switzerland

[2]Laboratory for the Study of Emotion Elicitation and Expression (E3Lab), Department of Psychology, University of Geneva, Geneva, Switzerland

[3]Division of Rheumatology, Department of Medicine, Geneva University Hospitals, Geneva, Switzerland

[4]Univ. Grenoble Alpes, SENS laboratory, Grenoble, France

[5]Population Health Laboratory, University of Fribourg, Switzerland.

[6]Department of Readaptation and Geriatrics, University of Geneva, Switzerland.

[7]Department of Psychology, Saarland University, Germany.

[8]Department of Psychology and center for Urban Mental Health, University of Amsterdam, The Netherlands.

[9]School of Rehabilitation Sciences, Faculty of Health Sciences, University of Ottawa, Ottawa, ON, Canada

[10]Bruyère Research Institute, Ottawa, ON, Canada

[11]Quality of Care Unit, Geneva University Hospitals, Geneva, Switzerland

[12]Division of General Medical Rehabilitation, University Hospitals of Geneva (HUG), Switzerland

For peer review only

For peer review only - http://bmjopen.bmj.com/site/about/guidelines.xhtml

Corresponding author: Campus, Biotech, Chemin des mines 9, 1202, Genève, Switzerland; boris.cheval@unige.ch; @ChevalBoris (B. Cheval).

Word count: 4800

Tables/Figures: 5

For peer review only

Abstract

Introduction. Being physically active is associated with a wide range of health benefits in patients. However, many patients do not engage in the recommended levels of physical activity (PA). To date, interventions promoting PA in patients mainly rely on providing knowledge about the benefits associated with PA to develop their motivation to be active. Yet, these interventions focusing on changing patients' conscious goals have proven to be rather ineffective in changing behaviors. Recent research on automatic factors (e.g., automatic approach tendencies) may provide additional targets for interventions. However, the implementation and evaluation of intervention designed to change these automatic bases of PA are rare. Consequently, little is known about whether and how interventions that target automatically activated processes toward PA can be effective in changing PA behaviors. The Improving physical activity (IMPACT) trial proposes to fill this knowledge gap by investigating the effect of a cognitive-bias modification intervention aiming to modify the automatic approach toward exercise-related stimuli on PA among patients.

Methods and analysis. The IMPACT trial is a single-center, placebo (sham controlled), triple-blinded, phase 3 randomized controlled trial that will recruit 308 patients enrolled in a rehabilitation program in the Division of General Medical Rehabilitation at the University Hospital of Geneva (Switzerland) and intends to follow them up for up to one year after intervention. Immediately after starting a rehabilitation program, patients will be randomized (1:1 ratio) to receive either the cognitive-bias modification intervention consisting of a 12-session training program performed over three weeks or a control condition (placebo). The cognitive-bias modification intervention aims to improve PA levels through a change in automatic approach tendencies toward PA and sedentary behaviors. The primary outcome is the sum of accelerometer-based time spent in light-, moderate-, and vigorous-intensity PA over one week after the cognitive-bias modification intervention (in minutes per week). Secondary outcomes are related to changes in (1) automatic approach tendencies and self-reported motivation to be active, (2) physical health and (3) mental health. Sedentary behaviors and self-reported PA will also be examined. The main timepoint of the analysis will be the week after the end of the intervention. These outcomes will also be assessed during the rehabilitation program, as well as one, three, six, and 12 months after the intervention for secondary analyses.

Ethics and dissemination. The study will be conducted in accordance with the Declaration of Helsinki. This trial was approved by the Ethics Committee of Geneva Canton, Switzerland (reference number: CCER2019-02257). All participants will give an informed consent to

For peer review only

participate in the study. Results will be published in relevant scientific journals and be disseminated in international conferences.

For peer review only

For peer review only - http://bmjopen.bmj.com/site/about/guidelines.xhtml

Strengths and limitations of this study

- The randomized controlled IMPACT trial will test the effects of an intervention based on cognitive-bias modification (CBM) to improve physical activity among patients following a rehabilitation program.

- Physical activity, sedentary behaviors, physical health, and mental health will be measured at multiple time points over one year.

- The findings from this well-powered study will provide evidence-based recommendations for clinical interventions aiming to promote physical activity among patients in rehabilitation.

- The reliance of a single center trial and the selection bias due to loss-to-follow-up and the volunteer participation are keys limitations that may reduce our ability to generalize the results to other populations.

For peer review only

Introduction

The health benefits of physical activity (PA) are well established and extensive. PA can reduce rates of cardiovascular diseases,[1] cancers,[2] hypertension,[3] diabetes,[4] obesity,[5] depression,[6] and all-cause mortality,[7] even more effectively than medication.[8] PA is safe and beneficial for almost everyone, while the risk of harm from moderate PA is small.[8 9] A recent systematic review and meta-analysis suggests that any PA, irrespective of the intensity, is beneficial for health.[7] In patients suffering from chronic diseases, increased PA is associated with reduced hospital admissions, decrease in pain, greater quality of life and mental health, and improvement in physical function.[8 10-13] These myriads of benefits even led the Academy of Medical Sciences to consider PA as a miracle cure.[14] Nevertheless, patients, similarly to the general population, remain largely physically inactive.[15-17]

Healthcare professionals are uniquely placed to promote PA among patients. Today, interventions aiming to enhance PA in patients largely relies on providing rational information about the benefits associated with PA. For example, a practical guide to help clinicians discussing about PA within a consultation has been recently proposed.[8] In this guide, clinicians are encouraged to rationally address patients' concerns about PA, to explain that there are more benefits to become active than to remain sedentary, to set an achievable goal, to identify barriers to be overcome, and finally to set a plan. This type of intervention guide is grounded in the dominant social-cognitive theories,[18] which contend that goals are proximal determinants of behaviors.[19 20] From these perspectives, changing patients' conscious goals should lead to substantial changes in their behaviors.[21 22] While these types interventions have proven to be effective to change PA behaviors to some extent,[23] meta-analyses also indicate that these approaches are more effective in changing intentions than in changing actual behavior.[24] Thus, developing additional interventions targeting alternative mechanisms is needed.

Recent research focusing on automatic mechanisms may provide additional targets for interventions.[25-29] For example, studies showed that in physically active individuals stimuli associated with PA attract attention,[30-33] trigger positive affective reactions,[34-37] and activate approach tendencies toward PA.[38-41] These automatically activated processes are thought to facilitate the translation of conscious goals into actual PA behaviors. Importantly, these automatic reactions predict PA behaviors above and beyond self-reported measures, such as the intention to be physically active[39], and are stronger predictors of spontaneous and unplanned actions that often consist of light-intensity physical activities.[42] As such, from this perspective,

For peer review only - http://bmjopen.bmj.com/site/about/guidelines.xhtml

physical inactivity is thought to also result from an imbalance between a strong motivation to be physically active, but weak automatic approach tendencies toward PA. Crucially, this imbalance between automatic and reflective processes may be particularly pronounced in patients, whose automatic reactions toward PA may be negatively biased by the fear, pain and discomfort felt during some exercises.[43] Thus, in comparison with the general population, patients may demonstrate more negative automatic reactions toward PA, including, for example, stronger negative affective reactions and weaker approach tendencies toward PA. One practical implication of these findings is that interventions designed to promote PA in patients might particularly benefit from directly targeting automatically activated processes toward PA.

What kinds of interventions can target automatically activated processes? New types of interventions have been developed to directly target these automatic reactions toward a given health behavior.[44 45] For example, in alcohol addiction, studies have used a cognitive-bias modification (CBM) intervention aimed at retraining automatic approach reactions toward alcohol using a computerized task.[46] In a CBM intervention, patients were repeatedly asked to push a joystick when exposed to alcohol-related pictures, simulating an avoidance movement. Specifically, in this computerized-based task, participants were asked to push or pull a joystick in response to the format of the pictures. For example, they were instructed to make a pushing movement when the picture presented on the screen was in the landscape format (i.e., avoidance), and to make a pulling movement when the picture was in the portrait format (i.e., approach). To ensure congruence with the participant's actions on the joystick, the picture became smaller when the participant pushed the joystick, and it became larger when the participant pulled the joystick. Participants received training in which they had to push the joystick away in response to pictures of alcohol (i.e., all alcohol pictures were presented in the push format) and to pull the joystick toward them in response to non-alcohol pictures (i.e., all non-alcohol pictures were presented in the pull format). Three large studies conducted in patients showed that adding a CBM intervention to a regular cognitive-behavior treatment yielded a beneficial effect on the relapse rates one year after treatment discharge, with a reduction of 9%,[47] 13%,[46] and 12%,[48] which could be attributed to changes in approach tendencies.[47 49] These interventions have also proven to be useful in impacting cigarette smoking,[50] social anxiety,[51] or eating behaviors.[52-54] Yet, it should be noted, the clinical effectiveness of CBM interventions has been criticized,[55 56] especially for anxiety and depression-related outcomes.[57-60]

For peer review only - http://bmjopen.bmj.com/site/about/guidelines.xhtml

To the best of our knowledge, however, only a handful set of studies have been conducted to target automatic processes toward physical activity.[61-64] Crucially, only one study has been conducted to examine the effect of a brief CBM intervention targeting approach-avoidance tendencies on an exercise task in a sample of healthy young adults.[64] Specifically, using a manikin task,[42 65] a variant of the approach-avoidance joystick task, participants were explicitly trained to repeatedly approach a manikin toward pictures depicting PA and to avoid pictures depicting sedentary behaviors, by pressing keys on the keyboard. Results revealed that participants spent more time exercising during a laboratory exercise task of moderate intensity (i.e., doing squat), in comparison with control groups either trained to approach stimuli depicting sedentary behaviors and avoid stimuli depicting PA (i.e., reverse contingencies) or to approach and avoid stimuli depicting PA and sedentary behaviors equally often (sham controlled). These findings suggest that a single and brief CBM session targeting automatic approach tendencies toward PA and sedentary behaviors can have beneficial effect on laboratory-based PA behaviors. However, this study has at least two important limitations. First, it is unclear if and to what extent the PA behavior performed in the laboratory extends to behaviors performed in everyday life, thereby preventing the possibility to determine whether CMB manipulations can be effective in changing daily-life behaviors. Second, the study was conducted on a sample of rather physically active college students. As such the potential beneficial effect of adding a CBM intervention to a regular treatment in patients, a population which may particularly benefit from such manipulation, remains unknown.

Objectives

In sum, while recent research highlights the importance of targeting automatically activated processes related to PA, the effectiveness of interventions designed to change these presumed automatic bases of PA behaviors has been largely overlooked. Consequently, little is known about whether and how interventions that target automatically activated processes toward PA can be effective in changing behaviors. The primary objective of the IMPACT trial is to investigate the effectiveness of a CBM intervention targeting automatic approach tendencies toward exercise-related stimuli on PA patients in a rehabilitation program. This trial will be performed using a placebo, triple-blinded, phase 3 randomized controlled trial. The secondary objectives are to evaluate the effect of this CBM intervention on changes in (1) automatic approach tendencies and self-reported motivation to be active, (2) physical health and (3) mental health. We hypothesize that the CBM intervention will be associated with higher levels of PA (pre vs. 1-week post intervention) (H1). Moreover, we hypothesize that the CBM

For peer review only - http://bmjopen.bmj.com/site/about/guidelines.xhtml

intervention will increase automatic approach tendencies toward PA (H2a), but will decrease automatic approach tendencies toward sedentary behaviors (H2b). Finally, we predict that the CBM intervention will improve patients' physical and mental health (H3). All these hypotheses will also be tested during the rehabilitation program as well as one, three, six, and 12 months after the intervention (secondary analyses).

Methods and analysis

Study design

The IMPACT trial is a single-center, placebo (sham controlled), triple-blinded, phase 3 randomized controlled trial. The trial will start (First-Participant-In) January 2022 in the ward 3DK of the Division of General Medical Rehabilitation (University Hospitals of Geneva; Switzerland) and will finish (Last-Participant-Out) in January 2024. The ward 3DK admits and manages patients for treatments or diagnostics evaluations, especially after being in acute care for several reasons, such as serious infections, cancer, heart failure, or post-surgery follow-up treatments. This ward offers multidisciplinary treatment in rehabilitation (e.g., physiotherapists, occupational therapists, nutritionists) and does not focus on improving PA engagement. In other words, within the usual care, there is not any content specifically devoted to improve patients' PA level. Eligible patients will be randomly assigned to either the CBM intervention or the active control condition (placebo) in a 1:1 ratio. The current study follows the Standard Protocol Items: Recommendations for Interventional Trials (SPIRIT) statement.[66] The clinical trial was registered at the German clinical trials register (reference number: DRKS00023617).

Eligibility criteria

The eligibility criteria are listed in Box 1. Participants fulfilling all the inclusion criteria are eligible for the study. The presence of the exclusion criterion will lead to the exclusion of the participant.

Box 1. Inclusion and exclusion criteria

Inclusion criteria

- Patients treated in the ward 3DK of the Division of General Medical Rehabilitation
- Aged 18 years or older
- Can comply with the study protocol
- Able to provide a written consent of participation in the trial.

For peer review only - http://bmjopen.bmj.com/site/about/guidelines.xhtml

Exclusion criteria

- Contraindication to PA in the view of the health status

Decision to include/exclude a participant

The decision to include/exclude a participant from this study will be jointly decided by the chief medical officer and the research assistant.

Participant screening, recruitment, and consent

All patients starting rehabilitation program in the ward 3DK of the Division of General Medical Rehabilitation, University Hospital of Geneva, Switzerland (from January 2022 to January 2024) will be approached during the first consultation with the chief medical officer and will receive an information sheet explaining the main objective of the IMPACT trial. The investigators will explain to each participant the nature of the study, its purpose, the procedures involved, the expected duration, the potential risks and benefits and any discomfort it may entail. Each participant will be informed that the participation in the study is voluntary and that he or she may withdraw from the study at any time and that withdrawal of consent will not affect his or her subsequent medical assistance and treatment. The participant will be informed that his or her medical records may be examined by authorized individuals other than their treating physician. All participants will be provided a participant information sheet and a consent form describing the study and providing sufficient information for participants to make an informed decision about their participation in the study (see supplementary materials the patient consent form). Participants will have time to carefully read the documents and can give their responses up to 24 hours after having received the documents. The formal consent of a participant, using the approved consent form, will be obtained before the participant is submitted to any study procedure. Participants will then complete a first questionnaire assessing the exclusion and inclusion criteria, as well as other screening measures. All the questionnaires will be assessed electronically using REDCap software. Finally, patients' expectations regarding the effects of the intervention will be assessed.[67] Table 1 provides an overview of all the baseline screening measures available. The study patient flow chart is provided in Figure 1.

Sample size

For power calculation, our intervention implements a between-subject design and random-effects statistical models (i.e., t-tests). The power calculation is based on the primary outcome [i.e., accelerometer-based time spent in light-, moderate-, and vigorous-intensity PA over one

For peer review only - http://bmjopen.bmj.com/site/about/guidelines.xhtml

week after the cognitive-bias modification intervention (in minutes per week)]. Based on estimates of the effect size of interventions targeting automatic approach tendencies (i.e., Cohen's d = 0.41; e.g., a difference of ~30 minutes per week between the intervention and the control group for a pooled standard error of ~75 minutes per week),[68 69] a sample size calculation indicates that a minimum of 252 patients (126 per arm) would be needed to demonstrate efficacy of the intervention on the device-based PA during the week following the intervention, with a probability of committing a type I error < 5% and a probability of committing a type II error < 10%. We expect a loss to follow-up of 10 to 20% at one week after the intervention, and a loss of 30 to 40% over one year. Thus, a minimum of 352 patients will be recruited. Of note, with this sample size, an alpha of .05 and a power of .90, the smallest effect size we could detect is d = .35. Finally, using the expected effect size (i.e., d =.41), an alpha of .05 and a sample size of 352 patients, we obtain a power of .97.

<u>Feasibility</u>

The ward 3DK of the Division of General Medical Rehabilitation has 24 beds and treats on average 40 patients per month. Based on the chief medical officer's experiences and a first presentation of the study to the patients treated in this unit, we expect that 3 patients out of 5 will not agree (for various reasons) to participate in the study, thereby leading to a total of about 24 participants recruited per month. Consequently, we should be able to collect the target sample size in approximately 12-14 months. The average duration of participants hospitalization in the ward 3DK is about three weeks. As such, though this duration can vary between patients (i.e., some patients only stay a few days), this duration allows for the implementation of the whole intervention (i.e., 12-session training program performed over three weeks). Of note, participants who will not complete all the training sessions will still be included in the analysis. Sensibility analyses will be conducted to examine whether the number of completed sessions influence the effects of the intervention. To accelerate and facilitate knowledge dissemination, all articles will be preprinted, and data and code shared on public repositories.

<u>Patients adherence to the IMPACT trial</u>

Patients adherence to the training program (i.e., if the planned training session is completed or not, and why in case of no completion) and to the other measures are documented in an electronic Case Report Form (eCRF) powered by REDCap.[70] To promote patient retention and complete follow-up (i.e., one, three, six and 12 months after the end of the intervention),

For peer review only - http://bmjopen.bmj.com/site/about/guidelines.xhtml

participants will be contacted by phone by a research assistant two weeks before the follow-up measurement. If they do not answer, they will receive up to two additional phone calls this week. If they do not answer, this procedure will be repeated the following week. If they still do not answer, this time of measurement will be considered as missing. Patients with missing data at a given wave, will be contacted for the following waves through the above-mentioned procedure.

Patients who did not answer a given time of measurement, will still be contacted to participate in the following timepoints.

For peer review only

Interventions

All newly admitted patients will attend a meeting organized in the unit. The objective of this meeting will be to present and illustrate the health benefits of PA. Consistent with the recent practical guide to help healthcare professionals promoting PA to patients,[8] research assistants will follow the "Ask-Assess-Advise" structure for discussing PA behavior change in the consultation. Patients will also receive a watch tracking (i.e., polar) during the rehabilitation period and giving personalized feedback on their PA and sedentary behaviors. This procedure aims at increasing their self-reported motivation to be active, thereby allowing to examine the additional effects of the CBM intervention.

Intervention group: Training program of 12 sessions over 3 weeks (i.e., 4 sessions by week on average) using an adapted version of the Visual-Approach/Avoidance-by-the-Self Task (VAAST),[71] a task that have shown to produce large and replicable effects, compared with the manikin task. Specifically, patients will be asked to react to the format (i.e., portrait vs. landscape format) of the pictures depicting PA and of sedentary behaviors by pressing twice the "move forward" or "move backward" key press to approach or avoid the pictures, respectively. Participants will be instructed to approach the picture when it appears in a portrait format, and to avoid it when the picture appears in a landscape format (the rule will be counterbalanced between participants). Of note, unlike the previous study that relied on an explicit instruction task (i.e., participants were asked to respond to the content of the pictures),[64] the current study uses an irrelevant feature task (i.e., participants were asked to respond to the format of the pictures). This irrelevant feature task allows a training without explicit instruction. Congruent with the patient's approach or avoidance response, the whole visual environment will zoom in on the picture to simulate an approach movement and zoom out to simulate an

For peer review only - http://bmjopen.bmj.com/site/about/guidelines.xhtml

avoidance movement. A change by 10% after each key press will be used to give the impression to walk forward or backward as a consequence of the responses. Participants in the intervention group will receive training in which 90% of pictures depicting PA will be presented in the approach format (and 10% in the avoidance format), and 90% pictures depicting sedentary behaviors will be presented in the avoidance format (and 10% in the approach format). This 90/10 split aims to increase the patients blinding to the condition in which they will be assigned. Each training session will consist of 144 trials for a total duration of approximately 10 minutes. At the first session and at the beginning of each week, the training session will be preceded by 96 assessment trials in which the contingency of approaching or avoiding PA or sedentary behaviors will be 50%. Assessment trials will allow to measure patients' automatic approach-avoidance tendencies toward PA and sedentary behaviors (see Figure 2).

Comparator group: Patients in the comparator group (placebo; sham controlled) will not be trained to approach PA and to avoid sedentary behaviors. Specifically, the retraining sessions will also consist of 144 trials, but the task will require an equal number of approach and avoidance responses to both stimuli depicting PA and sedentary behaviors (see Figure 2). The use of a placebo was chosen to ensure that the potential effects of the experimental condition will be attributable to the content of the training program (i.e., learn to systematically approach PA-related stimuli and avoid sedentary behaviors-related stimuli) rather than because of a simple exposition effect (i.e., the fact to be exposed longer to contents related to physical activity and sedentary behaviors).

Stimuli: Stimuli representing PA and sedentary behaviors will be created using the Unity software ®. A set of 195 pictures including 14 avatars (50% women) in either active (walking and running) and inactive posture (sit on a cubicle) will be tested in a pilot study to identify the 48 pictures the most associated with "movement and physically active behaviors" and the 48 pictures the most associated with "rest and physically inactive behaviors" using two visual analogic scales (VAS 1; *"please indicate how this image is, in your opinion, associated with a behavior that requires: 0 = no physical exertion at all, 100 = a lot of physical exertion*"; VAS 2; *"Please indicate how closely this image is associated with: 0 = resting, sedentary behavior, 100 = movement, very active behavior").* The credibility of the pictures will also be tested ("*how realistic do you think this person's behavior is? Realistic meaning that the images may resemble to a real-life behavior*"; on a VAS from 0 = behavior not at all realistic; 100 = behavior very realistic) and for agreeableness *("how pleasant/sympathetic do you find the person in this*

For peer review only - http://bmjopen.bmj.com/site/about/guidelines.xhtml
For peer review only

image? For example, would you like to talk to her/him"; from 0 = very unpleasant/antipathetic, 100 = very pleasant/sympathetic). The aim of this pilot study was twofold. First, to ensure that the selected pictures reflect the concept of interest (i.e., movement and physical activity vs. rest and physical inactivity). Second, to check that the selected pictures were equivalent in term of credibility and agreeableness across categories (i.e., movement vs. rest). Pictures will be built to match for color, brightness, and visual complexity. To examine the generalization of training effects,[72] in both the intervention and comparator group, only half of the pictures used in the assessment phase will be included on the training phase (the selected pictures will be counterbalanced across participants).

<u>Randomization and blinding</u>

The research assistants and the participants will be blinded to the allocation of the groups. At the end of the trial, the success of the participant blinding will be examined by asking the participants to guess in what group there were, including a percentage of certainty. Moreover, the success of research assistants blinding will be examined by asking each research assistant if they were able to detect the group (comparator vs. intervention) when they conducted the data collection.

The randomization will be generated on a computer and will be performed using permuted blocks (size = 8). To ensure that the research team will be blinded to the randomization, an independent co-worker will carry out the randomization. The patient's identification number will be used to determine the sequence of randomization. Patients will be randomized in a 1:1 ratio between the intervention and active control condition. Unblinding is not planned during the trial as we do not see any reasons that would require either the patients or the researchers to know the group in which the patients were allocated. However, if requested by the patients, unblinding is permissible at the end of the trial.

<u>Outcomes</u>

<u>*Primary outcome*</u>

The primary outcome will be the sum of accelerometer-based time spent in light-, moderate-, and vigorous-intensity PA over one week after the cognitive-bias modification intervention (in minutes per week). Following recommendations in patients,[73] a three-axis accelerometer (Actigraph GT3X+; Pensacola, USA) will be used to assess PA. Patients will be given the

For peer review only - http://bmjopen.bmj.com/site/about/guidelines.xhtml

accelerometer and related indications during the first training session. They will be asked to wear the accelerometer for the full week and to return during the next appointment. They will be instructed on how to wear the device (i.e., over the right hip, affixed to an elastic belt, preferably worn under their waistbands). Currently, the waist-mounted Actigraph is the most used device to objectively measure physical activity.[74] One-minute epochs will be used for data analyses and non-wear time will be defined as ≥ 59 consecutive minutes of zero counts. Daily data will be included if the wear time is ≥ 10 waking hours per day [75]. Data will be included if ≥ 4 days met the aforementioned conditions.[76] The time spent in light, moderate, and vigorous PA over the week will be determined through previously validated cut points,[77] in bouts lasting at least 10 min. Then, in the week following the rehabilitation period, participants will be asked to wear the accelerometer for one week. The sum of times spent in light-, moderate-, and vigorous-intensity PA during this period (in minutes per week) will be used as the primary outcome. Of note, because the duration of the rehabilitation period may strongly vary between patients, it is possible that some patients will be still in the hospital after three weeks, while other will leave the service sooner (e.g., at two weeks). As such, to account for this feature and to allow comparisons between patients, the accelerometer will be scheduled to start on the Monday following their discharge from the rehabilitation unit, regardless the lengths of stay in the ward. Finally, participants will be asked to wear the accelerometer for one week at one, three, six, and 12 months post-intervention.

Secondary outcomes

The secondary outcomes will be the changes in (1) automatic approach tendencies and self-reported motivation to be active, (2) physical health, and (3) mental health. Sedentary behaviors and self-reported PA will also be examined. Table 2 provides an overview of all the outcomes measures and Table 3 provides the schedule of assessment.

Data analysis

Primary analyses

Statistical analyses will be performed according to the intention-to-treat (ITT) principle and will abide by the Consolidated Standards of Reporting Trials (CONSORT) guidelines. Analysis will be conducted in a blinded way. We will use mean, standard deviation (SD), median, and range values to summarize the continuous data. The primary outcome (i.e., the time spent in light-, moderate-, and vigorous-intensity PA over one week after the cognitive-bias modification intervention) will be analyzed using multiple linear regressions. Specifically, to

For peer review only - http://bmjopen.bmj.com/site/about/guidelines.xhtml

test H1, we will test whether the patients' PA level during the week after the end of the intervention will be higher in the intervention group relative to the comparator group, after adjustment for covariates (i.e., age, sex, and indicators of the medical evaluation during the screening assessment). To test H2a and H2b, we will test whether patients' automatic approach tendencies toward PA will be higher and patients' automatic approach tendencies toward sedentary behaviors will be lower in the intervention group relative to the comparator group, after adjustment for covariates. Finally, to test H3, we will test whether patients' physical and mental health during the week after the end of the intervention will be higher in the intervention group relative to the comparator group, after adjustment for covariates. Moderator analyses (i.e., for motivation to change, usual level of PA, personality, expectations for improvement) will be conducted.

For peer review only

Secondary analyses

The aforementioned models will be tested at one, three, six, and 12 months after the intervention. Moreover, to examine the effect of the intervention during the rehabilitation period, mixed effects models will be used. These models account for the nested structure of the data (i.e., multiple observations within a single participant), thereby providing accurate parameter estimates with acceptable Type I error rates.[78] Moreover, these models do not require an equal number of observations across participants, thereby allowing participants with missing observations to be included in the analyses without the need to impute those missing data. To formally examine the effect of the intervention on the evolution of PA within the rehabilitation period, models will include interaction terms between conditions (intervention group vs. comparator group) and number of days within the rehabilitation program (linear and quadratic). The number of days should be relatively equal between patients (about 21 days) but may differ to some extent (some patients can leave earlier or other later than 21 days). A statistically significant interaction will indicate that the rate of PA change throughout the rehabilitation program would be different across the conditions. The quadratic effect of the number of days will be included to account for potential non-linear change of PA across the rehabilitation period. This will allow, for instance, to model the possibility that the effect of the intervention will take some sessions before becoming effective or that no additional effect could be hoped after a certain number of sessions. The continuous secondary outcomes will be treated in the similar way to the primary outcome. All analyses will be conducted using R software®. Any deviation from the original statistical plan will be described and justified in the final trial report.

For peer review only - http://bmjopen.bmj.com/site/about/guidelines.xhtml

Data security, management, and monitoring

Project data will be handled with uttermost discretion and will be only accessible to authorized personnel who require the data to fulfill their duties within the scope of the research project. On the online Case Report Forms (CRFs) and other specific documents, participants are only identified by a unique participant number. The online CRF will be created using Redcap.

Data recording: The dataset will be accompanied by a README file, which will describe the directory hierarchy and file naming convention. The directory will contain an INFO file describing the experimental protocol used in that experiment. This INFO file will also record any deviation from the protocol and other useful contextual information. This procedure should allow the data to be easily understood by other researchers and should support future reuse of the data. Metadata will be created to provide contextual information required to interpret data. This metadata file will be created in accordance with the Data Documentation Initiative (DDI). In particular, the metadata file will include short unique identifier, the name of the author(s), the content, the date of creation, the locations, the reason why the data was generated, and how the data was created. The codebook will explicitly indicate the name, explanations, and the modalities of the different variables measured in the experiment. In addition, it will include information on the study design and contain all information necessary for another analyst to use the data accurately.

Data anonymization: Individual participant information collected during the study is considered confidential and disclosure to third parties is prohibited. Subject confidentiality will be ensured by utilizing subject identification code numbers to correspond to treatment data in the computer files. Only a minority of personnel (i.e., the principal investigator and chief medical officer) will have access to the data in a non-coded form.

Data storage: Participant data on a secure database in accordance with the General Data Protection Regulations (2018). Three copies of the data will be stored. First, original data will be stored on the principal investigator's computer, which will be backed up daily, and protected by a password. Additionally, data will be stored on a secure server hosted by the University of Geneva. Finally, data will be stored on an external device at a different location and be protected by a password. The original notebook will be stored in the principal investigator's laboratory. Local version of the data for statistical analysis will remain on a University computer, and be password protected. Each person who collected the data will have the responsibility to annotate

For peer review only

their data within the metadata. Nevertheless, the principal investigator will have the responsibility to weekly check that the data is properly processed, documented, and stored. All study data will be archived for a minimum of 10 years beyond the end of the randomized controlled trial.

Trial monitoring: The PI will organize a proper training of all involved study personnel to ensure that the study will be conducted according to the protocol. Research assistants should understand the detailed contents of the protocol before starting the data collection. For quality assurance the Ethics Committee may visit the research sites. Direct access to the source data and all project-related files and documents must be granted on such occasions. The principal investigator or any other competent authority may terminate the study prematurely according to the following circumstances: ethical concerns, insufficient participants recruitment, early evidence of harm or benefit of the experimental intervention through the interim analysis planned at six months after the start of the trial. Although no serious adverse event resulting from the intervention is expected, all potential adverse events will be documented within the eCRF.

Patient and public involvement in the trial design

No patient or public was involved in the present study.

Ethics and dissemination

The study was approved by the Ethics Committee of Geneva Canton, Switzerland (reference number: CCER2019-02257). All participants will give an informed consent to participate in the study.

Results will be published in relevant scientific journals and be disseminated in international conferences. Anonymity of the participants will be guaranteed when presenting the data at scientific meetings or publishing them in scientific journals. Individual participant information collected during the study is considered confidential and disclosure to third parties is prohibited.

Data sharing and reuse: Datasets and metadata from this trial will be deposited in ZENODO (a generic and free repository based at CERN, Geneva), and made public at the time of publication. Data in the repository will be stored in accordance with funder and university data policies. Particularly, original datasets, original software script and code, and original raw data

For peer review only

will be deposited. However, as stressed above, personal data will be anonymized before diffusion.

Discussion

PA is associated with a wide range of health benefits,[1-7] but patients, similarly to the general population, remain largely physically inactive. Promoting PA to patients is thus urgently warranted, and healthcare professionals are uniquely placed to do so.[8] To date, interventions mainly rely on providing rational information to change patients' conscious goals and motivation to be active. Yet, these approaches are insufficient to substantially impact actual behaviors.[24] One explanation for this lack of effectiveness draws on recent observations suggesting that automatic reactions toward exercise-related stimuli are involved in the regulation of PA.[33 34 39 79 80] As such, developing interventions targeting both reflective (e.g., motivation) and automatic (e.g., approach tendencies) precursors of PA could be particularly effective. This protocol paper outlines the design of the IMPACT trial, the first placebo, triple-blinded, randomized controlled trial examining the effectiveness of a CBM intervention targeting automatic approach tendencies toward exercise-related stimuli on PA in patients in rehabilitation program remains. The IMPACT trial will focus on an accelerometer-based measure of PA as the primary outcome due to all the extensive benefits associated with being physically active. The secondary outcomes will allow examining other positive-side effects of the intervention on physical and mental health.

The IMPACT randomized controlled trial has several strengths. Firstly, it is the first randomized controlled trial investigating the beneficial effect of an easy deliverable CBM intervention promoting physical activity among patients enrolled in a multidisciplinary rehabilitation program. Secondly, this CBM intervention is anchored within the dual-process models of behavior, arguing that automatic reactions toward physical activity represent additional targets for interventions. Accordingly, this trial will examine for the first time the efficacy of these new types of interventions, which directly targets the automatic precursors of physical activity behavior. Thirdly, we relied on an accelerometer-based measure of physical activity, which guarantee the validity and reliability of our primary outcome. Finally, in addition to physical activity behavior, we will collect data on physical and mental health at multiple time points over one year. However, potential limitations should be noted. The first limitation is related to the fact that the trial is based on a single center, which will limit the generalization of the results to other centers. Second, because of the longitudinal design (i.e.,

For peer review only - http://bmjopen.bmj.com/site/about/guidelines.xhtml

the main time point for the main analysis is assessed four weeks after the start of the intervention and additional time point for secondary analyses are assessed one, three, six, and 12 months after the start of the intervention), we cannot exclude a selection bias due to attrition. Likewise, as participation in our study is voluntary, it may favor the selection of patients with a higher health status or the most motivated to engage in PA. These features are key limitations that may reduce our ability to generalize the results to other populations. Third, to reduce patients' burden, the measure of physical and mental health is based on a single or few items, which may reduce the reliability and validity of these secondary outcomes' measurement. Finally, the rehabilitation program in the Division of General Medical Rehabilitation is a program receiving patients that have been in acute care for different reasons such as serious infections, cancer, heart or lung failure, or post-surgery follow-up treatments. Accordingly, the profiles of the patients included in the trial may strongly differ from one patient to another. Therefore, although patients' profile (e.g., age, sex, or features of the medical evaluation) will be adjusted in the model, the diversity of those profiles may still produce a level of variability likely to influence the effects of the intervention.

PA is a key factor to improve the management of patients' diseases. Helping patients to become more active is likely to promote their recovery, their physical and mental health, as well as to reduce the development of other comorbidities. Targeting automatic reactions toward PA, which may be negatively biased in patients, is particularly innovative. Furthermore, this low cost and easily deliverable intervention could be rapidly implemented on a large scale to help patients become more physically active. The findings from this study will provide evidence-based conclusions for future interventions promoting PA in patients.

For peer review only - http://bmjopen.bmj.com/site/about/guidelines.xhtml

Contributors: B.C.: conceptualization, writing – original draft; A.F.: conceptualization, writing – review & editing; S.M.: Methodology – creation of the tasks, writing – review & editing; L.F.: writing – review & editing; S.C.: writing – review & editing; D.S.: writing – review & editing; M.F.: writing – review & editing; R.W.W.: writing – review & editing; M.P.B.: conceptualization, writing – original draft; D.C.: supervision, resources – provision on instrumentation, writing – review & editing; C.L.: supervision, resources – provision of study materials, writing – original draft

Funding: This work was supported by the Swiss National Science Foundation, grant number PZ00P1_180040. The purchase of protocol-related material (i.e., 30 Polar watches and accelerometers) is provided by the non-operating fund of the Division of General Medical Rehabilitation, University Hospital of Geneva, Switzerland. The computer equipment (3 laptops) will be made available by Division of General Medical Rehabilitation, University Hospital of Geneva. M.P.B. is supported by the Natural Sciences and Engineering Research Council of Canada (RGPIN-2021-03153) and by a grant from The Banting Research Foundation.

Competing interests: None declared

Participants consent: Not obtained

Ethics approval: Ethics Committee of the Geneva University Hospitals, Switzerland

Provenance and peer review: Not commissioned; peer reviewed for ethical and funding approval prior to submission.

Data availability statement: Datasets and metadata from this trial will be deposited in ZENODO (a generic and free repository based at CERN, Geneva) for the duration of 20 years, and made public at the time of publication.

For peer review only

References

1. Wahid A, Manek N, Nichols M, et al. Quantifying the association between physical activity and cardiovascular disease and diabetes: a systematic review and meta-analysis. *J Am Heart Assoc* 2016;5:e002495.
2. Moore SC, Lee I-M, Weiderpass E, et al. Association of leisure-time physical activity with risk of 26 types of cancer in 1.44 million adults. *JAMA Intern Med* 2016;176:816-25.
3. Liu X, Zhang D, Liu Y, et al. Dose–response association between physical activity and incident hypertension: a systematic review and meta-analysis of cohort studies. *Hypertension* 2017;69:813-20.
4. Aune D, Norat T, Leitzmann M, et al. Physical activity and the risk of type 2 diabetes: a systematic review and dose–response meta-analysis. *Eur J Epidemiol* 2015;30:529-42.
5. Bleich SN, Vercammen KA, Zatz LY, et al. Interventions to prevent global childhood overweight and obesity: a systematic review. *Lancet Diabetes Endocrinol* 2018;6:332-46.
6. Schuch F, Vancampfort D, Firth J, et al. Physical activity and sedentary behavior in people with major depressive disorder: a systematic review and meta-analysis. *J Affect Disord* 2017;210:139-50.
7. Ekelund U, Tarp J, Steene-Johannessen J, et al. Dose-response associations between accelerometry measured physical activity and sedentary time and all cause mortality: systematic review and harmonised meta-analysis. *BMJ* 2019;366:l4570.
8. Haseler C, Crooke R, Haseler T. Promoting physical activity to patients. *BMJ* 2019;366:l5230.
9. Godlee F. The miracle cure. *BMJ* 2019;366:l5605.
10. Anderson L, Oldridge N, Thompson DR, et al. Exercise-based cardiac rehabilitation for coronary heart disease: Cochrane systematic review and meta-analysis. *J Am Coll Cardiol* 2016;67:1-12.
11. Stevinson C, Lawlor DA, Fox KR. Exercise interventions for cancer patients: systematic review of controlled trials. *Cancer causes & control* 2004;15:1035-56.
12. Taylor RS, Brown A, Ebrahim S, et al. Exercise-based rehabilitation for patients with coronary heart disease: systematic review and meta-analysis of randomized controlled trials. *The American journal of medicine* 2004;116:682-92.
13. Zanuso S, Jimenez A, Pugliese G, et al. Exercise for the management of type 2 diabetes: a review of the evidence. *Acta Diabetol* 2010;47:15-22.
14. Exercise: the miracle cure and the role of the doctor in promoting it. https://www.aomrc.org.uk/wp-content/uploads/2016/05/Exercise_the_Miracle_Cure_0215.pdf. 2015
15. Johansen KL, Chertow GM, Ng AV, et al. Physical activity levels in patients on hemodialysis and healthy sedentary controls. *Kidney Int* 2000;57:2564-70.
16. Watz H, Waschki B, Meyer T, et al. Physical activity in patients with COPD. *Eur Respir J* 2009;33:262-72.
17. Jeong S-W, Kim S-H, Kang S-H, et al. Mortality reduction with physical activity in patients with and without cardiovascular disease. *Eur Heart J* 2019;40:3547-55.
18. Rhodes RE, Nigg CR. Advancing physical activity theory: a review and future directions. *Exerc Sport Sci Rev* 2011;39:113-19.
19. Bandura A. Self-efficacy: The exercise of control (1st edition). New York: Worth Publishers 1997.
20. Carver CS, Scheier MF. On the self-regulation of behavior. New York: Cambridge University Press 1998.

For peer review only

21. Sheeran P, Gollwitzer PM, Bargh JA. Nonconscious processes and health. *Health Psychol* 2013;32:460-73.
22. Brand R, Cheval B. Theories to explain exercise motivation and physical inactivity: Ways of expanding our current theoretical perspective. *Front Psychol* 2019;10:1147.
23. Hillsdon M, Foster C, Thorogood M. Interventions for promoting physical activity. *Cochrane Database Syst Rev* 2005;25:CD003180.
24. Rhodes RE, Dickau L. Experimental evidence for the intention–behavior relationship in the physical activity domain: a meta-analysis. *Health Psychol* 2012;31:724-27.
25. Cheval B, Radel R, Neva JL, et al. Behavioral and neural evidence of the rewarding value of exercise behaviors: a systematic review. *Sports Med* 2018;48:1389-404.
26. Brand R, Ekkekakis P. Affective–Reflective Theory of physical inactivity and exercise. *Ger J Exerc Sport Res* 2018;48:48-58.
27. Conroy DE, Berry TR. Automatic affective evaluations of physical activity. *Exerc Sport Sci Rev* 2017;45:230-37.
28. Cheval B, Sarrazin P, Boisgontier MP, et al. Temptations toward behaviors minimizing energetic costs (BMEC) automatically activate physical activity goals in successful exercisers. *Psychol Sport Exerc* 2017;30:110-17.
29. Cheval B, Boisgontier MP. The theory of effort minimization in physical activity. *Exerc Sport Sci Rev* 2021
30. Berry TR. Who's even interested in the exercise message? Attentional bias for exercise and sedentary-lifestyle related words. *J Sport Exerc Psychol* 2006;28:4-17.
31. Berry TR, Spence JC, Stolp SM. Attentional bias for exercise-related images. *Res Q Exerc Sport* 2011;82:302-9.
32. Calitri R, Lowe R, Eves FF, et al. Associations between visual attention, implicit and explicit attitude and behaviour for physical activity. *Psychol Health* 2009;24:1105-23.
33. Cheval B, Miller MW, Orsholits D, et al. Physically active individuals look for more: an eye-tracking study of attentional bias. *Psychophysiology* 2020;57:e13582.
34. Conroy DE, Hyde AL, Doerksen SE, et al. Implicit attitudes and explicit motivation prospectively predict physical activity. *Ann Behav Med* 2010;39:112-18.
35. Rebar AL, Ram N, Conroy DE. Using the EZ-diffusion model to score a single-category implicit association test of physical activity. *Psychol Sport Exerc* 2015;16:96-105.
36. Bluemke M, Brand R, Schweizer G, et al. Exercise might be good for me, but I don't feel good about it: do automatic associations predict exercise behavior? *J Sport Exerc Psychol* 2010;32:137-53.
37. Chevance G, Caudroit J, Romain AJ, et al. The adoption of physical activity and eating behaviors among persons with obesity and in the general population: the role of implicit attitudes within the Theory of Planned Behavior. *Psychol Health Med* 2017;22:319-24.
38. Hannan TE, Moffitt RL, Neumann DL, et al. Implicit approach–avoidance associations predict leisure-time exercise independently of explicit exercise motivation. *Sport Exerc Perform Psychol* 2019;8:210-22.
39. Cheval B, Sarrazin P, Isoard-Gautheur S, et al. Reflective and impulsive processes explain (in)effectiveness of messages promoting physical activity: a randomized controlled trial. *Health Psychol* 2015;34:10-19.
40. Cheval B, Sarrazin P, Isoard-Gautheur S, et al. How impulsivity shapes the interplay of impulsive and reflective processes involved in objective physical activity. *Pers Individ Dif* 2016;96:132-7.
41. Cheval B, Tipura E, Burra N, et al. Avoiding sedentary behaviors requires more cortical resources than avoiding physical activity: an EEG study. *Neuropsychologia* 2018;119:68-80.

For peer review only - http://bmjopen.bmj.com/site/about/guidelines.xhtml

42. Cheval B, Sarrazin P, Pelletier L. Impulsive approach tendencies towards physical activity and sedentary behaviors, but not reflective intentions, prospectively predict non-exercise activity thermogenesis. *Plos One* 2014;9:e115238.
43. Shelby RA, Somers TJ, Keefe FJ, et al. Domain specific self-efficacy mediates the impact of pain catastrophizing on pain and disability in overweight and obese osteoarthritis patients. *J Pain* 2008;9:912-19.
44. Friese M, Hofmann W, Wiers RW. On taming horses and strengthening riders: Recent developments in research on interventions to improve self-control in health behaviors. *Self Identity* 2011;10:336-51.
45. Marteau TM, Hollands GJ, Fletcher PC. Changing human behavior to prevent disease: the importance of targeting automatic processes. *Science* 2012;337:1492-95.
46. Wiers RW, Eberl C, Rinck M, et al. Retraining automatic action tendencies changes alcoholic patients' approach bias for alcohol and improves treatment outcome. *Psychol Sci* 2011;22:490-97.
47. Eberl C, Wiers RW, Pawelczack S, et al. Approach bias modification in alcohol dependence: do clinical effects replicate and for whom does it work best? *Dev Cogn Neurosci* 2013;4:38-51.
48. Manning V, Garfield JB, Staiger PK, et al. Effect of cognitive bias modification on early relapse among adults undergoing inpatient alcohol withdrawal treatment: a randomized clinical trial. *JAMA Psychiat* 2021;78:133-40.
49. Gladwin TE, Rinck M, Eberl C, et al. Mediation of cognitive bias modification for alcohol addiction via stimulus-specific alcohol avoidance association. *Alcohol Clin Exp Res* 2015;39:101-07.
50. Wittekind CE, Feist A, Schneider BC, et al. The approach-avoidance task as an online intervention in cigarette smoking: A pilot study. *J Behav Ther Exp* 2015;46:115-20.
51. Taylor CT, Amir N. Modifying automatic approach action tendencies in individuals with elevated social anxiety symptoms. *Behaviour research and therapy* 2012;50:529-36.
52. Fishbach A, Friedman RS, Kruglanski AW. Leading us not unto temptation: Momentary allurements elicit overriding goal activation. *J Pers Soc Psychol* 2003;84:296-309. doi: 10.1037/0022-3514.84.2.296
53. Fishbach A, Shah JY. Self-control in action: implicit dispositions toward goals and away from temptations. *J Pers Soc Psychol* 2006;90:820.
54. Aulbach MB, Knittle K, Haukkala A. Implicit process interventions in eating behaviour: a meta-analysis examining mediators and moderators. *Health Psychol Rev* 2019;13:179-208.
55. Field M, Duka T, Eastwood B, et al. Experimental manipulation of attentional biases in heavy drinkers: do the effects generalise? *Psychopharmacology* 2007;192:593-608.
56. Schoenmakers T, Wiers RW, Jones BT, et al. Attentional re-training decreases attentional bias in heavy drinkers without generalization. *Addiction* 2007;102:399-405.
57. Cristea IA, Kok RN, Cuijpers P. The effectiveness of cognitive bias modification interventions for substance addictions: a meta-analysis. *Plos One* 2016;11:e0162226.
58. Cristea IA, Kok RN, Cuijpers P. Efficacy of cognitive bias modification interventions in anxiety and depression: meta-analysis. *Br J Psychiatry* 2015;206:7-16.
59. Mogoaşe C, David D, Koster EH. Clinical efficacy of attentional bias modification procedures: An updated meta-analysis. *Journal of Clinical Psychology* 2014;70:1133-57.
60. Boffo M, Zerhouni O, Gronau QF, et al. Cognitive bias modification for behavior change in alcohol and smoking addiction: Bayesian meta-analysis of individual participant data. *Neuropsychol Rev* 2019;29:52-78.

For peer review only - http://bmjopen.bmj.com/site/about/guidelines.xhtml

61. Markland D, Hall CR, Duncan LR, et al. The effects of an imagery intervention on implicit and explicit exercise attitudes. *Psychol Sport Exerc* 2015;17:24-31.
62. Antoniewicz F, Brand R. Learning to like exercising: evaluative conditioning changes automatic evaluations of exercising and influences subsequent exercising behavior. *J Sport Exerc Psychol* 2016;38:138-48.
63. Qiu Y, Zhang G. Make exercise easier: A brief intervention to influence implicit attitudes towards exercise and physical activity behavior. *Learning and Motivation* 2020;72:101660.
64. Cheval B, Sarrazin P, Pelletier L, et al. Effect of retraining approach-avoidance tendencies on an exercise task: A randomized controlled trial. *J Phys Act Health* 2016;13:1396-403.
65. Krieglmeyer R, Deutsch R. Comparing measures of approach–avoidance behaviour: The manikin task vs. two versions of the joystick task. *Cogn Emot* 2010;24:810-28.
66. Chan A-W, Tetzlaff JM, Altman DG, et al. SPIRIT 2013 statement: defining standard protocol items for clinical trials. *Ann Intern Med* 2013;158:200-07.
67. Boot WR, Simons DJ, Stothart C, et al. The pervasive problem with placebos in psychology: Why active control groups are not sufficient to rule out placebo effects. *Perspect Psychol Sci* 2013;8:445-54.
68. Treur J, van Dessel P, ter Haar A, et al. Study protocol for ABC-training: a new, personalized cognitive training for smoking cessation. *OSF* 2021.
69. Van Dessel P, Hughes S, De Houwer J. Consequence-based approach-avoidance training: A new and improved method for changing behavior. *Psychol Sci* 2018;29:1899-910.
70. Harris PA, Taylor R, Thielke R, et al. A metadata-driven methodology and workflow process for providing translational research informatics support. *J Biomed Inform* 2009;42:377-81.
71. Rougier M, Muller D, Ric F, et al. A new look at sensorimotor aspects in approach/avoidance tendencies: The role of visual whole-body movement information. *J Exp Soc Psychol* 2018;76:42-53.
72. Wiers RW, Rinck M, Kordts R, et al. Retraining automatic action-tendencies to approach alcohol in hazardous drinkers. *Addiction* 2010;105:279-87.
73. Mantri S, Wood S, Duda JE, et al. Comparing self-reported and objective monitoring of physical activity in Parkinson disease. *Parkinsonism Relat Disord* 2019;67:56-59.
74. Bassett DR, Troiano RP, McClain JJ, et al. Accelerometer-based physical activity: total volume per day and standardized measures. *Med Sci Sports Exerc* 2015;47:833-38.
75. Evenson KR, Terry JW. Assessment of differing definitions of accelerometer nonwear time. *Res Q Exerc Sport* 2009;80:355-62.
76. Matthews CE, Ainsworth BE, Thompson RW, et al. Sources of variance in daily physical activity levels as measured by an accelerometer. *Med Sci Sports Exerc* 2002;34:1376-81.
77. Freedson PS, Melanson E, Sirard J. Calibration of the computer science and applications, inc. accelerometer. *Med Sci Sports Exerc* 1998;30:777-81.
78. Boisgontier MP, Cheval B. The anova to mixed model transition. *Neurosci Biobehav Rev* 2016;68:1004-05.
79. Cheval B, Cabral DAR, Daou M, et al. Inhibitory control elicited by physical activity and inactivity stimuli: an EEG study. *Motiv Sci* 2021
80. Cheval B, Daou M, Cabral DAR, et al. Higher inhibitory control is required to escape the innate attraction to effort minimization. *Psychol Sport Exerc* 2020;51:101781.
81. Grimby G, Börjesson M, Jonsdottir I, et al. The "Saltin–Grimby physical activity level scale" and its application to health research. *Scand J Med Sci Spor* 2015;25:119-25.

For peer review only

82. Gosling SD, Rentfrow PJ, Swann Jr WB. A very brief measure of the Big-Five personality domains. *J Res Pers* 2003;37:504-28.
83. Bravell ME, Zarit SH, Johansson B. Self-reported activities of daily living and performance-based functional ability: a study of congruence among the oldest old. *European journal of ageing* 2011;8:199-209.
84. Leong DP, Teo KK, Rangarajan S, et al. Prognostic value of grip strength: findings from the Prospective Urban Rural Epidemiology (PURE) study. *Lancet* 2015;386:266-73.
85. Mang CS, McEwen LM, MacIsaac JL, et al. Exploring genetic influences underlying acute aerobic exercise effects on motor learning. *Sci Rep* 2017;7:12123.
86. Rhodes RE. The evolving understanding of physical activity behavior: a multi-process action control approach. Advances in Motivation Science: Elsevier 2017:171-205.
87. Craig CL, Marshall AL, Sjostrom M, et al. International physical activity questionnaire: 12-country reliability and validity. *Med Sci Sports Exerc* 2003;35:1381-95.
88. Lowe R, Eves F, Carroll D. The influence of affective and instrumental beliefs on exercise intentions and behavior: A longitudinal analysis. *J Appl Soc Psychol* 2002;32:1241-52.
89. French DP, Sutton S, Hennings SJ, et al. The Importance of Affective Beliefs and Attitudes in the Theory of Planned Behavior: Predicting Intention to Increase Physical Activity 1. *J Appl Soc Psychol* 2005;35:1824-48.

For peer review only

For peer review only - http://bmjopen.bmj.com/site/about/guidelines.xhtml

Table 1. Overview of the baseline screening measures

Measures	Assessment method
**Inclusion criteria**	
Patients treated in ward 3DK of the Division of General Medical	During the first meeting with the research assistant.
≥ 18 years of age	
Can comply with study protocol	
Able to provide a written consent	
**Exclusion criterion**	
Contraindication to PA in the view of the health status	During the first meeting with the research assistant.
**Additional baseline screening assessment**	
Medical evaluation (questionnaires and objective tests)	Patients' diseases and treatment characteristics (medical burden, comorbidity, body mass index, mobility test, functional independence, health-related quality of life).
Sociodemographic characteristics	Questionnaires (age, sex, height, weight).
Usual level of PA	Saltin-Grimby PA Level Scale (SGPALS).[81]
Personality	Ten-Item Personality Inventory (TIPI).[82]
Expectations for improvement	A questionnaire measuring patients' thoughts about the effects of the intervention (three items: "to what extent do you think that your physical activity behaviors will improve as a result of training on the computerized task?"; "to what extent do you think that your mental health will improve as a result of training on the computerized task?"; "to what extent do you think that your physical health will improve as a result of training on the computerized task?").[67]
Self-reported motivation to change	Questionnaire measuring patients' motivation to change their condition (two items: "how motivated are you to change your health condition?"; "to what extent do you really want to change your health condition?"), to avoid a new treatment (two items: "how motivated are you to avoid a new treatment because your health condition?"; "to what extent do you really want to avoid taking a new medication because of your health condition?", and to engage in more PA in the future(two items: "I intend to carry out more physical activity in the next future"; I am determine to carry out more physical activity in the next future").[66]
Self-reported ability to implement daily-life PA	Questionnaire measuring patients' self-reported ability to adopt regular PA in their daily life. Self-reported function in instrumental activities of daily life (IADL; seven items), in activities of daily living (ADL; seven items), and in mobility (three items).[83]

For peer review only

Table 2. Outcomes measures of the IMPACT trial and assessment time point

**Outcome**	**Assessment method**	**Measurement timepoints***
**Primary outcome**		
PA	Accelerometer-based PA (Actigraph GT3X+) to measure the time spent in light-, moderate-, and vigorous-intensity PA over one week.	During the rehabilitation, one week after, as well as one, three, six and 12 months after the intervention.
**Secondary outcomes**		
Automatic precursors of PA		
Approach tendencies	The Visual-Approach/Avoidance-by-the-Self Task (VAAST).[71] A computerized reaction-time task assessing automatic approach tendencies toward PA and sedentary behaviors.	During the rehabilitation, one week after, as well as one, three, six and 12 months after the intervention.
Physical Health		
Weight	Weight (accuracy 0.1 kg) is assessed with participants clothed (lightweight clothing)	During the rehabilitation, one week after, as well as one, three, six and 12 months after the intervention.
Muscle strength	Grip strength measured with a handheld dynamometer.[84]	
Cardiorespiratory fitness	Maximal graded exercise test.[85]	
Perceived global physical health	Global physical health Patient-Reported Outcomes Measurement Information System (PROMIS) scale (one item: "In general, how would you rate your physical health?").	
Mental health		
Perceived physical functioning, fatigue, self-efficacy toward PA	Physical and fatigue PROMIS scales and perceived capability from the Multi-process action control approach,[86] (six items. e.g., "To what extent are you able to carry out your everyday physical activities such as walking, climbing stairs, carrying groceries, or moving a chair?"; "I have the physical ability to walk around the hospital"; "In the past 7 days, how would you rate your fatigue on average?").	During the rehabilitation, one week after, as well as one, three, six and 12 months after the intervention.
Perceived pain interference	Pain interference and pain intensity PROMIS scales (one item: "In the past 7 days, how would you rate your pain on average?")	
Depression, anxiety, general life satisfaction	Anxiety, depression, general life satisfaction PROMIS scales (eight items. e.g., "In general, how would you rate your mental health, including your mood and your ability to think?"; "In general, how would you rate your quality of life?").	
Sleep	Sleep disturbance PROMIS scales (two items: "In the past 7 days, my quality of sleep was…"; "How satisfied/dissatisfied are you with your current sleep?").	
Social role	Ability to participants in social roles and activities PROMIS scale (one item: "In general, how would you rate your satisfaction with social activities and relationships?").	
Other PA-related measures		

For peer review only

For peer review only - http://bmjopen.bmj.com/site/about/guidelines.xhtml

Self-reported behaviors	The International PA Questionnaire to measure the time spent in PA and in sedentary behaviors.[87]	During the rehabilitation, one week after, as well as one, three, six and 12 months after the intervention.
Sedentary behaviors	Accelerometer-based sedentary behaviors (Actigraph GT3X+)	
Attitudes	Instrumental (two items: useful, beneficial) and affective (two items: enjoyable, interesting) attitudes toward PA using a short self-reported questionnaire.[88 89]	
Self-reported motivation	Intention (one item: "To what extent do you intend to do physical activities (such as walking in the hospital or in the park) during your rehabilitation?") and importance (one item: "How important is it for you to engage in physical activity during your rehabilitation?").	

Note. The main timepoint of analysis will be the week after the end of intervention

For peer review only

For peer review only - http://bmjopen.bmj.com/site/about/guidelines.xhtml

Table 3. Schedule of assessment

WEEK 1	-1 day	0	+1	+2	+3	+4
Visit	Information	Screening	1st training session	2nd training session	3rd training session	4th training session
Oral and written patient information	+					
Informed written consent		+				
Inclusion exclusion criteria		+				
Additional baseline screening assessment		+				
Self-reported PA (usual week)		+				
Intervention			+	+	+	+
Motivation to be active			+			
Approach tendencies			+			
Physical health			+			
Mental health			+			
Accelerometer-based PA			Continuously across the week			

WEEK 2	+1	+2	+3	+4
Visit	1st training session	2nd training session	3rd training session	4th training session
Intervention	+	+	+	+
Motivation to be active	+			
Approach tendencies	+			
Physical health	+			
Mental health	+			
Accelerometer-based PA	Continuously across the week			

WEEK 3	+1	+2	+3	+4
Visit	1st training session	2nd training session	3rd training session	4th training session
Intervention	+	+	+	+
Motivation to be active	+			
Approach tendencies	+			
Physical health	+			
Mental health	+			
Accelerometer-based PA	Continuously across the week			

Post-intervention	1 week	1 month	3 months	6 months	12 months
Motivation to be active	+	+	+	+	+
Approach tendencies	+	+	+	+	+
Physical health	+	+	+	+	+
Mental health	+	+	+	+	+

For peer review only

**Self-reported and accelerometer-based PA (during one week)**	+	+	+	+	+	

For peer review only

Figure 1. Flow chart

Note. The daily assessment refers to the measure of PA behaviors that will be continuously assessed during the rehabilitation period. The secondary outcomes will be assessed on a weekly basis.

Figure 2. Study design and of Cognitive-Bias Modification (CBM) task

Note. A. Study design. B. Illustration of the Cognitive-Bias Modification (CBM) task. In the CBM task, participants are asked to approach or avoid the picture appearing on the screen depending on its format (i.e., portrait vs. landscape format, counterbalanced across participants). Participants are asked to approach the picture in the approach conditions and to avoid the picture in the avoidance conditions. In the intervention condition, 90% of the pictures depicting physical activity are presented in the approach format (10% in avoidance format), and 90% of the pictures depicting sedentary behaviors are presented in the avoidance format (10% in approach format). In the control condition, the pictures depicting physical activity and sedentary behaviors are equally distributed across formats (i.e., 50%-50%).

For peer review only

For peer review only - http://bmjopen.bmj.com/site/about/guidelines.xhtml

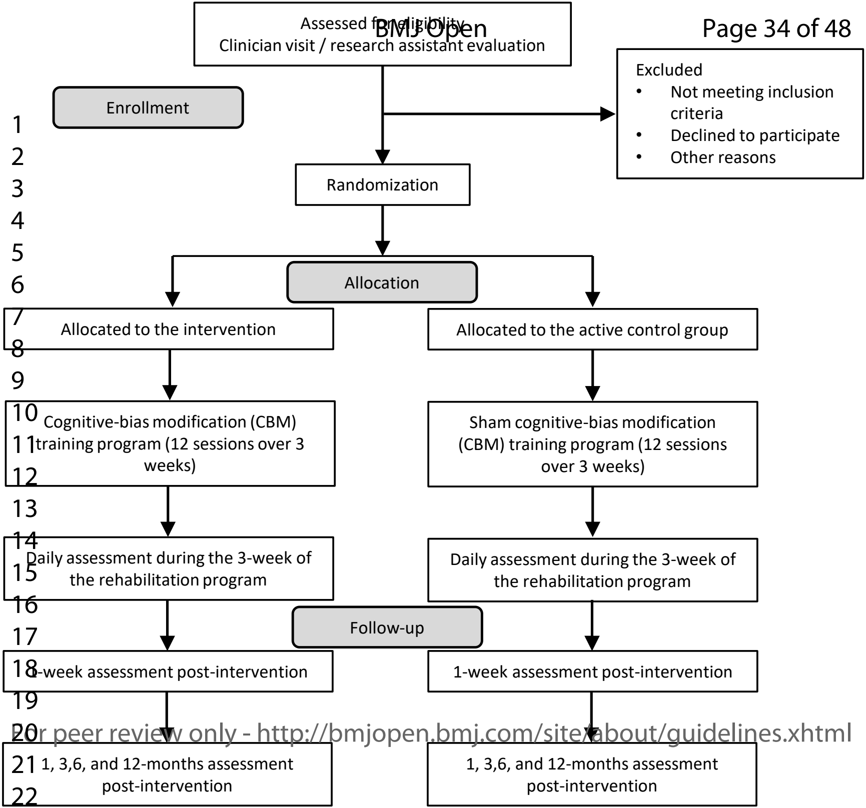
Assessed for eligibility
Clinician visit / research assistant evaluation
Excluded
• Not meeting inclusion criteria
• Declined to participate
• Other reasons
Enrollment
Randomization
Allocation
Allocated to the intervention
Allocated to the active control group
Cognitive-bias modification (CBM) training program (12 sessions over 3 weeks)
Sham cognitive-bias modification (CBM) training program (12 sessions over 3 weeks)
Daily assessment during the 3-week of the rehabilitation program
Daily assessment during the 3-week of the rehabilitation program
Follow-up
1-week assessment post-intervention
1-week assessment post-intervention
1, 3,6, and 12-months assessment post-intervention
1, 3,6, and 12-months assessment post-intervention

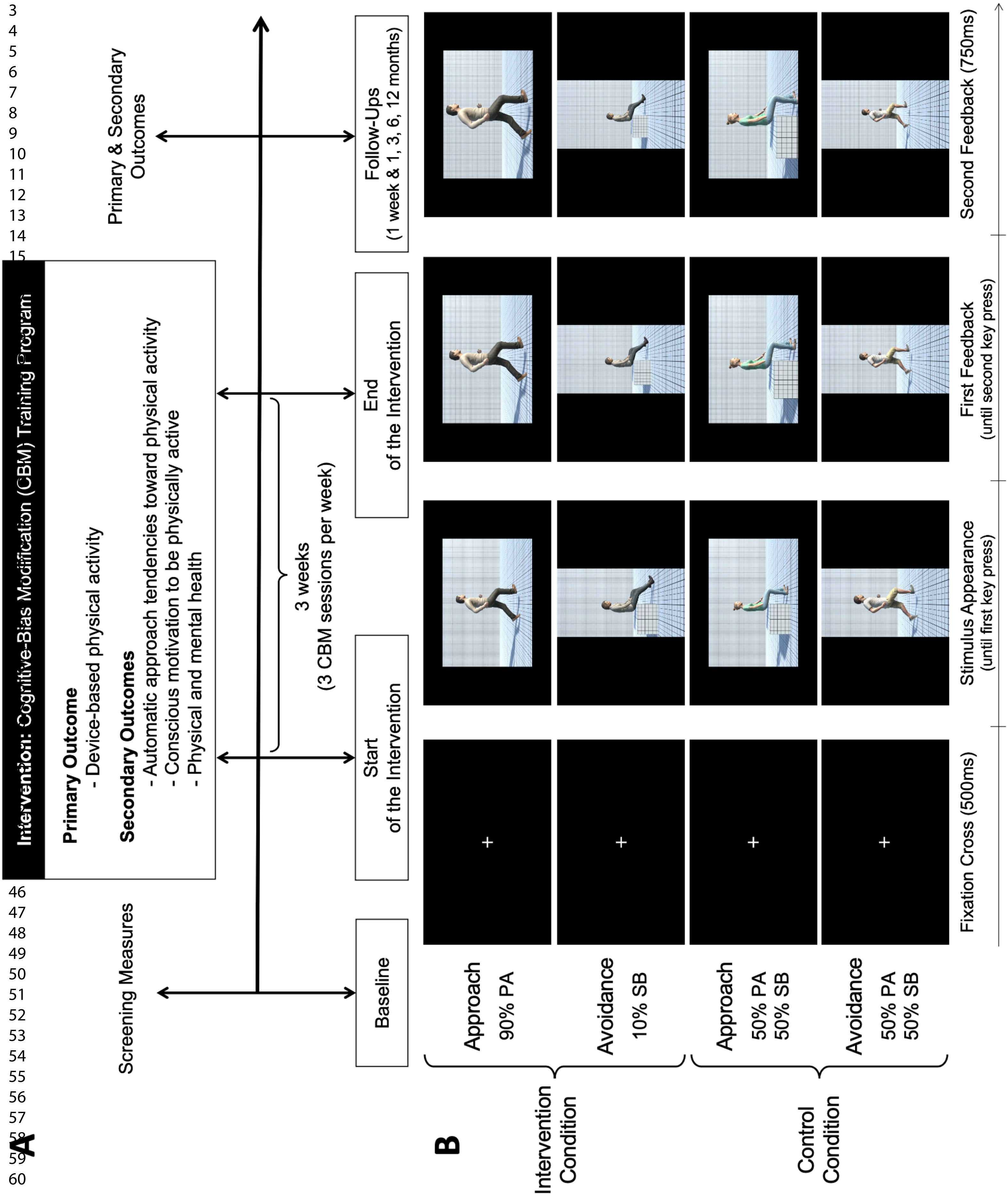
A
Screening Measures
Primary & Secondary Outcomes
Intervention: Cognitive-Bias Modification (CBM) Training Program
Primary Outcome
- Device-based physical activity
Secondary Outcomes
- Automatic approach tendencies toward physical activity
- Conscious motivation to be physically active
- Physical and mental health
3 weeks
(3 CBM sessions per week)
Baseline
Start of the Intervention
End of the Intervention
Follow-Ups
(1 week & 1, 3, 6, 12 months)
B
Intervention Condition
Control Condition
Approach 90% PA
Avoidance 10% SB
Approach 50% PA 50% SB
Avoidance 50% PA 50% SB
Fixation Cross (500ms)
Stimulus Appearance
(until first key press)
First Feedback
(until second key press)
Second Feedback (750ms)

Titre de l'étude

L'étude IMPACT. Promouvoir l'activité physique des patients en reprogrammant leurs réactions automatiques envers l'activité physique à l'aide de jeux sérieux sur ordinateur.

Cette étude est organisée par : Les Hôpitaux Universitaires de Genève et le Centre Interfacultaire en Sciences Affectives (CISA) de l'Université de Genève.

Madame, Monsieur,

Nous vous proposons de participer à notre projet de recherche. Cette feuille d'information décrit le projet de recherche, d'abord dans une version courte (résumé), comme s'il s'agissait d'une table de matières, puis dans une version longue (version détaillée).

Résumé

1	**Objectifs de l'étude**<br>Par la présente, nous vous proposons de participer à notre étude clinique **IMPACT**. Cette étude concerne toutes les personnes qui sont qui sont hospitalisées au 3DK dans le service de médecine interne & réadaptation de l'Hôpital Beau-Séjour. Nous effectuons cette étude pour tester l'efficacité d'une nouvelle intervention visant à promouvoir l'activité physique chez des patients.
2	**Sélection des personnes**<br>La participation est ouverte à toutes les personnes hospitalisées au 3DK nécessitant des traitements de réhabilitation.
3	**Informations générales sur le projet**<br>Cette étude consiste en un programme d'entrainement qui fait appel à une tâche sur ordinateur dite de « jeu sérieux ». Vous serez répartis au hasard dans le groupe qui recevra le jeu sérieux supposé vous aider à devenir plus actif (c'est à dire, le groupe dit « intervention ») ou dans le groupe contrôle que recevra une forme de jeu sérieux factice (c'est à dire, le groupe dit « placebo »). Ni vous, ni la personne en charge de vous faire compléter le programme d'entrainement ne serez au courant du groupe dans lequel vous avez été inclut.<br><br>L'étude débutera début 2020 (après avoir obtenu l'accord du comité d'éthique) et se finira au mois de Janvier 2022 ; elle inclura un total de 250 patients.
4	**Déroulement pour les participants**<br><br><u>Procédure de sélection :</u><br>Dans un premier temps, le médecin responsable de l'étude vous indiquera si vous répondez aux critères d'inclusion de l'étude. La décision sur votre participation (ou non-participation) sera prise conjointement par le principal investigateur et le médecin responsable de l'étude. Un code d'identification créé de façon aléatoire vous sera fourni. Il vous sera demandé de conserver ce code pour les sessions du programme d'entrainement.<br><br><u>Intervention :</u><br>Vous serez invité à participer à une réunion au cours de laquelle vous seront expliqués les bienfaits sur votre santé et votre moral de l'activité physique. Vous aurez aussi l'occasion de discuter avec les médecins de vos appréhensions et barrières que vous pouvez ressentir à l'idée de faire de l'activité physique. Un plan d'activité physique adapté à votre pathologie et à vos attentes vous sera fourni par les professionnels de la santé qui vous prendront en charge. De plus, durant votre séjour, vous serez équipé d'une montre qui

For peer review only - http://bmjopen.bmj.com/site/about/guidelines.xhtml

Hôpitaux Universitaires Genève

UNIVERSITÉ DE GENÈVE
SWISS CENTER FOR AFFECTIVE SCIENCES

	vous permettra d'avoir un retour d'information en continue sur votre niveau d'activité physique et de sédentarité (par exemple, nombre de pas, temps passé assis) au cours de votre programme de réhabilitation.<br><br>L'étude prévoit 15 séances (durée max. de chaque séance 15 min) réparties 3 semaines et au cours desquelles il vous sera demandé de compléter le jeu sérieux. Le groupe intervention recevra la version du jeu sérieux supposé vous aider à devenir plus actif. Le groupe contrôle recevra la version du jeu sérieux factice.<br><br>Pendant toute la durée de l'intervention, vos comportements d'activité physique et de sédentarité seront mesurés en continu à l'aide de la montre qui vous aura été distribuée. À chaque début de semaine, il vous sera aussi demandé de remplir à un questionnaire (durée max. 20 min). Les questions posées nous permettrons de connaître l'évolution de vos capacités physiques et de votre bien être.
5	**Bénéfices pour les participants**<br>La participation à l'intervention devrait vous aider à adopter un style de vie plus actif, et ainsi devrait s'accompagner de bienfaits sur votre santé et l'évolution de vos handicaps.
6	**Droits des participants**<br>Vous êtes libre d'accepter ou de refuser de participer à l'étude. Si vous décidez de ne pas participer, cela ne changera rien à votre prise en charge médicale. Vous n'avez pas à justifier vos décisions.
7	**Obligations des participants**<br>Si vous décidez de participer à l'étude, vous devrez accepter de suivre les instructions et de vous conformer au plan de l'étude.
8	**Risques**<br>Il n'y a pas de risque immédiat associé à cette intervention.
9	**Autres possibilités de traitement**<br>Votre médecin vous conseillera sur les autres possibilités concernant votre traitement.
10	**Découvertes**<br>Toute découverte survenant durant l'étude et pertinente pour votre santé vous sera communiquée. Si vous ne souhaitez pas obtenir ce type d'information, veuillez en avisez le médecin-investigateur.
11	**Confidentialité des données et des échantillons**<br>Nous enregistrerons vos données personnelles (sans géolocalisation). Si vous y consentez (consentement séparé), les données pourront être exploitées dans de futurs projets de recherche. Nous respectons toutes les dispositions légales relatives à la protection des données. Toutes les personnes impliquées sont soumises au secret professionnel. Vos données personnelles et médicales sont protégées et utilisées sous une forme codée.
12	**Retrait de l'étude**<br>Vous pouvez à tout moment vous retirer du projet si vous le souhaitez, sans avoir à vous justifier. Les données recueillies jusque-là seront analysées malgré tout. Cependant, si vous le souhaitez vos données peuvent être détruites. Dans ce cas, toutes les données ainsi que le formulaire de consentement seront détruits.
13	**Compensation des participants**<br>Si vous participez à cette étude, vous ne recevrez pour cela aucune compensation
14	**Réparation des dommages subis**<br>La responsabilité civile des Hôpitaux Universitaires de Genève couvre les dommages éventuels dans le cadre de l'étude<br>L'Université de Genève a souscrit une assurance auprès de AXA Winterthur pour pouvoir réparer les dommages sous sa responsabilité.
15	**Financement de l'étude**

For peer review only - http://bmjopen.bmj.com/site/about/guidelines.xhtml

Hôpitaux Universitaires Genève

UNIVERSITÉ DE GENÈVE
SWISS CENTER FOR AFFECTIVE SCIENCES

	L'étude est financée par le Fond National Suisse de la Recherche Scientifique et les fonds de service du service de médecine interne & réadaptation de l'Hôpital Beau-Séjour des Hôpitaux Universitaire de Genève
16	**Interlocuteur(s)**<br>Christophe Luthy, Prof.<br>4 Rue Gabrielle-Perret-Gentil, 1205 Genève<br>E-mail: Christophe.Luthy@hcuge.ch

For peer review only - http://bmjopen.bmj.com/site/about/guidelines.xhtml

Hôpitaux Universitaires Genève

UNIVERSITÉ DE GENÈVE
SWISS CENTER FOR AFFECTIVE SCIENCES

Information détaillée

1. Objectifs de l'étude

Cette étude doit nous permettre de tester l'efficacité d'une nouvelle intervention visant à promouvoir l'activité physique chez des patients. Plus spécifiquement, nous voulons savoir si un programme d'entrainement basés sur un jeu dit « sérieux » est efficace pour permettre aux patients d'être davantage actif.

2. Sélection des personnes pouvant participer à l'étude

La participation est ouverte à toutes les personnes hospitalisées au 3DK dans le service de médecine interne & réadaptation de l'Hôpital Beau-Séjour et nécessitant des traitements de réhabilitation. Elle est en revanche fermée aux personnes pour qui une réhabilitation n'est pas indiquée.

3. Informations générales sur l'étude

- Cette étude a pour but de tester l'efficacité d'une nouvelle intervention visant à promouvoir l'activité physique chez des patients.
- Cette étude est basée en **Suisse** et toute l'intervention se déroulera au 3DK.
- L'intervention consiste en un programme d'entrainement qui fait appel à une tâche sur ordinateur dit de « jeu sérieux ». Le **programme d'entrainement** sera constitué de 15 séances (durée max. de chaque séance 15 min) réparties 3 semaines et au cours desquelles il vous sera demandé de compléter le « jeu sérieux ».
- L'intervention se déroulera en plusieurs étapes. Dans un premier temps, le médecin responsable de l'étude vous indiquera si vous répondez aux critères d'inclusions de l'étude. Ensuite, vous serez invité à participer à une **réunion** au cours de laquelle il vous sera expliqué les bienfaits de l'activité physique. Pendant cette réunion, une montre permettant de mesurer en continue votre activité physique vous sera prêtée. Vous serez ensuite **aléatoirement** réparti dans le groupe qui recevra le jeu sérieux supposé vous aider à devenir plus actif (c'est à dire, le **groupe dit « intervention »**) ou dans le groupe contrôle que recevra une forme de jeu sérieux factice (c'est à dire, **le groupe dit « placebo »**). Ni vous, ni la personne en charge de vous faire compléter le programme d'entrainement ne serez au courant du groupe dans lequel vous avez été inclus. Au cours de votre programme de réhabilitation, il vous sera aussi demandé de remplir chaque début de semaine à un **questionnaire** (durée max. 20 min) nous permettant de de connaître l'évolution de vos capacités physiques et de votre bien être.
- Au total, la durée de cette étude clinique sera de 2 ans et 250 participants seront inclus.

Nous effectuons cette étude dans le respect des prescriptions de la législation suisse. Nous suivons en outre l'ensemble des directives reconnues au niveau international. La commission cantonale d'éthique compétente a contrôlé et autorisé l'étude.

Vous trouverez aussi un descriptif de l'étude sur le site Internet de l'Office fédéral de la santé publique : www.kofam.ch (le numéro de registre SNCTP de l'étude sera indiqué ici).

4. Déroulement pour les participants

- <u>Procédure de sélection (durée 20 minutes maximum) :</u>

For peer review only - http://bmjopen.bmj.com/site/about/guidelines.xhtml

Dans un premier temps, le médecin responsable de l'étude vous indiquera si vous répondez aux critères d'inclusion de l'étude. La décision sur votre participation (ou non-participation) sera prise conjointement par le principal investigateur de l'étude et le médecin responsable de l'étude. Un code d'identification crée de façon aléatoire vous sera fourni. Il vous sera demandé de conserver ce code pour les sessions du programme d'entrainement.

- <u>Intervention (15 sessions de 15 minutes maximum réparties sur 3 semaines) :</u>

Vous serez invité à participer à une réunion au cours de laquelle vous seront expliqués les aspects positifs de l'activité physique. Vous aurez aussi l'occasion de discuter avec les médecins de vos appréhensions et barrières que vous pouvez ressentir à l'idée de faire de l'activité physique. Un plan d'activité physique adapté à votre pathologie et à vos attentes vous sera fourni par les professionnels de la santé qui vous prendront en charge. De plus, vous recevrez une montre qui vous permettra d'avoir un retour d'information en continue sur votre niveau d'activité physique et de sédentarité (par exemple, nombre de pas, temps passé assis) au cours de votre programme de réhabilitation.

L'étude prévoit 15 séances (durée max. de chaque séance 15 min) réparties 3 semaines et au cours desquelles il vous sera demandé de compléter le « jeu sérieux ». Le groupe intervention recevra la version du jeu sérieux supposé vous aider à devenir plus actif. Le groupe contrôle recevra la version du jeu sérieux factice.

Pendant toute la durée de l'intervention, vos comportements d'activité physique et de sédentarité seront mesurés en continu à l'aide de la montre qui vous aura été distribuée. À chaque début de semaine, il vous sera aussi demandé de remplir à un questionnaire (durée max. 20 min). Les questions posées nous permettrons de connaître l'évolution de vos capacités physiques et de votre bien être.

Il se peut que nous devions vous retirer de l'étude avant le terme prévu. Cette situation peut se produire si nous mettons en évidence une contre-indication. En pareil cas, nous vous proposerons pour votre propre sécurité de vous examiner une dernière fois. Votre médecin traitant sera informé de votre participation à l'étude.

5. Bénéfices pour les participants

Si vous participez à l'étude, cela pourra éventuellement vous aider à adopter un style de vie plus actif, et ainsi devrait s'accompagner de bienfaits sur votre santé et l'évolution de vos handicaps.

Les résultats de l'étude pourraient se révéler importants par la suite pour aider les personnes à être plus actif physiquement.

6. Droits des participants

Votre participation est entièrement libre. Si vous choisissez de ne pas participer ou si vous choisissez de participer et revenez sur votre décision pendant le déroulement de l'étude, vous n'aurez pas à justifier votre refus. Cela ne changera rien à votre prise en charge médicale habituelle. Si vous le souhaitez vos données pourront être détruites. Vous pouvez à tout moment poser toutes les questions nécessaires au sujet de l'étude. Veuillez vous adresser pour ce faire à la personne indiquée à la fin de la présente feuille d'information.

7. Obligations des participants

En tant que participant à l'étude, vous serez tenu :

- de suivre les instructions et de vous conformer au plan de l'étude, à savoir, participer à la première réunion d'information sur les bienfaits de l'activité physique, compléter les 15 séances du programme d'entrainement, porter une montre 24h/24h nous permettant de mesurer votre activité physique en continue, et remplir l'ensemble des questionnaires qui vous seront proposés au cours de l'intervention.

For peer review only - http://bmjopen.bmj.com/site/about/guidelines.xhtml

Hôpitaux Universitaires Genève

UNIVERSITÉ DE GENÈVE
SWISS CENTER FOR AFFECTIVE SCIENCES

8. Risques et contraintes pour les participants

- Il n'y a pas de risque immédiat associé à cette intervention.

9. Autres possibilités de traitement

Vous n'êtes pas tenu de participer à l'étude. Si vous décidez de ne pas y prendre part, il vous sera toujours possible de demander des informations aux médecins concernant les bienfaits de l'activité physique. Vous pourrez aussi participer à la réunion visant à vous expliquer les bienfaits de l'activité physique sans pour autant accepter de participer au programme d'entrainement.

10. Découvertes pendant l'étude

Le médecin-investigateur vous avisera pendant l'étude de toute nouvelle découverte susceptible d'influer sur les bénéfices de l'étude ou votre sécurité, et donc sur votre consentement à participer. Vous serez informé oralement et par écrit.

11. Confidentialité des données et des échantillons

Pour les besoins de l'étude, nous enregistrerons vos données personnelles et médicales. Seul un nombre limité de personnes peut consulter vos données sous une forme non codée, et exclusivement afin de pouvoir accomplir des tâches nécessaires au déroulement du projet. Les données recueillies à des fins de recherche sont codées lors de leur collecte. Le codage signifie que toutes les données permettant de vous identifier (p. ex. le nom, la date de naissance, etc.) sont remplacées par un code (ce code aléatoirement crée vous sera communiqué au début de l'étude. Seuls les responsables auront accès à la base de données permettant de faire le lien entre ce code et votre personne). Le code reste en permanence au sein de l'hôpital. Les personnes ne connaissant pas ce code ne peuvent pas lier ces données à votre personne. Dans le cas d'une publication, les données seront agrégées et personne ne pourra être individuellement identifié. Votre nom n'apparaîtra jamais sur Internet ou dans une publication. Parfois, les journaux scientifiques exigent la transmission de données individuelles (données brutes). Si des données individuelles devaient être transmises, elles seraient toujours codées et ne permettraient donc pas de vous identifier en tant que personne. Toutes les personnes impliquées dans l'étude de quelque manière que ce soit sont tenues au secret professionnel. Toutes les directives relatives à la protection des données sont respectées et vous avez à tout moment le droit de consulter vos données.

Durant son déroulement, l'étude peut faire l'objet d'inspections. Celles-ci peuvent être effectuées par la commission d'éthique qui s'est chargée de son contrôle initial et l'a autorisé, mais aussi être mandatées par l'organisme qui l'a initiée (le fond national suisse pour la recherche scientifique). Il se peut que le médecin-investigateur doive communiquer vos données personnelles et médicales pour les besoins de ces inspections. En cas de dommage, un représentant de l'assurance peut également être amené à consulter vos données. Toutes les personnes sont tenues au secret professionnel.

12. Retrait de d'étude

Vous pouvez à tout moment vous retirer de l'étude si vous le souhaitez, sans avoir besoin de vous justifier.

Les données personnelles recueillies jusque-là seront tout de même analysés, ceci afin de ne pas compromettre la valeur de l'étude dans son ensemble.

13. Compensation des participants

Si vous participez à ce projet, vous ne recevrez pour cela aucune rémunération.

For peer review only - http://bmjopen.bmj.com/site/about/guidelines.xhtml

Hôpitaux Universitaires Genève

UNIVERSITÉ DE GENÈVE
SWISS CENTER FOR AFFECTIVE SCIENCES

14. Réparation des dommages subis

L'organisme ou l'entreprise (promoteur) qui a initié l'étude et est en charge de sa réalisation est responsable des dommages que vous pourriez subir en relation avec la substance à l'étude ou avec les activités de recherche (p.ex. examens). Les conditions et la procédure sont fixées par la loi. L'Université de Genève a conclu une assurance auprès de la compagnie AXA Winterthur pour être en mesure de réparer les dommages relevant de sa responsabilité. La responsabilité civile des Hôpitaux Universitaires de Genève couvre les dommages éventuels dans le cadre de l'étude

15. Financement de l'étude

L'étude est financée par le Fond National Suisse de la Recherche Scientifique les fonds de service du service de médecine interne & réadaptation de l'Hôpital Beau-Séjour des Hôpitaux Universitaire de Genève

16. Interlocuteur(s)

En cas de doute, de craintes ou d'urgences pendant ou après l'étude, vous pouvez vous adresser à tout moment à :

Prof. Christophe Luthy, 4 Rue Gabrielle-Perret-Gentil, 1205 Genève ; E-mail: Christophe.Luthy@hcuge.ch
Boris Cheval, 9, Chemin des Mines, 1202 Genève ; E-mail : boris.cheval@unige.ch

For peer review only

For peer review only - http://bmjopen.bmj.com/site/about/guidelines.xhtml

Hôpitaux Universitaires Genève

UNIVERSITÉ DE GENÈVE
SWISS CENTER FOR AFFECTIVE SCIENCES

Déclaration de consentement

Déclaration de consentement écrite pour la participation à un projet de recherche
Veuillez lire attentivement ce formulaire. N'hésitez pas à poser des questions lorsque vous ne comprenez pas quelque chose ou que vous souhaitez avoir des précisions.

**Numéro BASEC de l'étude:** (après soumission à la commission d'éthique compétente) :	
**Titre de l'étude :** (titre scientifique et titre usuel)	L'étude IMPACT. Promouvoir l'activité physique des patients en reprogrammant leurs réactions automatiques envers l'activité physique à l'aide de jeux sérieux sur ordinateur.
**Institution responsable :** (Promoteur avec adresse complète) :	Hôpitaux Universitaire de Genève / Université de Genève
**Lieu de réalisation de l'étude:**	Hôpital Beau-Séjour
**Médecin responsable du projet sur le site :** (nom et prénom en caractères d'imprimerie) :	LUTHY CHRISTOPHE
**Participant / participante :** (nom et prénom en caractères d'imprimerie) : Date de naissance :	☐ femme ☐ homme

- Je déclare avoir été informé, par le médecin-investigateur responsable de cette étude soussigné ou par un assistant de recherche, oralement et par écrit, des objectifs et du déroulement de l'étude ainsi que des effets présumés, des avantages, des inconvénients possibles et des risques éventuels.
- Je prends part à cette étude de façon volontaire et j'accepte le contenu de la feuille d'information qui m'a été remise sur l'étude précitée. J'ai eu suffisamment de temps pour prendre ma décision.
- J'ai reçu des réponses satisfaisantes aux questions que j'ai posées en relation avec ma participation à l'étude. Je conserve la feuille d'information et reçois une copie de ma déclaration de consentement écrite.
- J'accepte que mon médecin traitant soit informé de ma participation à l'étude.
- J'accepte que les spécialistes compétents du promoteur de l'étude, de la Commission d'éthique compétente puissent consulter mes données brutes afin de procéder à des contrôles, à condition toutefois que la confidentialité de ces données soit strictement assurée.
- Je serai informé des découvertes (fortuites) ayant une incidence directe sur ma santé. Si je ne souhaite pas obtenir ces informations, j'en aviserai le médecin-investigateur.
- Je sais que mes données personnelles peuvent être transmises à des fins de recherche dans le cadre de ce projet uniquement et sous une forme codée.
- Je peux, à tout moment et sans avoir à me justifier, révoquer mon consentement à participer à l'étude, sans que cela n'ait de répercussion défavorable sur la suite de ma prise en charge. Je sais que les données qui ont été recueillies jusque-là seront cependant analysées sauf dans le cas où j'indique ma volonté que les données ainsi que le formulaire de consentement soient totalement détruits.
- Je suis informé que la responsabilité civile de l'hôpital/institution couvre les dommages éventuels que je pourrais subir imputables au projet.

For peer review only - http://bmjopen.bmj.com/site/about/guidelines.xhtml

Hôpitaux Universitaires Genève

UNIVERSITÉ DE GENÈVE
SWISS CENTER FOR AFFECTIVE SCIENCES

- Je suis conscient que les obligations mentionnées dans la feuille d'information destinée aux participants doivent être respectées pendant toute la durée de l'étude. La direction de l'étude peut m'en exclure à tout moment dans l'intérêt de ma santé.

Lieu, date	Signature du participant / de la participante

Attestation du médecin-investigateur : Par la présente, j'atteste avoir expliqué au participant / à la participante la nature, l'importance et la portée de l'étude. Je déclare satisfaire à toutes les obligations en relation avec ce projet conformément au droit en vigueur. Si je devais prendre connaissance, à quelque moment que ce soit durant la réalisation du projet, d'éléments susceptibles d'influer sur le consentement du participant / de la participante à prendre part au projet, je m'engage à l'en informer immédiatement.

Lieu, date	Nom et prénom du médecin-investigateur assurant l'information aux participants en caractères d'imprimerie.<br><br>Signature du médecin-investigateur

For peer review only

For peer review only - http://bmjopen.bmj.com/site/about/guidelines.xhtml

SPIRIT

STANDARD PROTOCOL ITEMS: RECOMMENDATIONS FOR INTERVENTIONAL TRIALS

SPIRIT 2013 Checklist: Recommended items to address in a clinical trial protocol and related documents*

Section/item	Item No	Description	
**Administrative information**			
Title	1	Descriptive title identifying the study design, population, interventions, and, if applicable, trial acronym	Yes, P1
Trial registration	2a	Trial identifier and registry name. If not yet registered, name of intended registry	Yes, P8
	2b	All items from the World Health Organization Trial Registration Data Set	Yes, P8
Protocol version	3	Date and version identifier	Yes, sup. materials
Funding	4	Sources and types of financial, material, and other support	Yes, P19
Roles and responsibilities	5a	Names, affiliations, and roles of protocol contributors	Yes, P19
	5b	Name and contact information for the trial sponsor	Yes, sup. materials
	5c	Role of study sponsor and funders, if any, in study design; collection, management, analysis, and interpretation of data; writing of the report; and the decision to submit the report for publication, including whether they will have ultimate authority over any of these activities	Yes, sup. materials
	5d	Composition, roles, and responsibilities of the coordinating centre, steering committee, endpoint adjudication committee, data management team, and other individuals or groups overseeing the trial, if applicable (see Item 21a for data monitoring committee)	Yes, sup. materials
**Introduction**			
Background and rationale	6a	Description of research question and justification for undertaking the trial, including summary of relevant studies (published and unpublished) examining benefits and harms for each intervention	Yes, P5-p7
	6b	Explanation for choice of comparators	Yes, P11-P12

For peer review only

For peer review only - http://bmjopen.bmj.com/site/about/guidelines.xhtml

Objectives	7	Specific objectives or hypotheses	Yes, P7
Trial design	8	Description of trial design including type of trial (eg, parallel group, crossover, factorial, single group), allocation ratio, and framework (eg, superiority, equivalence, noninferiority, exploratory)	Yes, P7
**Methods: Participants, interventions, and outcomes**			
Study setting	9	Description of study settings (eg, community clinic, academic hospital) and list of countries where data will be collected. Reference to where list of study sites can be obtained	Yes, P8
Eligibility criteria	10	Inclusion and exclusion criteria for participants. If applicable, eligibility criteria for study centres and individuals who will perform the interventions (eg, surgeons, psychotherapists)	Yes, P8-P9
Interventions	11a	Interventions for each group with sufficient detail to allow replication, including how and when they will be administered	Yes, P10-P11
	11b	Criteria for discontinuing or modifying allocated interventions for a given trial participant (eg, drug dose change in response to harms, participant request, or improving/worsening disease)	Yes, P9
	11c	Strategies to improve adherence to intervention protocols, and any procedures for monitoring adherence (eg, drug tablet return, laboratory tests)	Yes, P10
	11d	Relevant concomitant care and interventions that are permitted or prohibited during the trial	Yes, P8
Outcomes	12	Primary, secondary, and other outcomes, including the specific measurement variable (eg, systolic blood pressure), analysis metric (eg, change from baseline, final value, time to event), method of aggregation (eg, median, proportion), and time point for each outcome. Explanation of the clinical relevance of chosen efficacy and harm outcomes is strongly recommended	Yes, P13
Participant timeline	13	Time schedule of enrolment, interventions (including any run-ins and washouts), assessments, and visits for participants. A schematic diagram is highly recommended (see Figure)	Yes, P9
Sample size	14	Estimated number of participants needed to achieve study objectives and how it was determined, including clinical and statistical assumptions supporting any sample size calculations	Yes, P9-P10
Recruitment	15	Strategies for achieving adequate participant enrolment to reach target sample size	Yes, P10
**Methods: Assignment of interventions (for controlled trials)**			
Allocation:			

Sequence generation	16a	Method of generating the allocation sequence (eg, computer-generated random numbers), and list of any factors for stratification. To reduce predictability of a random sequence, details of any planned restriction (eg, blocking) should be provided in a separate document that is unavailable to those who enrol participants or assign interventions	Yes, P13
Allocation concealment mechanism	16b	Mechanism of implementing the allocation sequence (eg, central telephone; sequentially numbered, opaque, sealed envelopes), describing any steps to conceal the sequence until interventions are assigned	Yes, P13
Implementation	16c	Who will generate the allocation sequence, who will enrol participants, and who will assign participants to interventions	Yes, P13
Blinding (masking)	17a	Who will be blinded after assignment to interventions (eg, trial participants, care providers, outcome assessors, data analysts), and how	Yes, P13
	17b	If blinded, circumstances under which unblinding is permissible, and procedure for revealing a participant's allocated intervention during the trial	Yes, P13
**Methods: Data collection, management, and analysis**			
Data collection methods	18a	Plans for assessment and collection of outcome, baseline, and other trial data, including any related processes to promote data quality (eg, duplicate measurements, training of assessors) and a description of study instruments (eg, questionnaires, laboratory tests) along with their reliability and validity, if known. Reference to where data collection forms can be found, if not in the protocol	Yes, P13; Table 1; Table 2
	18b	Plans to promote participant retention and complete follow-up, including list of any outcome data to be collected for participants who discontinue or deviate from intervention protocols	Yes, P10
Data management	19	Plans for data entry, coding, security, and storage, including any related processes to promote data quality (eg, double data entry; range checks for data values). Reference to where details of data management procedures can be found, if not in the protocol	Yes, P10-P16
Statistical methods	20a	Statistical methods for analysing primary and secondary outcomes. Reference to where other details of the statistical analysis plan can be found, if not in the protocol	Yes, P14-p15
	20b	Methods for any additional analyses (eg, subgroup and adjusted analyses)	Yes, P14-p15

For peer review only - http://bmjopen.bmj.com/site/about/guidelines.xhtml

	20c	Definition of analysis population relating to protocol non-adherence (eg, as randomised analysis), and any statistical methods to handle missing data (eg, multiple imputation)	Yes, P14-p15
**Methods: Monitoring**			
Data monitoring	21a	Composition of data monitoring committee (DMC); summary of its role and reporting structure; statement of whether it is independent from the sponsor and competing interests; and reference to where further details about its charter can be found, if not in the protocol. Alternatively, an explanation of why a DMC is not needed	Yes, P15-p16
	21b	Description of any interim analyses and stopping guidelines, including who will have access to these interim results and make the final decision to terminate the trial	Yes, P16
Harms	22	Plans for collecting, assessing, reporting, and managing solicited and spontaneously reported adverse events and other unintended effects of trial interventions or trial conduct	Yes, P16
Auditing	23	Frequency and procedures for auditing trial conduct, if any, and whether the process will be independent from investigators and the sponsor	Yes, sup. materials
**Ethics and dissemination**			
Research ethics approval	24	Plans for seeking research ethics committee/institutional review board (REC/IRB) approval	Yes, P17-P18
Protocol amendments	25	Plans for communicating important protocol modifications (eg, changes to eligibility criteria, outcomes, analyses) to relevant parties (eg, investigators, REC/IRBs, trial participants, trial registries, journals, regulators)	Yes, sup. materials
Consent or assent	26a	Who will obtain informed consent or assent from potential trial participants or authorised surrogates, and how (see Item 32)	Yes, P9
	26b	Additional consent provisions for collection and use of participant data and biological specimens in ancillary studies, if applicable	NAN
Confidentiality	27	How personal information about potential and enrolled participants will be collected, shared, and maintained in order to protect confidentiality before, during, and after the trial	Yes, P17
Declaration of interests	28	Financial and other competing interests for principal investigators for the overall trial and each study site	Yes, P20
Access to data	29	Statement of who will have access to the final trial dataset, and disclosure of contractual agreements that limit such access for investigators	Yes, P20

review only

Ancillary and post-trial care	30	Provisions, if any, for ancillary and post-trial care, and for compensation to those who suffer harm from trial participation	NAN
Dissemination policy	31a	Plans for investigators and sponsor to communicate trial results to participants, healthcare professionals, the public, and other relevant groups (eg, via publication, reporting in results databases, or other data sharing arrangements), including any publication restrictions	Yes, P17
	31b	Authorship eligibility guidelines and any intended use of professional writers	Yes, P20
	31c	Plans, if any, for granting public access to the full protocol, participant-level dataset, and statistical code	Yes, P20
**Appendices**			
Informed consent materials	32	Model consent form and other related documentation given to participants and authorised surrogates	Yes, sup. materials
Biological specimens	33	Plans for collection, laboratory evaluation, and storage of biological specimens for genetic or molecular analysis in the current trial and for future use in ancillary studies, if applicable	NAN

*It is strongly recommended that this checklist be read in conjunction with the SPIRIT 2013 Explanation & Elaboration for important clarification on the items. Amendments to the protocol should be tracked and dated. The SPIRIT checklist is copyrighted by the SPIRIT Group under the Creative Commons "Attribution-NonCommercial-NoDerivs 3.0 Unported" license.

For peer review only
